# Caterpillar Track Complexes in Template-Directed Synthesis and Correlated Molecular Motion**

**DOI:** 10.1002/anie.201412293

**Published:** 2015-02-12

**Authors:** Shiqi Liu, Dmitry V Kondratuk, Sophie A L Rousseaux, Guzmán Gil-Ramírez, Melanie C O'Sullivan, Jonathan Cremers, Tim D W Claridge, Harry L Anderson

**Affiliations:** Department of Chemistry, University of Oxford, Chemistry Research LaboratoryOxford, OX1 3TA (UK)

**Keywords:** molecular gears, molecular motion, porphyrinoids, supramolecular chemistry, templated synthesis

## Abstract

Small alterations to the structure of a star-shaped template totally change its mode of operation. The hexapyridyl template directs the conversion of a porphyrin dimer to the cyclic hexamer, but deleting one pyridine site changes the product to the cyclic decamer, while deleting two binding sites changes the product to the cyclic octamer. This surprising switch in selectivity is explained by the formation of 2:1 caterpillar track complexes, in which two template wheels bind inside the nanoring. Caterpillar track complexes can also be prepared by binding the hexapyridyl template inside the 8- and 10-porphyrin nanorings. NMR exchange spectroscopy (EXSY) experiments show that these complexes exhibit correlated motion, in which the conrotatory rotation of the two template wheels is coupled to rotation of the nanoring track. In the case of the 10-porphyrin system, the correlated motion can be locked by binding palladium(II) dichloride between the two templates.

Template-directed synthesis is a powerful strategy for using reversible non-covalent interactions to control the formation of covalent bonds,[1] and many spectacular examples have been reported recently.[2] We have used oligo-pyridines to direct the synthesis of zinc-porphyrin nanorings,[3] and shown that several small template molecules can cooperate to direct the formation of larger macrocycles, according to a Vernier principle.[4] Here we demonstrate that a single template hub can be modified to direct the formation of several different products, simply by deleting some of the binding sites on the template (Figure 1). Coupling of linear porphyrin dimer **P2** in the presence of the regular *D*_6*h*_ **T6** template gives the cyclic hexamer ***c-*****P6** (Figure 1 a).[3b,d] If two adjacent pyridine binding sites of the **T6** template are deleted to give **T4**, then the main product becomes the cyclic octamer ***c-*****P8** (Figure 1 b), or if only one binding site is deleted, the main product is the cyclic decamer ***c-*****P10** (Figure 1 c). The formation of ***c-*****P10** from **P2** directed by **T5** is a case of Vernier templating, because 10 is the lowest common multiple of 2 and 5, whereas formation of ***c-*****P8** directed by **T4**, illustrates a new type of non-Vernier cooperative templating.[5] We call the 1:2 nanoring template assemblies ***c-*****P8⋅**(**T4**)_**2**_ and ***c-*****P10⋅**(**T5**)_**2**_ “caterpillar track” complexes because of their resemblance to the wheeled treads commonly used on bulldozers, tanks and tractors. In ***c-*****P8⋅**(**T4**)_**2**_ and ***c-*****P10⋅**(**T5**)_**2**_ the template wheels do not rotate, but similar complexes ***c-*****P8⋅**(**T6**)_**2**_ and ***c-*****P10⋅**(**T6**)_**2**_ can be prepared which undergo correlated motion, like a turning caterpillar track.

**Figure 1:**
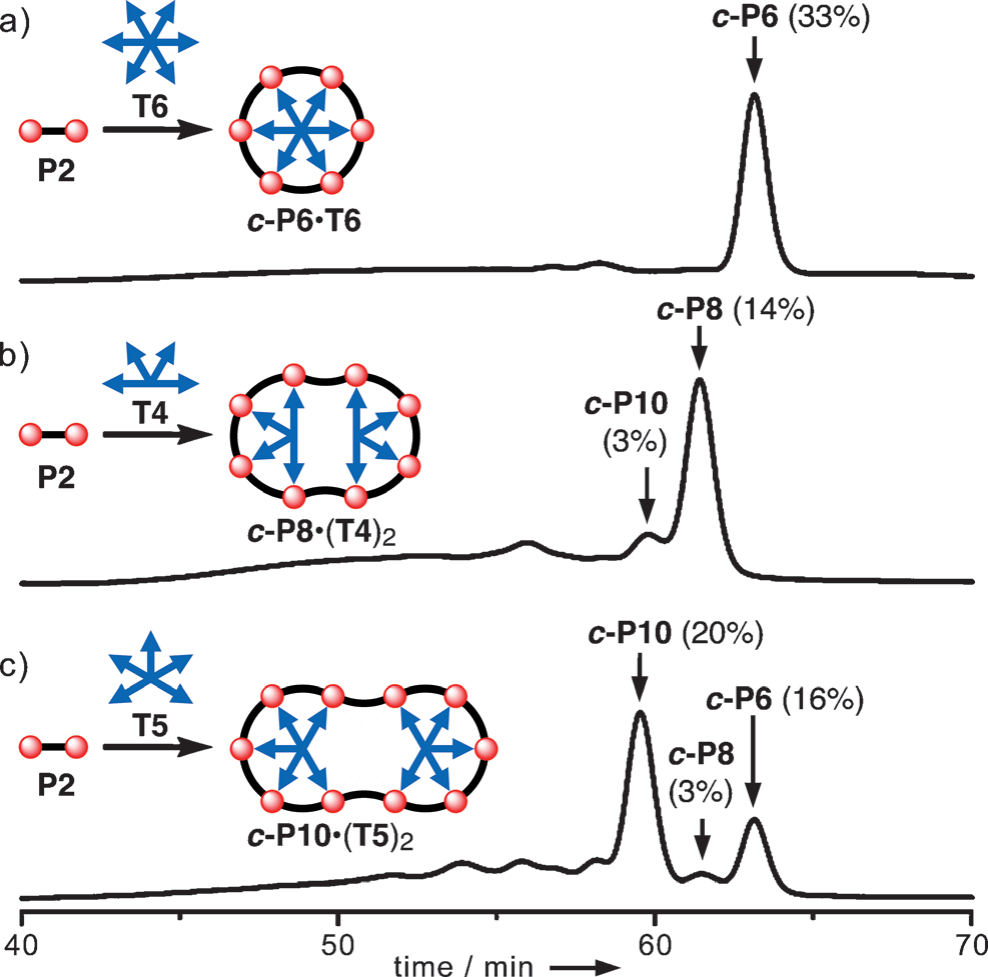
Cartoons showing coupling of a porphyrin dimer (P2) in the presence of templates T6, T4 and T5, together with analytical GPC traces (toluene/1 % pyridine, detection at 500 nm) of the crude reaction mixtures from these three reactions. The detailed chemical structures are shown in Figure 2; the product distributions shown here were obtained using the compounds with R=OC_8_H_17_. The percentages are isolated yields, for the main product of each reaction, and analytical yields for byproducts.

The molecular structures of the compounds used in this study are shown in Figure 2. Despite their structural similarity, templates **T4**, **T5** and **T6** have dramatically different effects on the palladium-catalyzed oxidative coupling of porphyrin dimer **P2**, as seen from the gel permeation chromatography (GPC) traces of crude reaction mixtures in Figure 1. No detectable trace of ***c-*****P8** or ***c-*****P10** is formed when the reaction is carried out in the presence of **T6**, but these larger nanorings are isolated in yields of 14 % and 20 %, respectively, by using templates **T4** and **T5** (with R=OC_8_H_17_, *n*-octyloxy). Similar reactions were also carried out using the version of **P2** with R=*t-*Bu, giving ***c-*****P8** and ***c-*****P10** in isolated yields of 29 % and 18 %, respectively (see Figure S33 in the Supporting Information for GPC traces). Previously we isolated ***c-*****P10** as an unexpected byproduct during the synthesis of ***c-*****P30** by the Vernier coupling of a linear porphyrin decamer in the presence of **T6**;[4a] the formation of a caterpillar track complex now explains how ***c-*****P10** was formed in this reaction.[6]

**Figure 2:**
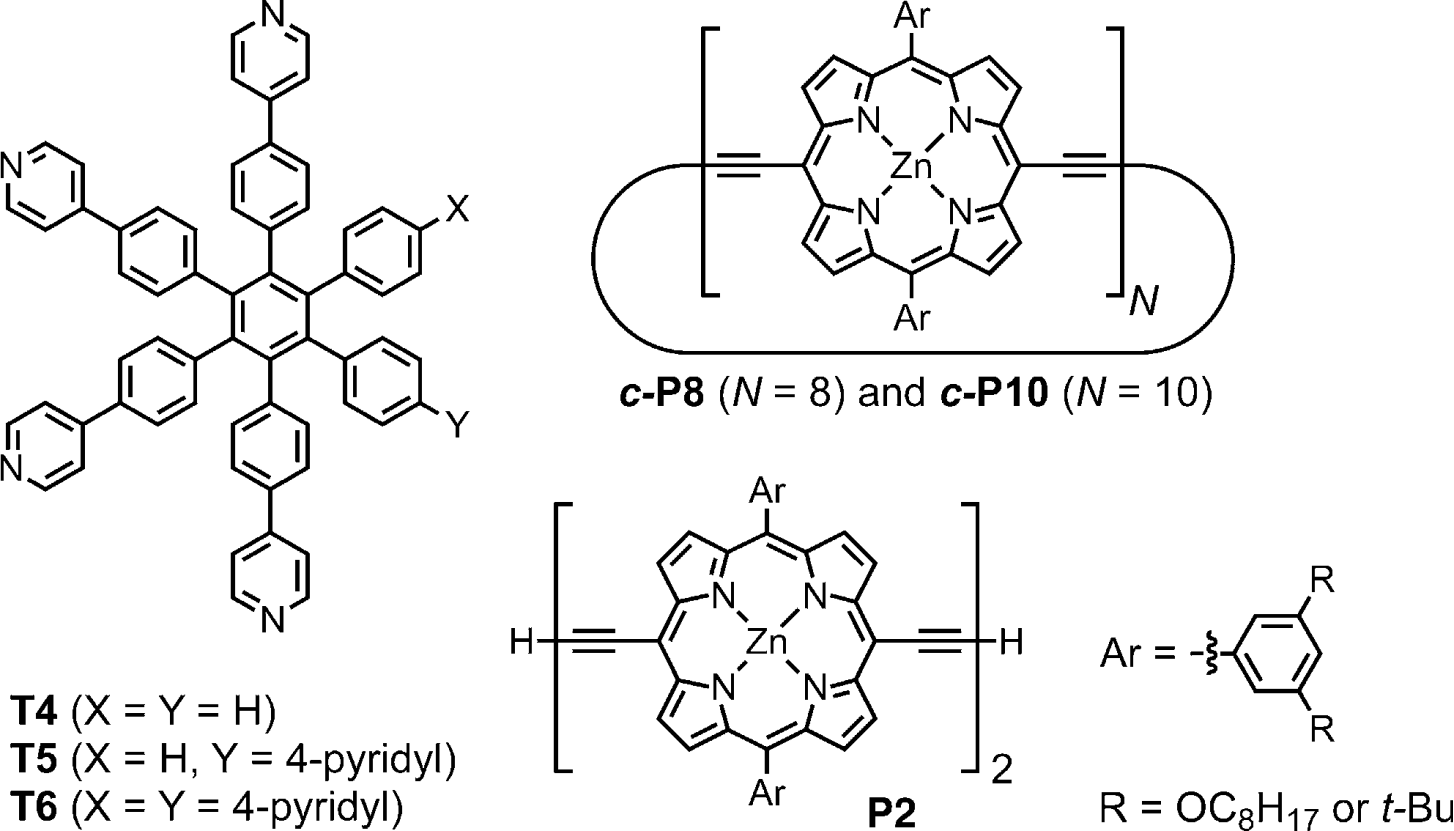
Structures of T4, T5, T6, P2, *c*-P*N*.

The discovery of “caterpillar track” template effects prompted us to investigate the structures and stabilities of the complexes ***c-*****P8⋅**(**T4**)_**2**_ and ***c-*****P10⋅**(**T5**)_**2**_. UV/Vis/NIR and ^1^H NMR titrations showed that 1:2 complexes are formed in solution with high allosteric cooperativity; in both cases, the corresponding 1:1 complexes do not form in detectable concentrations (Figure S35–S37). The nanorings ***c-*****P8** and ***c-*****P10** also form similar 1:2 caterpillar track complexes with the regular **T6** template. The optimized geometries of ***c-*****P8⋅**(**T6**)_**2**_ and ***c-*****P10⋅**(**T6**)_**2**_, from molecular mechanics calculations (Figure 3) are similar to the geometries of ***c-*****P8⋅**(**T4**)_**2**_ and ***c-*****P10⋅**(**T5**)_**2**_ deduced from solution-phase small-angle X-ray scattering (SAXS) studies (Figures S48 and S49). In both structures, all the zinc atoms lie in essentially the same plane, and in ***c-*****P8⋅**(**T6**)_**2**_, the two template units overlap at the center.

**Figure 3:**
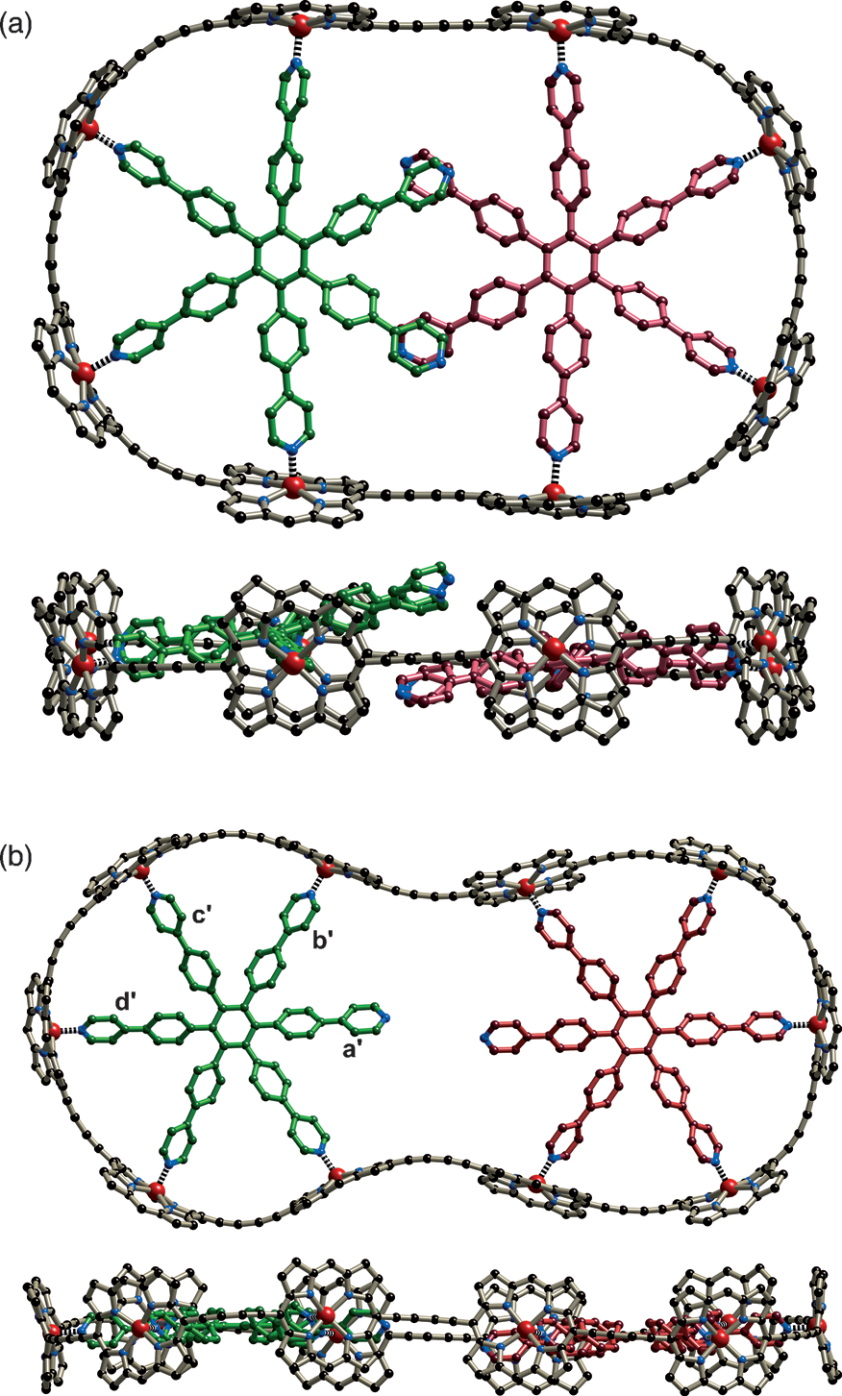
Two orthogonal views of the calculated structures of a) *c*-P8⋅(T6)_2_ and b) *c*-P10⋅(T6)_2_. (MM+ force field, HyperChem; *meso-*aryl side groups were not included in the calculations; hydrogen atoms are omitted for clarity.)

^1^H NMR exchange spectroscopy (EXSY) experiments were carried out to elucidate the dynamics of the complexes ***c-*****P8⋅**(**T4**)_**2**_, ***c-*****P8⋅**(**T6**)_**2**_, ***c-*****P10⋅**(**T5**)_**2**_, and ***c-*****P10⋅**(**T6**)_**2**_. These experiments demonstrated that the two coordinatively saturated complexes, ***c-*****P8⋅**(**T4**)_**2**_ and ***c-*****P10⋅**(**T5**)_**2**_, are static, with no exchange between template environments, whereas the two complexes with free pyridine sites, ***c-*****P8⋅**(**T6**)_**2**_ and ***c-*****P10⋅**(**T6**)_**2**_, undergo caterpillar track motion of the type shown schematically for ***c*****-P8⋅**(**T6**)_**2**_ in Figure 4. The 1D EXSY spectra in Figure 5 demonstrate this concerted motion by revealing exchange signals only between environments that are related by a 60° rotation of the template (***m***⇔—***a***, ***a***⇔—***b***, ***l***⇔—***c***, and ***c***⇔—***d***); signals corresponding to a rotation of 120° (***m***⇔—***b*** and ***l***⇔—***d***) are absent on the time-scale of the experiment, confirming that the observed exchange peaks are due to intramolecular rotation of bound **T6**, and not due to random exchange of the template units. Under the same conditions, an identical 1D EXSY experiment on ***c*****-P8⋅**(**T4**)_**2**_ showed no exchange signals between template protons (Figure S43), confirming that the exchange peaks observed in ***c*****-P8⋅**(**T6**)_**2**_ result from intramolecular motion.

**Figure 4:**
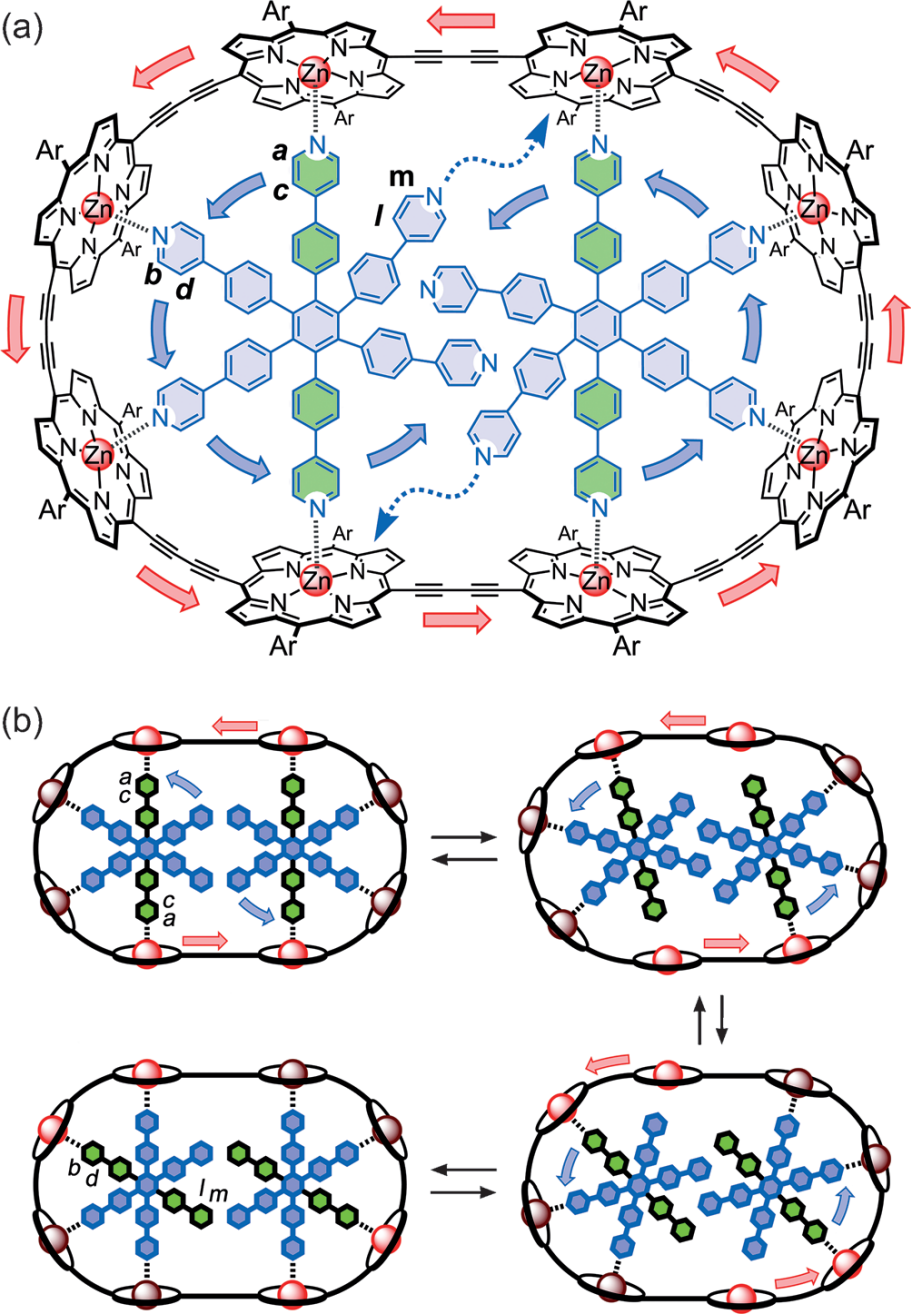
Caterpillar track motion in *c*-P8⋅(T6)_2_, as revealed by ^1^H NMR EXSY experiments. a) Detailed molecular structure. b) Mechanism leading to a concerted 60° rotation of both template units, transforming environments a and c into b/m and d/l. The time constant for this 60° rotation is 7.7 s. According to this mechanism, both template units must rotate in the same direction, but the direction of rotation is arbitrary, and subsequent 60° rotation steps could occur in either direction with equal probability.

**Figure 5:**
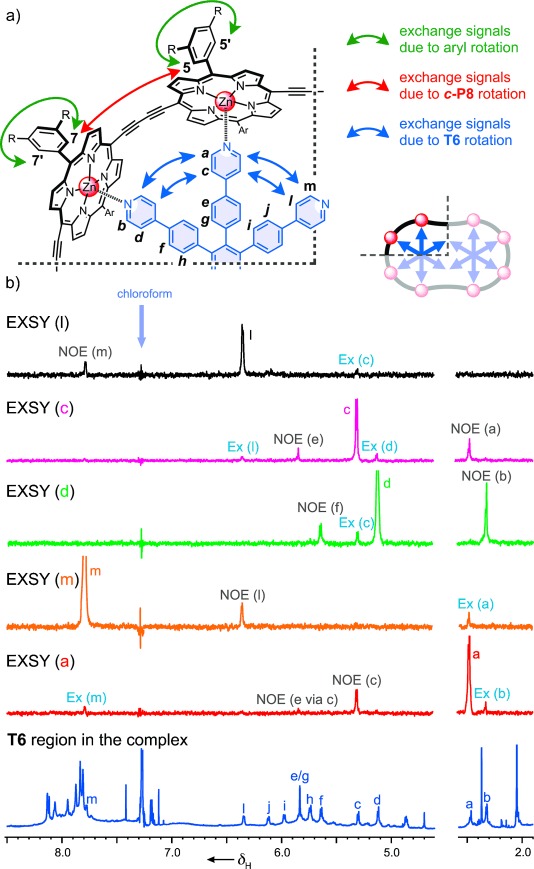
a) Structure of one quarter of the symmetrical *c*-P8⋅(T6)_2_ complex. Arrows indicate the exchange signals detected between various protons in the 1D EXSY NMR experiments. b) (From top to bottom) EXSY NMR spectra (CDCl_3_, 700 MHz, 298 K, 400 ms mixing time) for targeted protons *l* (black), *c* (pink), *d* (green), *m* (orange), *a* (red) and ^1^H NMR spectrum of *c*-P8⋅(T6)_2_ highlighting the region corresponding to the signals for T6.

A second set of 1D EXSY NMR experiments, targeting the porphyrin nanoring protons, was performed to confirm that the nanoring and template rotation in ***c*****-P8⋅**(**T6**)_**2**_ are concerted (i.e. that the motions indicated by the blue and red arrows in Figure 4 proceed at the same rate). The *ortho* proton ***7*** on the porphyrin aryl side-groups and template proton ***d*** were chosen for irradiation in these kinetic experiments (Figure 5 and S43). Spectra were recorded under various mixing times (100–600 ms) to determine the relative rates of exchange. Aryl proton ***7*** generates two types of exchange signals corresponding to 1) the rotation of the aryl group (***7***→***7*** ***′***; rate constant: 4 s^−1^), and 2) the movement of ***c*****-P8** (***7***→***5***; rate constant: 0.13 s^−1^). The rate of growth of the signals corresponding to rotation of the template (***d***→***c***; rate constant: 0.13 s^−1^) is identical, within experimental error, to the rate of rotation of the porphyrin nanoring (***7***→***5***), whereas rotation of the aryl side-groups (***7***→***7*** ***′***) occurs on a faster time-scale (Figure S44). An identical 1D EXSY NMR experiment using ***c*****-P8⋅**(**T4**)_**2**_ confirmed that, in this case, only exchange signals resulting from the aryl side-group rotation are observed (Figure S43). Overall, the results of these experiments provide strong evidence that the three component complex ***c*****-P8⋅**(**T6**)_**2**_ exhibits concerted “caterpillar track” molecular motion.

Similar 1D EXSY NMR experiments on ***c*****-P10⋅**(**T6**)_**2**_ showed that caterpillar track motion occurs faster in this system than in ***c*****-P8⋅**(**T6**)_**2**_, with a rate constant of 10±2 s^−1^ for a 60° rotation, resulting in the observation of exchange signals corresponding to more than one 60° rotation. When the non-coordinated β-pyridyl proton (**a′** in Figure 3) is selectively irradiated, a gradual build-up of exchange signals corresponding to a 60° rotation of the template is observed to **b′**, followed by the appearance of exchange signals corresponding to a 120° rotation to **c′**, and finally by signals corresponding to a 180° rotation to **d′** (Figure S45). The delayed appearance of signals **c′** and **d′** is a clear signature of a sequential step-wise exchange process.[7] This kinetic behavior demonstrates that the template undergoes a step-wise rotation within the nanoring, rather than exchanging through dissociation/association.

Several systems have been reported in which intramolecular rotary motion can be locked by binding a metal cation,[8–11] which suggest that we might be able to control the motion in a molecular caterpillar track by coordinating a metal between the two templates (Figure 6). The distance between the two central nitrogen atoms in the energy-minimized geometry of ***c*****-P10⋅**(**T6**)_**2**_ (Figure 3 b) is 6.8 Å, which is too far to chelate a metal atom, but molecular modeling shows that these pyridine sites can easily move closer together if the complex adopts a helical twist (Figure S50). We tested this idea by carrying out a ^1^H NMR titration (Figure S41). When one equivalent of [PdCl_2_(PhCN)_2_] is added to a solution of ***c*****-P10⋅**(**T6**)_**2**_ in CDCl_3_, new sharp signals appear corresponding to the formation of a complex of stoichiometry ***c*****-P10⋅**(**T6**)_**2**_**⋅PdCl_2_**. The signal from the central α-pyridyl protons shifts from 8.74 ppm in ***c*****-P10⋅**(**T6**)_**2**_ to 8.17 ppm in ***c*****-P10⋅**(**T6**)_**2**_**⋅PdCl_2_**, while other protons are essentially unaffected (Figure S31), confirming that palladium binds between the two central nitrogens. The resonances corresponding to the **T6** template protons in this palladium complex are sharper than those in ***c*****-P10⋅**(**T6**)_**2**_, suggesting that the motion has become locked. 1D EXSY NMR experiments on ***c*****-P10⋅**(**T6**)_**2**_**⋅PdCl_2_** showed no exchange signals between template protons, confirming that palladium effectively prevents the caterpillar track motion in this complex (Figure S46d).

**Figure 6:**
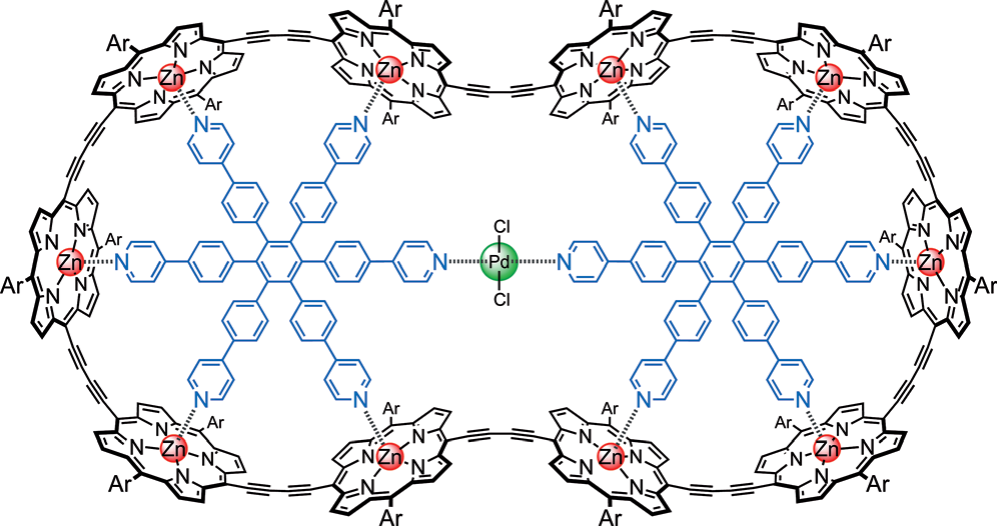
Structure of the *c-*P10⋅(T6)_2_⋅PdCl_2_ complex in which caterpillar track motion is locked.

Caterpillar tracks constitute a new type of supramolecular system exhibiting correlated motion between several movable parts.[12] Previously studied systems include molecular gears,[13] ball bearings,[14] turnstiles,[15] and rotary transduction modules.[16] In a caterpillar track, the motion of the two wheels must be conrotatory, whereas in a gear the motion is disrotatory. The results presented here demonstrate a new approach to the transmission of molecular motion. The formation of caterpillar track complexes also provides useful access to large macrocycles, via template directed synthesis.
