# Supplementary material for: Caterpillar Track Complexes in Template-Directed Synthesis and Correlated Molecular Motion
Source: Angew Chem Int Ed Engl. 2015 Feb 12;54(18):5355–9. doi: 10.1002/anie.201412293 (PMC4471551; doi:10.1002/anie.201412293)
Supplement: Supplementary file 1 [file anie0054-5355-sd1.pdf]

Angewandte Chemie
Eine Zeitschrift der Gesellschaft Deutscher Chemiker

Supporting Information

Caterpillar Track Complexes in Template-Directed Synthesis and Correlated Molecular Motion**

*Shiqi Liu, Dmitry V. Kondratuk, Sophie A. L. Rousseaux, Guzmán Gil-Ramírez, Melanie C. O'Sullivan, Jonathan Cremers, Tim D. W. Claridge, and Harry L. Anderson**

anie_201412293_sm_miscellaneous_information.pdf

Table of Contents

A. General methods **S2**

B. Synthetic procedures **S2**

C. Characterization of nanoring-template complexes (spectra & analysis) **S6**

C1. Characterization of ***c*-P$_{t\text{-Bu}}$8•(T4)$_2$** *S6*

C2. Characterization of ***c*-P$_{t\text{-Bu}}$8•(T6)$_2$** *S10*

C3. Comparison of Δδ for the template protons in the ^{1}H NMR of ***c*-P$_{t\text{-Bu}}$8•(T4)$_2$** *and* ***c*-P$_{t\text{-Bu}}$8•(T6)$_2$** *S11*

C4. Characterization of ***c*-P$_{t\text{-Bu}}$10•(T6)$_2$** *S13*

C5. Characterization of ***c*-P$_{t\text{-Bu}}$10•(T6)$_2$•PdCl$_2$** *S22*

C6. Comparison of Δδ for the template protons in the ^{1}H NMR of ***c*-P$_{t\text{-Bu}}$10•(T4)$_2$** *and* ***c*-P$_{t\text{-Bu}}$10•(T6)$_2$•PdCl$_2$** *S29*

C7. Analytical GPC traces *S30*

D. Binding studies of templates within nanorings **S32**

D1. Binding of **T4** *with* ***c*-P8** *S32*

D2. Binding of **T5** *with* ***c*-P10** *S33*

D3. Binding of **T6** *with* ***c*-P8** *S33*

D4. Binding of **T6** *with* ***c*-P10** *S34*

D5. Binding of **Pd(PhCN)$_2$Cl$_2$** *with* ***c*-P$_{t\text{-Bu}}$10•(T6)$_2$** *S35*

E. Exchange spectroscopy experiments **S36**

E1. EXSY experiments for ***c*-P$_{t\text{-Bu}}$8•(T6)$_2$** *S36*

E2. EXSY experiments for ***c*-P$_{t\text{-Bu}}$10•(T6)$_2$** and ***c*-P$_{t\text{-Bu}}$10•(T6)$_2$•PdCl$_2$** *S38*

F. Small-angle X-ray scattering analysis for *c*-P8•(T4)$_2$ and *c*-P10•(T5)$_2$ **S41**

G. Calculated geometry of *c*-P10•(T6)$_2$•PdCl$_2$ **S43**

H. References **S43**

A. General Methods

All reagents were purchased from commercial sources and solvents were used as supplied unless otherwise noted. Dry solvents ($CHCl_3$, CH_2Cl_2 and toluene) were obtained by passing through alumina under N_2. Diisopropylamine was dried over calcium hydride, distilled and stored under N_2 over molecular sieves. NMR data were recorded at 500 MHz using a Bruker AVII500 (with cryoprobe) or DRX500, or at 400 MHz using a Bruker AVII400 or AVIII400, or at 700 MHz using a Bruker AVIII700 (with cryoprobe) at 298 K. EXchange SpectroscopY NMR experiments were run with an excitation sculpting single-echo selection scheme, using a 80 ms, 180° Gaussian shaped pulse. For each measurement, 64 or 256 scans were taken, with 2 s recovery delay. A single 180° hard pulse was applied at the mid-point of the mixing time to minimize recovery of unwanted signals during the mixing time. Chemical shifts are quoted as parts per million (ppm) relative to residual $CHCl_3$ (δ_H 7.27 ppm for ^{1}H NMR and at δ_C 77.2 ppm for ^{13}C NMR) and coupling constants (*J*) are reported in Hertz. MALDI-ToF spectra were measured at the EPSRC National Mass Spectrometry service (Swansea) using the Applied Biosystems Voyager DE-STR. UV/vis/NIR absorbance measurements were recorded at 25 °C with a Perkin-Elmer Lambda 20 photospectrometer using quartz 1 cm cuvettes. UV/vis/NIR titrations were analyzed by calculating the difference in absorptions and plotted using Origin™ software or fitted with predicted models using SPECFIT™ software. Size exclusion chromatography (SEC) was carried out using Bio-Beads S-X1, 200–400 mesh (Bio Rad). Analytical and semi-preparative GPC were carried out on Shimadzu Recycling GPC system equipped with LC-20 AD pump, SPD-M20A UV detector and a set of JAIGEL 3H (20 × 600 mm) and JAIGEL 4H (20 × 600 mm) columns in toluene/1% pyridine as eluent with a flow rate of 3.5 mL/min.

B. Synthetic procedures

Porphyrin dimer ***l*-P2**,[S1] template **T6**,[S2] template **T5**,[S3] template **T4**[S3] and pentaphenylbenzene[S4] were prepared using previously reported procedures.

The scaled-up synthesis of ***c*-P$_{C8}$10** and ***c*-P$_{t\text{-}Bu}$10** was performed using the more synthetically accessible pentadentate template **T5*** whose synthesis is presented below.

Penta-(*p*-iodophenyl)benzene

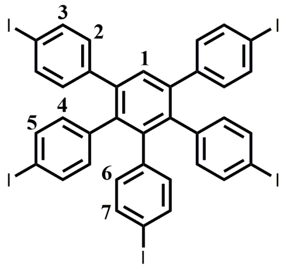

Pentaphenylbenzene[S3] (2.24 g, 4.88 mmol), bis(trifluoroacetoxy)iodobenzene (7.08 g, 16.46 mmol) and iodine (3.88 g, 15.29 mmol) were dissolved in dry CH_2Cl_2 (180 mL). The reaction mixture was stirred in the dark for 17 hours. The product was precipitated by the addition of hexane (400 mL). The precipitate was filtered and washed with hexane. The product was then dried under high vacuum to yield the title compound (3.99 g, 75%) as a light pink solid.

^{1}H NMR (400 MHz, CD_2Cl_2): δ_H(ppm) 7.57 (d, *J* = 8.4 Hz, 4H, H3), 7.46 (s, 1H, H1), 7.38 (d, *J* = 8.4 Hz, 4H, H5), 7.32 (d, *J* = 8.4 Hz, 2H, H7), 6.90 (d, *J* = 8.5 Hz, 4H, H2), 6.62 (d, *J* = 8.5 Hz, 4H, H4), 6.56 (d, *J* = 8.5 Hz, 2H, H6).

^{13}C NMR (125 MHz, CD_2Cl_2): δ_C(ppm) 141.0, 141.0, 140.5, 139.6, 139.2, 138.6, 138.0, 136.9, 136.6, 133.6, 133.5, 132.1, 131.8, 92.9, 92.4, 92.2.

Pentadentate Template T5*

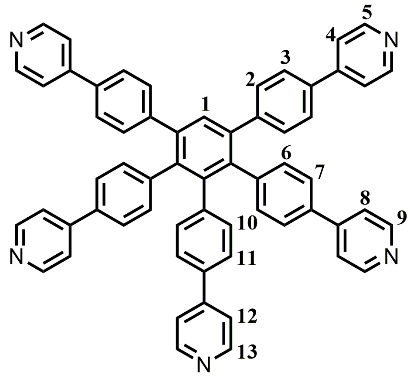

To a solution of penta-(*p*-iodophenyl)benzene (0.50 g, 0.46 mmol) in 1,2-dimethoxyethane (15 mL) and THF (20 mL) was added dichlorobis(triphenylphosphine)palladium(II) (65 mg, 0.09 mmol). After addition of water (20 mL), $NaHCO_3$ (0.70 g, 8.25 mmol) and 4-pyridineboronic acid (1.35 g, 11.05 mmol), the mixture was deoxygenated by freeze-pump-thawing (3 cycles), backfilled with argon and stirred at 70 °C for 5 days. The solvents were removed and the crude product was purified by flash column chromatography on silica gel (CH_2Cl_2 : methanol : triethylamine; gradient 100 : 1 : 0.25 to 100 : 5 : 0.5) to give the template as a white solid. The solid product was washed with water and dried under high vacuum (80 mg, 21%).

^{1}H NMR (400 MHz, $CDCl_3$): δ_H(ppm) 8.62 (d, J = 5.8 Hz, 4H, H5), 8.56 (d, J = 5.8 Hz, 4H, H9), 8.54 (d, J = 6.0 Hz, 2H, H13), 7.70 (s, 1H, H1), 7.52 (d, J = 8.4 Hz, 4H, H4), 7.47 (d, J = 6.1 Hz, 4H, H8), 7.39 (d, J = 6.0 Hz, 4H, H3), 7.35–7.32 (m, 10H, H12 + H7 + H2), 7.26 (d, J = 8.4 Hz, 2H, H11), 7.05 (d, J = 8.2 Hz, 4H, H6), 6.98 (d, J = 8.4 Hz, 2H, H10).

^{13}C NMR (125 MHz, $CDCl_3$): δ_C(ppm) 150.0, 149.9, 149.8, 147.8, 147.7, 142.1, 141.2, 141.0, 140.6, 140.6, 138.9, 136.2, 135.3, 135.1, 132.2, 132.2, 131.9, 130.6, 126.5, 125.8, 125.6, 121.4, 121.2, 121.2 (one signal missing, presumably due to overlap).

***m/z* (ESI)** 844.34 ($C_{61}H_{41}N_5$, $M+H^+$ requires 844.34).

***tert*-Butyl Cyclic Porphyrin Octamer *c*-P$_{t\text{-Bu}}$8**

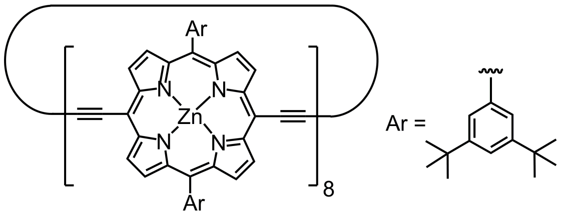

Tetradentate template **T4** (3.96 mg, 4.70 μmol) and deprotected porphyrin dimer ***l*-P$_{t\text{-Bu}}$2** (14.8 mg, 9.29 μmol) were dissolved in $CHCl_3$ (19 mL) and sonicated for 10 minutes. A catalyst solution was prepared by dissolving dichlorobis(triphenylphosphine)palladium(II) (3.29 mg, 4.69 μmol), CuI (4.47 mg, 23.5 μmol) and 1,4-benzoquinone (10.2 mg, 94.4 μmol) in $CHCl_3$ (2 mL) and freshly distilled diisopropylamine (0.03 mL). The catalyst solution was added to the template and porphyrin dimer mixture. The reaction mixture was stirred vigorously at room temperature overnight under air. A second batch of catalyst was added and the mixture was stirred at 40 °C for 2 h. The reaction progress was monitored by UV-vis spectroscopy. Once the reaction was judged to have reached completion, the reaction mixture was passed through a plug of alumina using $CHCl_3$ as the eluent, and purified by size

exclusion chromatography on Bio-Beads S-X1 using toluene/10% pyridine. The desired product was purified from the crude mixture by recycling GPC (toluene/1% pyridine). The separation gave 4.26 mg (29% yield) of ***c*-P$_{t\text{-Bu}}$8** as a dark brown solid.

^{1}H NMR (400 MHz, $CDCl_3$ + 1% pyridine-d_5, 298 K): δ_H(ppm) 9.70 (d, J = 4.6 Hz, 32H, -ArH_β), 8.83 (d, J = 4.6 Hz, 32H, -ArH_β), 7.93 (d, J = 1.7 Hz, 32H, -ArH_{ortho}), 7.72 (t, J = 1.8 Hz, 16H, -ArH_{para}), 1.46 (s, 288H, -*t*Bu*H*).

***m/z* (MALDI-ToF)** 6367 ($C_{416}H_{400}N_{32}Zn_8$, M^+ requires 6371).

λ_{max} ($CHCl_3$ / 1% pyridine) / nm (log ε) 466 (5.92), 492 (5.94), 817 (5.72).

Octyloxy Cyclic Porphyrin Octamer *c*-P$_{C8}$8

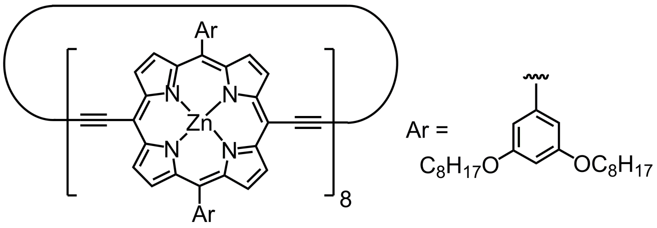

Tetradentate template **T4** (4.0 mg, 4.7 μmol) and deprotected porphyrin dimer ***l*-P$_{C8}$2** (20 mg, 9.4 μmol, 2.0 equiv) were dissolved in $CHCl_3$ (19 mL) and sonicated for 10 minutes. A catalyst solution was prepared by dissolving dichlorobis(triphenylphosphine)palladium(II) (3.3 mg, 4.7 μmol), CuI (4.5 mg, 24 μmol) and 1,4-benzoquinone (10 mg, 94 μmol) in $CHCl_3$ (2 mL) and freshly distilled diisopropylamine (0.03 mL). The catalyst solution was added to the template and porphyrin dimer mixture. The reaction mixture was stirred vigorously at room temperature overnight under air, then passed through a plug of alumina using $CHCl_3$ as the eluent, and purified by size exclusion chromatography on Bio-Beads S-X1 using toluene/10% pyridine. The desired product was purified from the crude mixture by recycling GPC (toluene/1% pyridine). The separation gave 2.8 mg (14% yield) of ***c*-P$_{C8}$8** as a dark brown solid.

^{1}H NMR (400 MHz, $CDCl_3$ + 1% pyridine-d_5, 298 K): δ_H(ppm) 9.72 (d, J = 4.5 Hz, 32H, -ArH_β), 8.95 (d, J = 4.5 Hz, 32H, -ArH_β), 7.32 (s, 32H, -ArH_{ortho}), 6.88 (s, 16H, -ArH_{para}), 4.14 (t, J = 6.5 Hz, 64 H, -OCH_2), 1.89–1.84 (m, 64H, -CH_2), 1.55–1.26 (m, 320 H, -CH_2), 0.83 (t, J = 6.8 Hz, 96 H, -CH_3).[S5]

***tert*-Butyl Cyclic Porphyrin Decamer *c*-P$_{t\text{-Bu}}$10**

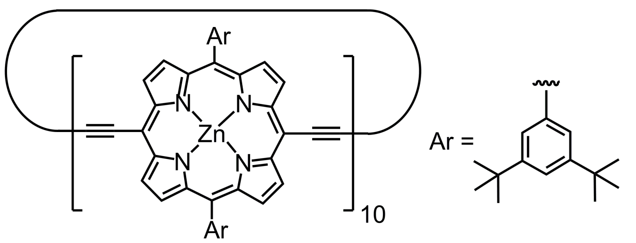

Pentadentate template **T5*** (5.0 mg, 5.9 μmol) and deprotected porphyrin dimer ***l*-P$_{t\text{-Bu}}$2** (22.0 mg, 13.8 μmol) were dissolved in $CHCl_3$ (22 mL), sonicated for 15 min and then cooled to 20 °C. A catalyst solution was prepared by dissolving dichlorobis(triphenylphosphine)-palladium(II) (1.0 mg, 1.42 μmol), CuI (1.0 mg, 5.3 μmol) and 1,4-benzoquinone (3.0 mg, 27.7 μmol) in $CHCl_3$ (0.7 mL) and freshly distilled diisopropylamine (35 μL). The catalyst solution was added to the template and porphyrin dimer mixture, which was then stirred at 20 °C overnight. This was followed by the addition of a 6-fold amount of the initial catalyst loading and further stirring for 3 hours. The reaction mixture was passed over a short alumina plug ($CHCl_3$) followed by a size exclusion column on Biobeads SX-1 (toluene/10% pyridine)

and further purified by recycling GPC (toluene/1% pyridine) to give 4.0 mg (18%) of ***c*-P$_{t\text{-Bu}}$10** as brown solid.

^{1}H NMR (400 MHz, $CDCl_3$, 298 K): δ_H(ppm) 9.82 (d, J = 4.6 Hz, 40H, -ArH_β), 8.94 (d, J = 4.6 Hz, 40H, -ArH_β), 8.03 (d, J = 1.7 Hz, 40H, -ArH_{ortho}), 7.80 (t, J = 1.6 Hz, 20H, -ArH_{para}), 1.54 (s, 360H, -*t*Bu*H*).

***m/z* (MALDI-TOF)** 7955 ($C_{520}H_{500}N_{40}Zn_{10}$, M^+ requires 7963).

λ_{max} ($CHCl_3$ / 1% pyridine) / nm (log ε) 468 (5.89), 596 (4.76), 822 (5.71).

Octyloxy Cyclic Porphyrin Decamer *c*-P$_{C8}$10

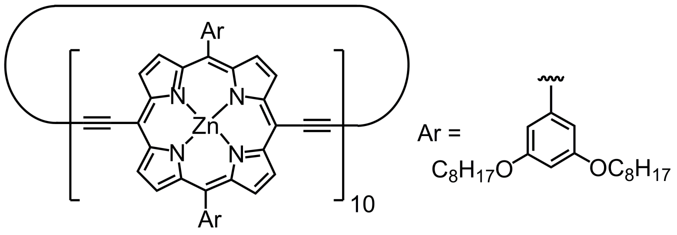

Method 1

Pentadentate template **T5*** (10.1 mg, 11.9 µmol) and deprotected porphyrin dimer ***l*-P$_{C8}$2** (65.0 mg, 29.9 µmol) were dissolved in $CHCl_3$ (65 mL), sonicated for 15 min and then cooled to 20 °C. A catalyst solution was prepared by dissolving dichlorobis(triphenylphosphine)-palladium(II) (2.2 mg, 3.0 µmol), CuI (2.2 mg, 11.8 µmol) and 1,4-benzoquinone (6.7 mg, 62 µmol) in $CHCl_3$ (1.6 mL) and freshly distilled diisopropylamine (80 µL). The catalyst solution was added to the template and porphyrin dimer mixture, which was then stirred at 20 °C overnight. This was followed by the addition of a 5-fold amount of the initial catalyst loading and further stirring for 3 h. The reaction mixture was passed over a size exclusion column on Biobeads SX-1 ($CHCl_3$/20% pyridine) and recycling GPC (toluene/1% pyridine) to give 11.4 mg (18%) of ***c*-P$_{C8}$10** as brown solid.

^{1}H NMR (500 MHz, $CDCl_3$, 298 K): δ_H(ppm) 9.74 (d, 40H, J = 4.5 Hz, -ArH_β), 8.96 (d, 40H, J = 4.5 Hz, -ArH_β), 7.29 (m, 40H, -ArH_{ortho}), 6.84 (m, 20H, -ArH_{para}), 4.07 (s br, 80H, -OCH_2), 1.83–1.77 (m, 80H, CH_2), 1.46–1.40 (m, 80H, CH_2), 1.32–1.16 (m, 320H, CH_2), 0.82–0.74 (m, 120H, -CH_3).

***m/z* (MALDI-TOF)** 10852 ($C_{680}H_{820}N_{40}O_{40}Zn_{10}$, M^+ requires 10848).

λ_{max} (toluene / 1% pyridine) / nm (log ε) 471 (6.08), 491 (6.06), 806 (5.87).

Method 2

Pentadentate template **T5** (7.7 mg, 8.4 µmol) and deprotected porphyrin dimer ***l*-P$_{C8}$2** (45.6 mg, 0.021 mmol) were dissolved in $CHCl_3$ (45 mL), sonicated for 30 min and then cooled to 20 °C. A catalyst solution was prepared by dissolving dichlorobis(triphenyl-phosphine)palladium(II) (3.0 mg, 4.2 µmol), CuI (4.0 mg, 2.5 µmol) and 1,4-benzoquinone (9.0 mg, 84 µmol) in $CHCl_3$ (2.1 mL) and freshly distilled diisopropylamine (100 µL). The catalyst solution was added to the template and porphyrin dimer mixture, which was then stirred at 20 °C overnight. The reaction mixture was passed over a size exclusion column on Biobeads SX-1 ($CHCl_3$/10% pyridine) and recycling GPC (toluene/1% pyridine) to give 9.0 mg (20 %) of ***c*-P$_{C8}$10** as brown solid.

C. Characterization of Nanoring-Template Complexes

C1) Characterization of ***c*-P$_{t\text{-Bu}}$8•(T4)$_2$**

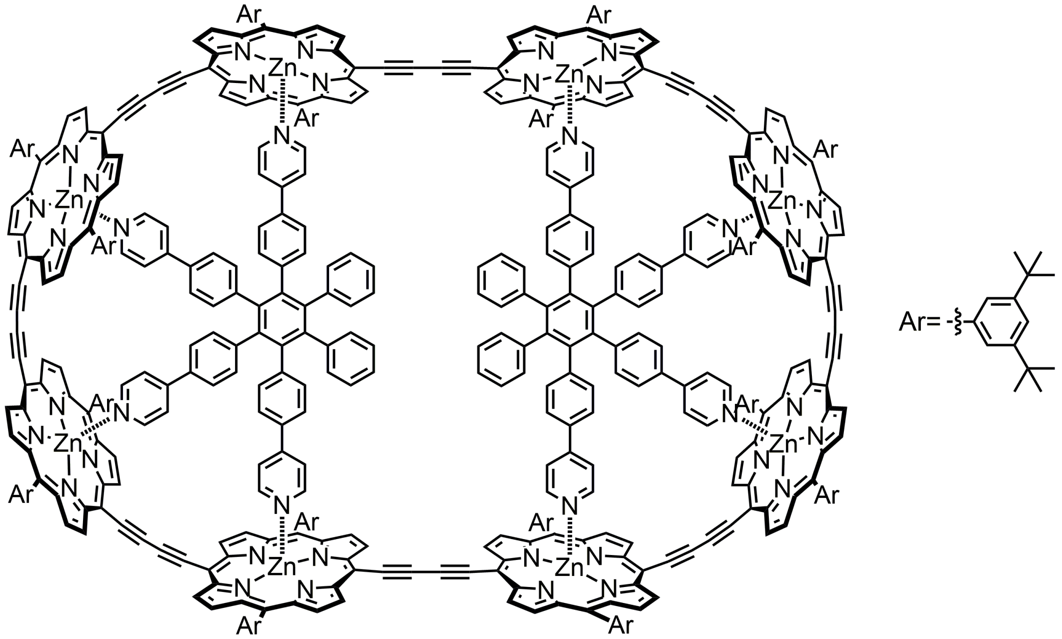

^{1}H NMR (400 MHz, $CDCl_3$, 298 K): δ_H (ppm) 9.71 (d, J = 4.2 Hz, 8H) 9.64–9.63 (m, 24H), 8.88–8.84 (m, 32H), 8.12 (br s, 8H), 8.04 (br s, 8H), 7.96 (br s, 8H), 7.90 (br s, 8H), 7.84 (br s, 8H), 7.82 (br s, 8H), 5.92–5.89 (m, 8H), 5.85–5.80 (m, 20H), 5.75–5.71 (m, 16H), 5.61 (d, J = 8.5 Hz, 8H), 5.26–5.24 (m, 8H), 5.12–5.11 (m, 8H), 2.49–2.47 (m, 8H), 2.37–2.35 (m, 8H), 1.62–1.56 (m, 288H).

^{13}C NMR (125 MHz, $CDCl_3$): δ_C (ppm) 152.9, 152.0, 151.8, 151.7, 150.6, 150.5, 150.4, 150.4, 149.3, 149.3, 148.7, 148.6, 147.2, 146.8, 143.4, 143.3, 141.7, 141.6, 141.1, 140.8, 140.6, 139.4, 139.1, 138.4, 133.8, 133.3, 133.2, 132.4, 131.2, 131.1, 130.7, 130.6, 129.9, 126.2, 125.7, 125.4, 124.3, 124.2, 121.3, 121.3, 119.6, 119.5, 100.5, 100.5, 100.4, 100.3, 97.2, 96.8, 95.3, 90.3, 90.1, 87.8, 83.3, 35.4, 35.4, 35.3, 32.2, 32.2, 32.1, 30.1 (seven signals missing, presumably due to overlap).

m/z (MALDI-TOF) 6364 ($C_{540}H_{484}N_{40}Zn_8$, M^+ – 2 **T4** requires 6372).

λ_{max} ($CHCl_3$) / nm (log ε) 442 (5.70), 498 (5.93), 786 (5.46), 811 (5.63), 859 (5.93).

Based on the symmetry in the ***c*-P$_{t\text{-Bu}}$8•(T4)$_2$** complex, only one quarter of the molecule needs to be considered in the interpretation of the ^{1}H NMR spectrum (Figure S1). The chemical structure in Figure S1 represents the two porphyrin units contained in this quarter. The portion of the aryl side group that points away from the template is drawn in bold and the portion of the template that coordinates the porphyrin units is also drawn. The full assignment of the ^{1}H NMR spectrum for the ***c*-P$_{t\text{-Bu}}$8•(T4)$_2$** complex is outlined in the following section. This was completed by comparison with similar templated-ring systems[S6] as well as with the assistance of COSY and ROESY NMR experiments.

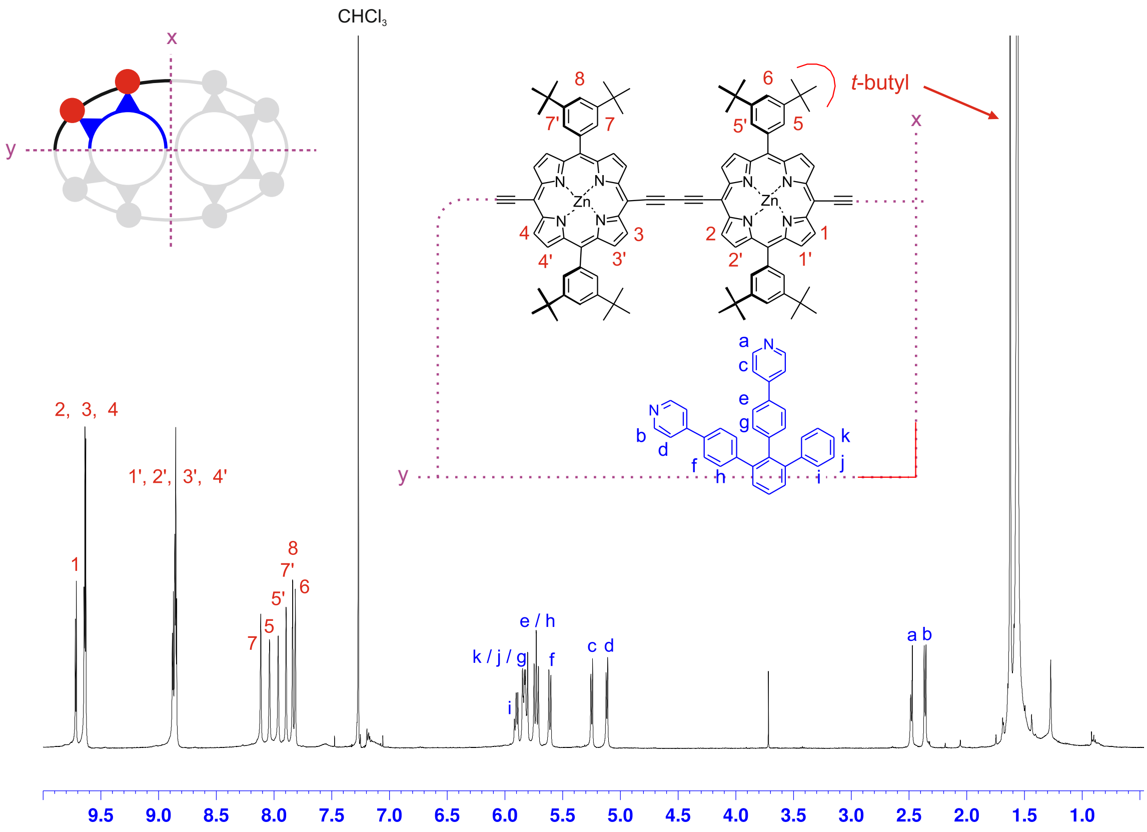

Figure S1. ^{1}H NMR spectrum of ***c*-P$_{t\text{-Bu}}$8•(T4)$_2$** (500 MHz, $CDCl_3$, 298 K).

The proton assignments for ***c*-P$_{t\text{-Bu}}$8•(T4)$_2$** are based on the initial assumption that the most distinct porphyrin β-proton (with the highest chemical shift) at δ_H = 9.71 ppm, corresponds to proton ***1***, which connects the relatively straight acetylene bridge in ***c*-P$_{t\text{-Bu}}$8•(T4)$_2$**. This assumption was later confirmed by EXSY NMR experiments.

Due to ring current effects, the α-protons ***a*** and ***b*** on the template pyridine moieties are strongly shielded and appear at very low chemical shifts (between 2.30-2.50 ppm). In the ROESY spectrum, the porphyrin β-proton ***1*** shows an NOE with the template α-proton ***a***. Thus, the template α-proton ***b*** must be bound to the other porphyrin unit (Figure S2).

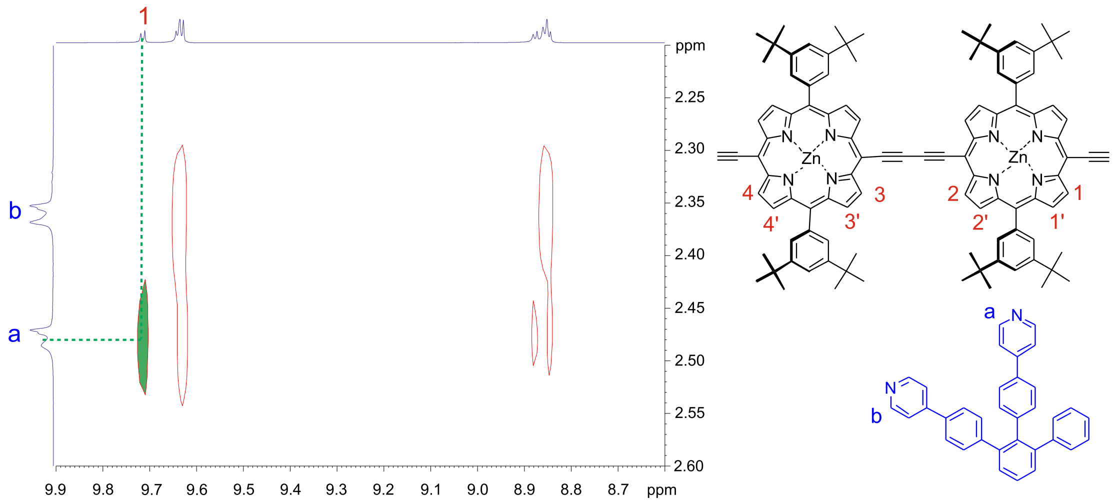

Figure S2. Region of the ROESY spectrum of ***c*-P$_{t\text{-Bu}}$8•(T4)$_2$** corresponding to the porphyrin β-protons and the **T4** pyridine α-protons (500 MHz, $CDCl_3$, 298 K, 500 ms mixing time).

The template β-protons ***c*** and ***d*** are less affected by the ring current and are therefore less shielded than α-protons ***a*** and ***b***. Based on their coupling with protons ***a*** and ***b*** in the COSY spectrum, their assignment can be confirmed (Figure S3).

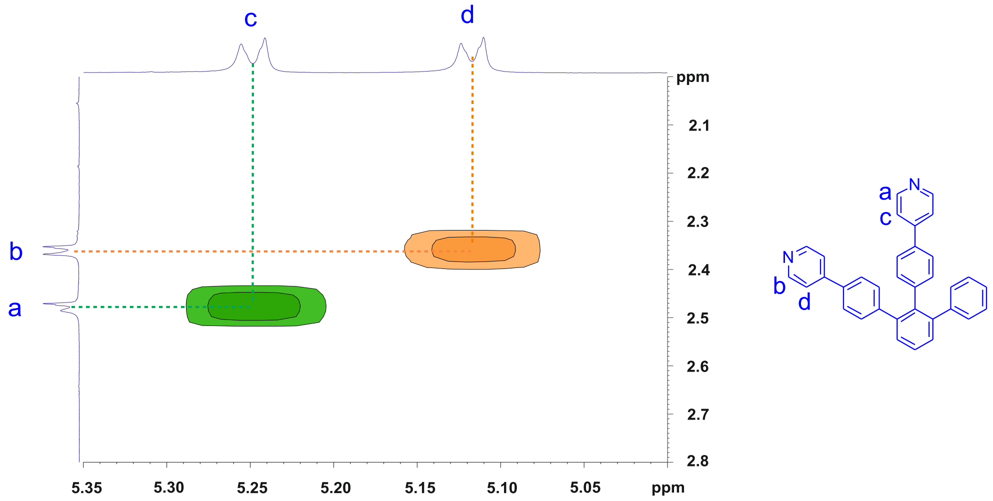

Figure S3. Region of the COSY spectrum of ***c*-P$_{t\text{-Bu}}$8•(T4)$_2$** corresponding to protons ***a*** and ***b*** and protons ***c*** and ***d*** on **T4** (500 MHz, $CDCl_3$, 298 K).

In the ROESY spectrum, template protons ***c*** and ***d*** show NOEs with protons ***e*** and ***f***, respectively. In addition, a weaker correlation is observed between protons ***d*** and ***h***, possibly arising from a longer-range effect (Figure S4). This helps clarify the assignment for protons ***g*** and ***h*** that couple to protons ***e*** and ***f***, respectively, in the COSY spectrum (Figure S5).

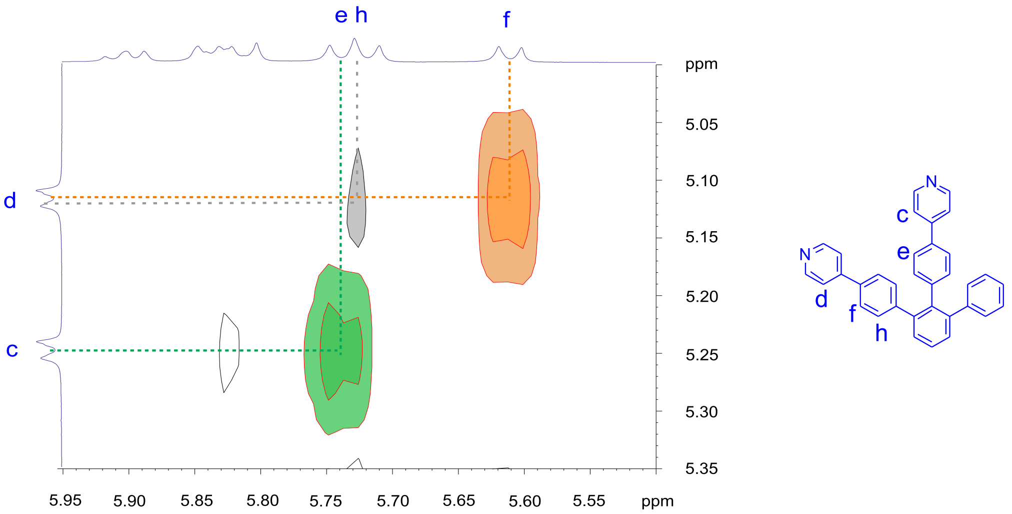

Figure S4. Region of the ROESY spectrum of ***c*-P$_{t\text{-Bu}}$8•(T4)$_2$** corresponding to template protons ***c*** and ***d*** and protons ***e***, ***f*** and ***h*** (500 MHz, $CDCl_3$, 298 K, 500 ms mixing time).

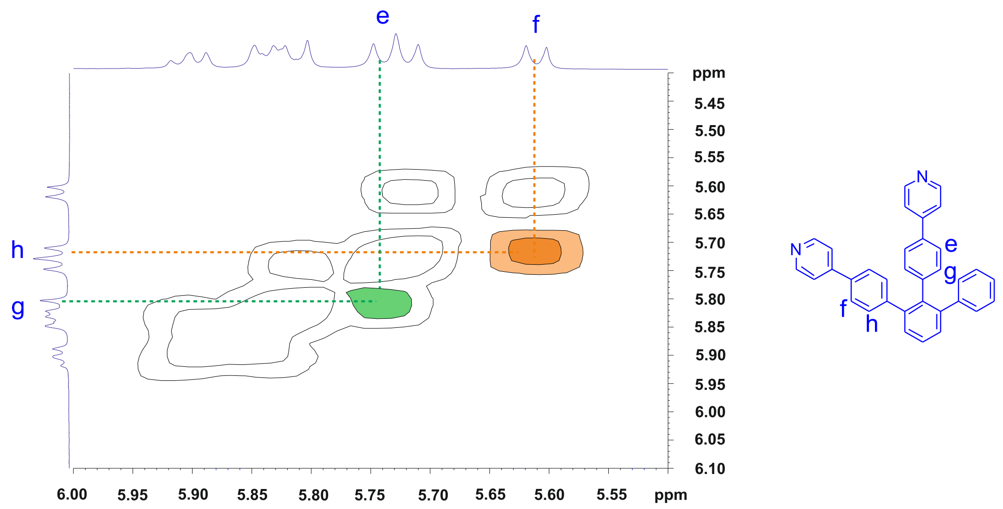

Figure S5. Region of the COSY spectrum of ***c*-P$_{t\text{-Bu}}$8•(T4)$_2$** corresponding to protons ***e*** and ***f*** and protons ***g*** and ***h*** (500 MHz, $CDCl_3$, 298 K).

The assignment of protons ***g*** and ***h*** is further confirmed by their NOEs to protons ***e*** and ***f***, respectively, in the ROESY spectrum (Figure S6). Also, in the ROESY spectrum, proton ***g*** shows an NOE to proton ***i***. Since the multiplet in the region corresponding to proton ***g*** integrates for five protons, this multiplet must also correspond to protons ***j*** and ***k***. This is further confirmed by the NOE of ***j*/*k*** with ***e*** in the ROESY spectrum.

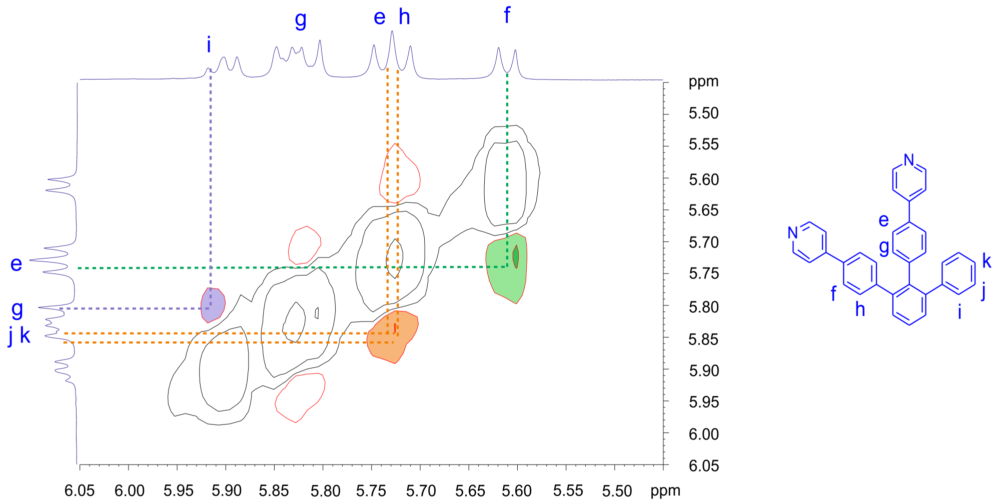

Figure S6. Region of the ROESY spectrum of ***c*-P$_{t\text{-Bu}}$8•(T4)$_2$** which confirms the assignments of template protons ***e***, ***g***, ***i***, ***j*** and ***k*** (500 MHz, $CDCl_3$, 298 K, 500 ms mixing time).

The protons of the aryl solubilizing side groups can also be assigned based on their correlations with protons ***a*** and ***b*** in the ROESY spectrum. As shown in Figure S7, protons ***5*** and ***5'*** appear to be on the same aryl group, and due to their strong correlation signals, protons ***5*** and ***7*** are pointing to the inside of the ring (towards the templates).

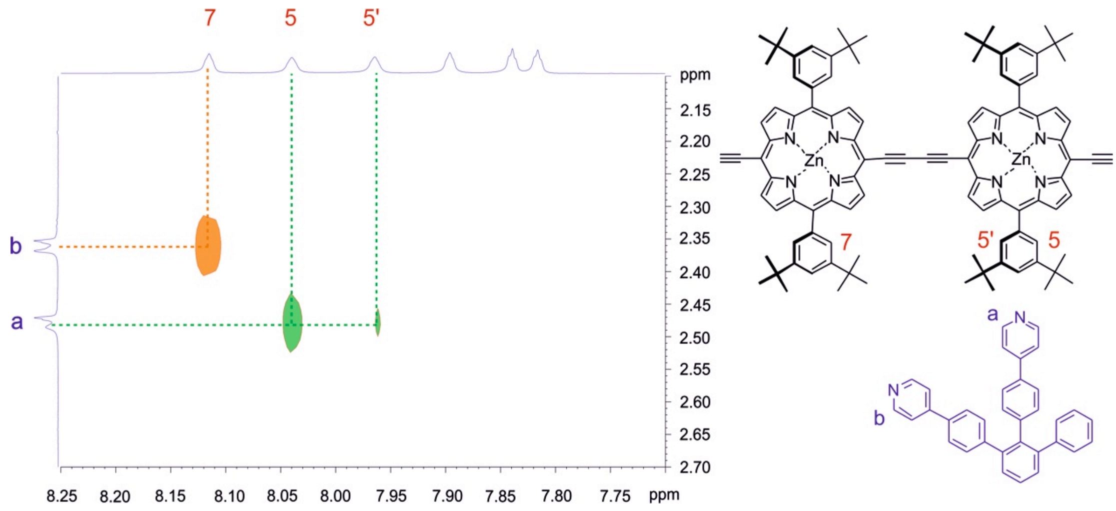

Figure S7. Region of the ROESY spectrum of ***c*-P$_{t\text{-Bu}}$8•(T4)$_2$** corresponding to the β-protons on the template and the aryl protons on the porphyrin units (500 MHz, $CDCl_3$, 298 K, 500 ms mixing time).

The remaining protons of the aryl side groups can be assigned based on their correlations in the COSY spectrum (Figure S8).

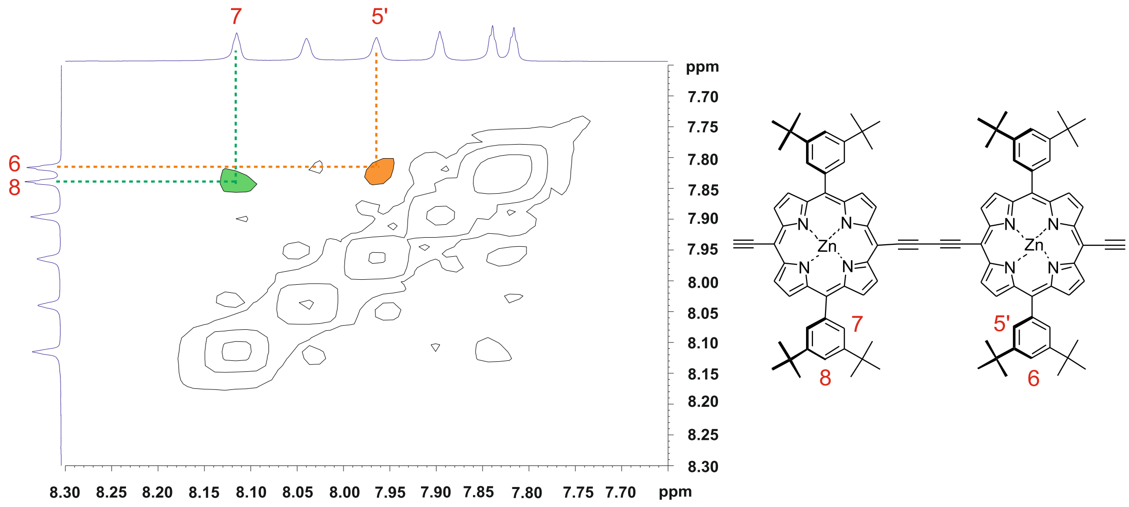

Figure S8. Region of the COSY spectrum of ***c*-P$_{t\text{-Bu}}$8•(T4)$_2$** corresponding to the aryl protons on the side groups of the porphyrin units for the assignment of protons ***6*** and ***8*** (500 MHz, $CDCl_3$, 298 K).

C2) Characterization of ***c*-P$_{t\text{-Bu}}$8•(T6)$_2$**

The assignment of the relevant protons for the caterpillar track complex ***c*-P$_{t\text{-Bu}}$8•(T6)$_2$** was carried out by comparison with the parent stationary complex ***c*-P$_{t\text{-Bu}}$8•(T4)$_2$** and with the assistance of 1D EXSY NMR experiments (Figure S9).

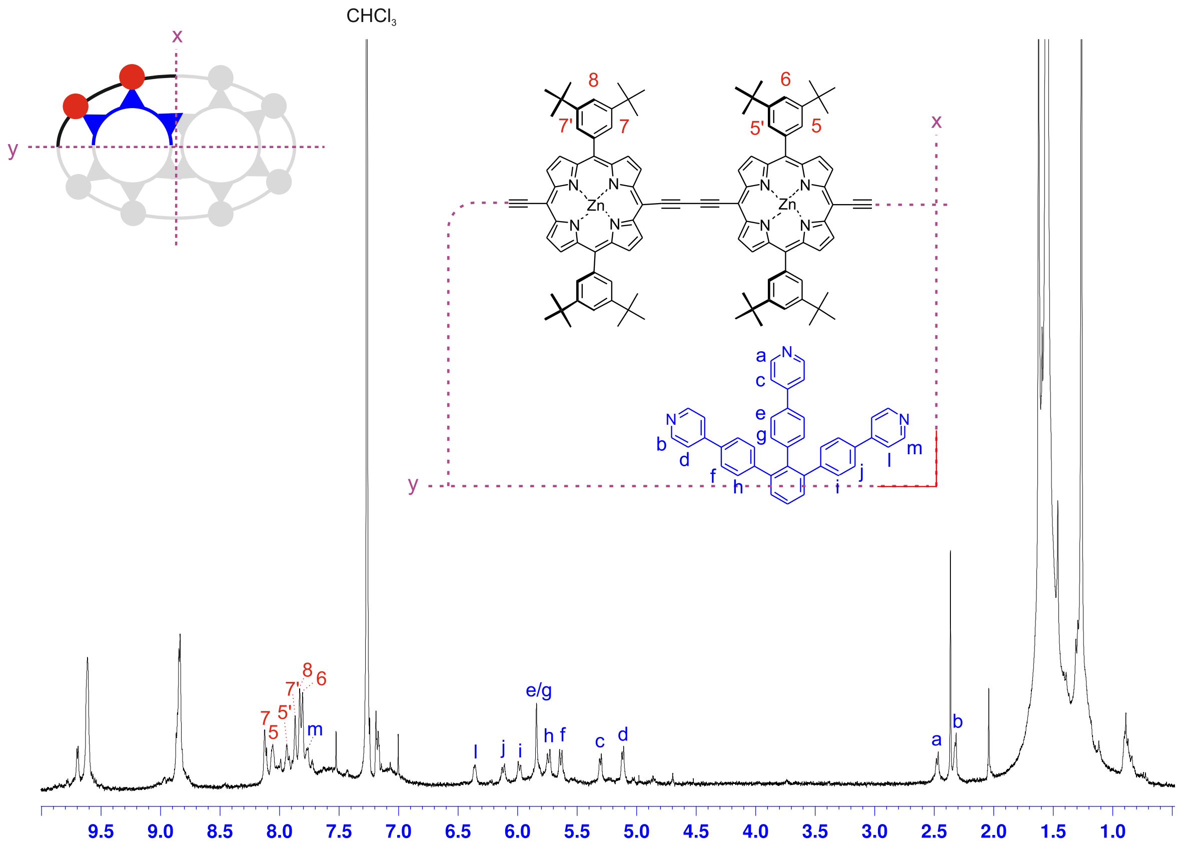

Figure S9. ^{1}H NMR spectrum of ***c*-P$_{t\text{-Bu}}$8•(T6)$_2$** (700 MHz, $CDCl_3$, 298 K).

*C3) Comparison of Δδ for the template protons in the ^{1}H NMR of **c-P$_{t\text{-Bu}}$8•(T4)$_2$** and **c-P$_{t\text{-Bu}}$8•(T6)$_2$***

To further understand the similarities between the ***c*-P$_{t\text{-Bu}}$8•(T4)$_2$** and ***c*-P$_{t\text{-Bu}}$8•(T6)$_2$** complexes, an analysis of the Δδ in the ^{1}H NMR spectra between free templates and bound templates was carried out (Figure S10 and S11, Table S1 and S2). The Δδ are highly similar, despite the different chemical shifts for **T4** compared to **T6** and ***c*-P$_{t\text{-Bu}}$8•(T4)$_2$** compared to ***c*-P$_{t\text{-Bu}}$8•(T6)$_2$** (Figure S12). This provides additional evidence that the binding relationships of **T4** and **T6** with ***c*-P$_{t\text{-Bu}}$8** strongly resemble each other in these two complexes.

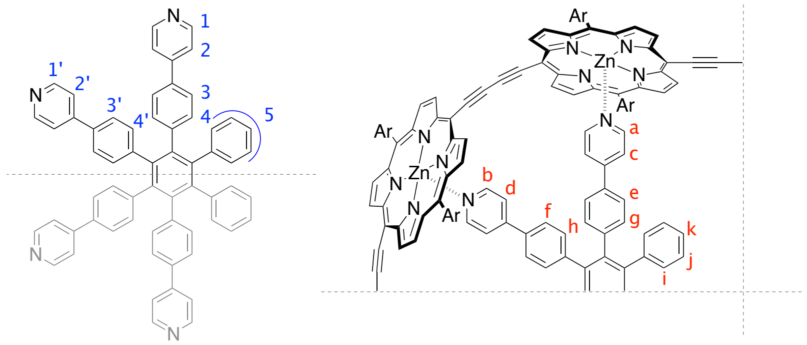

Figure S10. Proton labels for **T4** and complex-bound **T4** in ***c*-P$_{t\text{-Bu}}$8•(T4)$_2$**.

Table S1. ^{1}H NMR chemical shifts for protons on free **T4** and the corresponding protons on bound **T4** in ***c*-P$_{t\text{-Bu}}$8•(T4)$_2$** ($CDCl_3$, 298 K).

**T4**	δ_H (ppm)	***c*-P$_{t\text{-Bu}}$8•(T4)$_2$**	δ_H (ppm)	***Δδ*$_H$ (ppm)**
**1**	8.54	**a**	2.48	**6.06**
**1'**	8.54	**b**	2.36	**6.18**
**2**	7.34	**c**	5.25	**2.09**
**2'**	7.36	**d**	5.18	**2.18**
**3**	7.23	**e**	5.74	**1.49**
**3'**	7.25	**f**	5.61	**1.64**
**4**	7.00	**g**	5.81	**1.19**
**4'**	7.03	**h**	5.72	**1.31**
**5**	6.88	**i**	5.90	**0.98**
		**j, k**	5.84	**1.04**

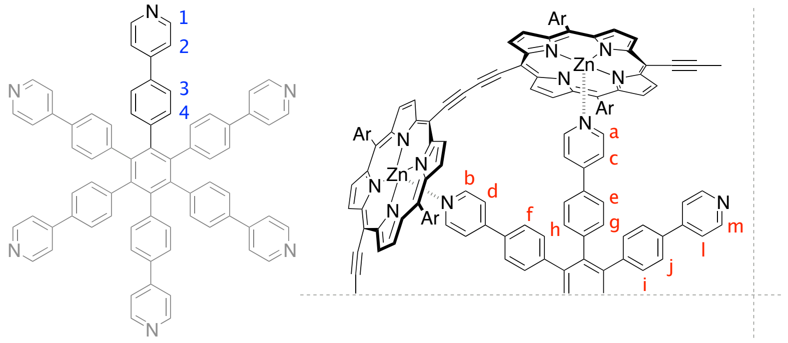

Figure S11. Proton labels for **T6** and complex-bound **T6** in ***c*-P$_{t\text{-Bu}}$8•(T6)$_2$**.

Table S2. ^{1}H NMR chemical shifts for protons on free **T6** and the corresponding protons on bound **T6** in ***c*-P$_{t\text{-Bu}}$8•(T6)$_2$** ($CDCl_3$, 298 K).

**T6**	δ_H (ppm)	***c*-P$_{t\text{-Bu}}$8•(T6)$_2$**	δ_H (ppm)	**Δδ$_H$ (ppm)**
**1**	8.54	**a**	2.47	**6.07**
		**b**	2.32	**6.22**
		**m**	7.77	**0.77**
**2**	7.34	**c**	5.30	**2.03**
		**d**	5.12	**2.21**
		**l**	6.34	**1.00**
**3**	7.23	**e**	5.84	**1.42**
		**f**	5.64	**1.62**
		**j**	6.12	**1.11**
**4**	7.00	**g**	5.74	**1.19**
		**h**	5.84	**1.29**
		**i**	5.98	**1.02**

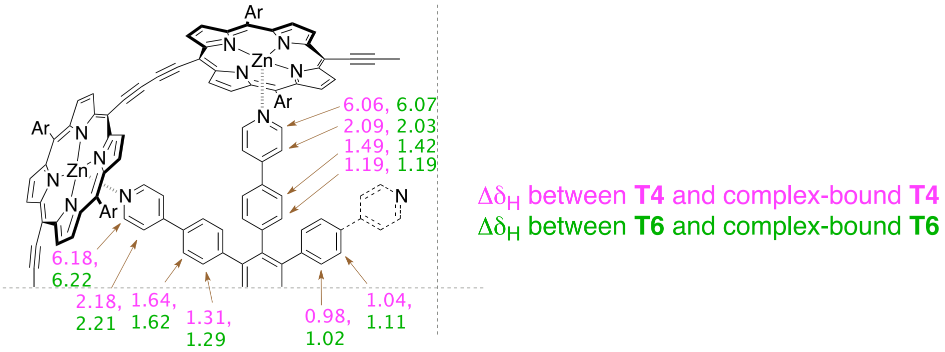

Figure S12. Complexation-induced changes in the chemical shift, *Δδ* in the ^{1}H NMR of free templates and bound templates (pink for **T4** and green for **T6**) in ***c*-P$_{t\text{-Bu}}$8•(T4)$_2$** and ***c*-P$_{t\text{-Bu}}$8•(T6)$_2$** ($CDCl_3$, at 298 K).

C4) Characterization of ***c*-P$_{t\text{-Bu}}$10•(T6)$_2$**

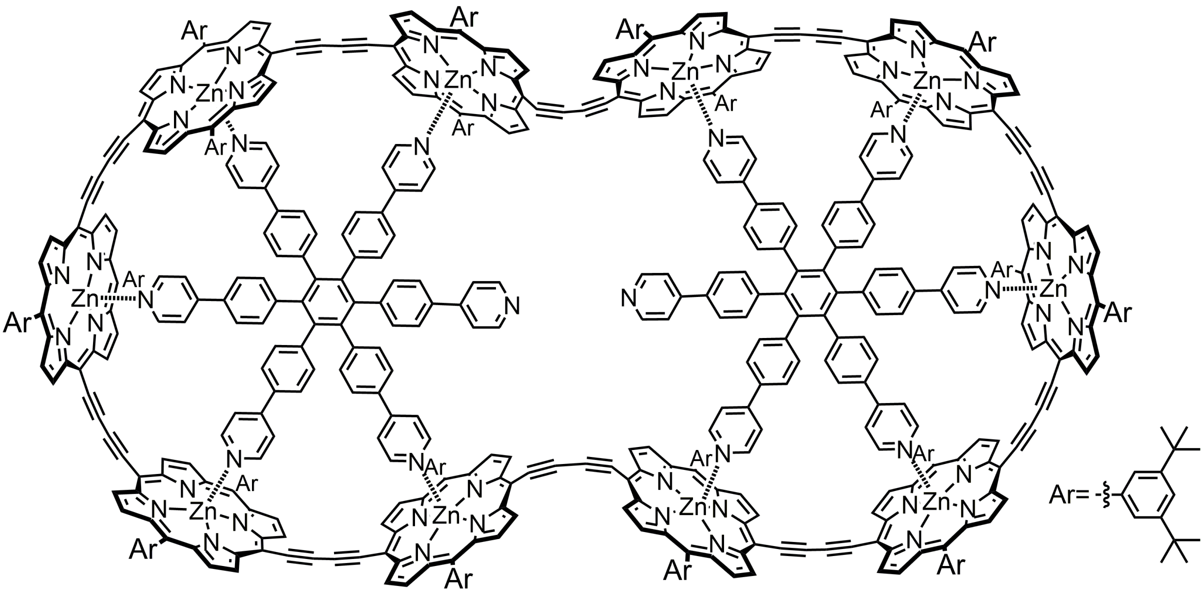

^{1}H NMR (700 MHz, $CDCl_3$, 298 K): δ_H (ppm) 9.66–9.57 (m, 40H), 8.85–8.77 (m, 40H), 8.76–8.71 (m, 4H), 8.11 (br s, 8H), 8.06 (br s, 4H), 7.96 (br s, 8H), 7.92 (br s, 8H), 7.89 (br s, 4H), 7.87 (br s, 8H), 7.83 (br s, 12H), 7.76 (br s, 8H), 7.02 (br d, *J* = 4.8 Hz, 4H), 6.40 (d, *J* = 7.9 Hz, 8H), 6.04 (d, *J* = 7.9 Hz, 8H), 5.83 (d, *J* = 9.3 Hz, 8H), 5.79 (d, *J* = 9.0 Hz, 8H), 5.70 (d, *J* = 8.5 Hz, 8H), 5.65–5.61 (m, 12H), 5.54 (d, *J* = 8.2 Hz, 4H), 5.24 (d, *J* = 6.6 Hz, 8H), 5.14 (d, *J* = 6.0 Hz, 8H), 5.04 (d, *J* = 5.5 Hz, 8H), 2.51–2.46 (m, 8H), 2.39–2.35 (m, 8H), 2.35–2.32 (m, 4H), 1.61 (br s, 72H), 1.60 (br s, 36H), 1.56 (br s, 36H), 1.54 (br s, 72H), 1.52–1.47 (m, 144H).

Due to the effective D_{2h} symmetry of the ***c*-P$_{t\text{-Bu}}$10•(T6)$_2$** complex (on the ^{1}H NMR timescale), only one quarter of the molecule needs to be considered in the interpretation of the ^{1}H NMR spectrum (Figure S13). The chemical structure in Figure S13 represents the three distinct porphyrin units contained in this quarter. The portion of the aryl side group that points away from the template is drawn in bold and the portion of the template that coordinates the porphyrin units is also drawn. The full assignment of the ^{1}H NMR spectrum for the ***c*-P$_{t\text{-Bu}}$10•(T6)$_2$** complex is outlined below. This was completed by comparison with the complex ***c*-P$_{t\text{-Bu}}$8•(T6)$_2$** (presented above) and the locked complex ***c*-P$_{t\text{-Bu}}$10•(T6)$_2$PdCl$_2$** (presented in the next section) with the assistance of COSY and ROESY NMR experiments.

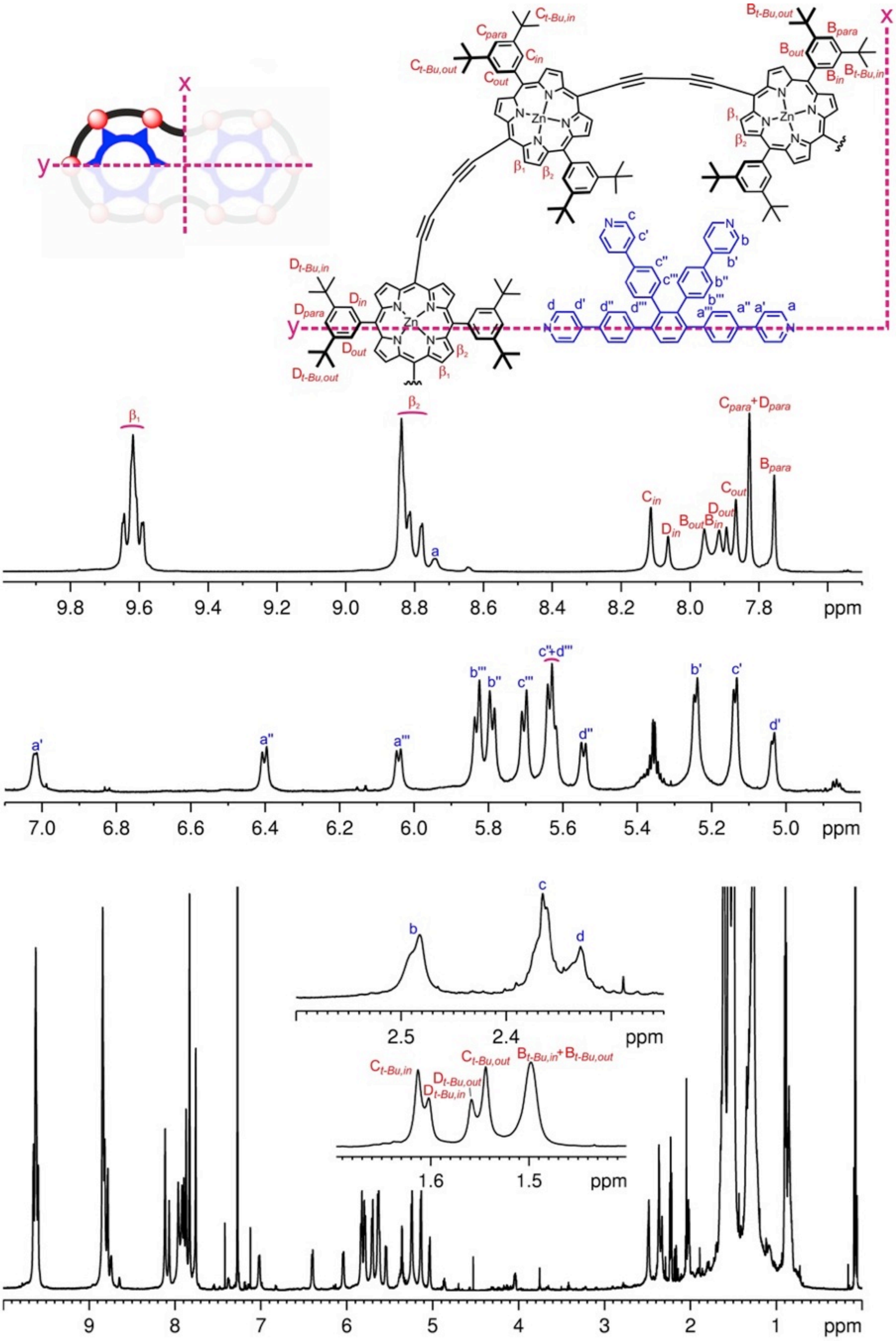

Figure S13. ^{1}H NMR spectrum of ***c*-P$_{t\text{-Bu}}$10•(T6)$_2$** (700 MHz, $CDCl_3$, 298 K) with expansions of key regions.

As in the case of ***c*-P$_{t\text{-Bu}}$8•(T6)$_2$**, the highest chemical shifts in the ^{1}H NMR spectrum of the complex ***c*-P$_{t\text{-Bu}}$10•(T6)$_2$** correspond to the β-protons on the porphyrin units, followed by the aryl protons, template protons and finally *tert*-butyl protons (Figure S13). Due to the ring current effect, the α-protons on the pyridine moieties are strongly shielded and appear at around 2.4 ppm.

The full ^{1}H NMR assignment was first done for the **T6** molecules of ***c*-P$_{t\text{-Bu}}$10•(T6)$_2$**. The following assignments are based on the assumption that the most distinct template proton ***a*** of the unbound pyridine unit corresponds to the highest frequency template proton (signal at 8.74 ppm) similar to the case of ***c*-P$_{t\text{-Bu}}$8•(T6)$_2$** (proton ***m***, Figure S9) discussed above in Section C2.

First, from the template proton ***a*,** and via a NOESY experiment showing exchange correlations, we can confirm the assignment of its exchange partners ***b***, ***c*** and ***d*** (Figure S14). The distinction between protons ***b*** and ***c*** was based on the slightly higher intensity of the cross-peak for the former. The distinction between protons ***c*** and ***d*** was based on the lower integral of resonance ***d***.

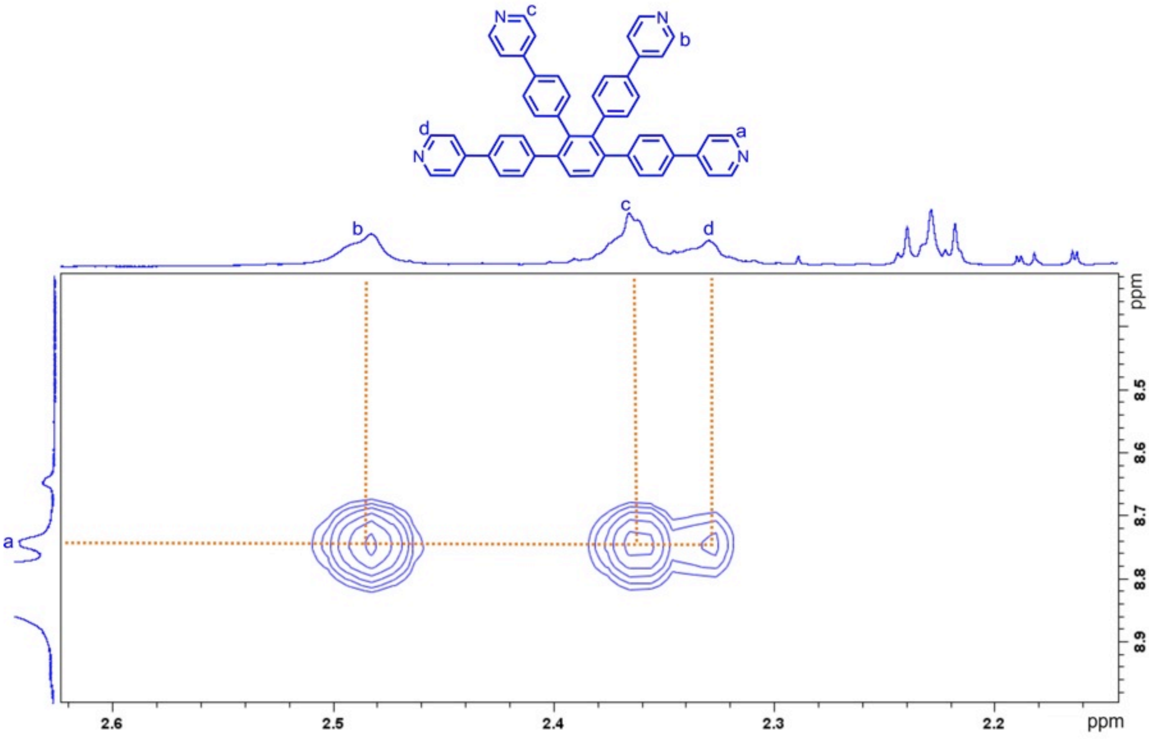

Figure S14. Region of the NOESY spectrum of ***c*-P$_{t\text{-Bu}}$10•(T6)$_2$** showing exchange correlations for proton ***a*** with protons ***b***, ***c*** and ***d*** on **T6** (700 MHz, $CDCl_3$, 298 K, 300 ms mixing time).

A COSY experiment established the assignments for protons ***b'***, ***c'*** and ***d'*** based on their coupling with protons ***b***, ***c*** and ***d*,** respectively (Figure S15).

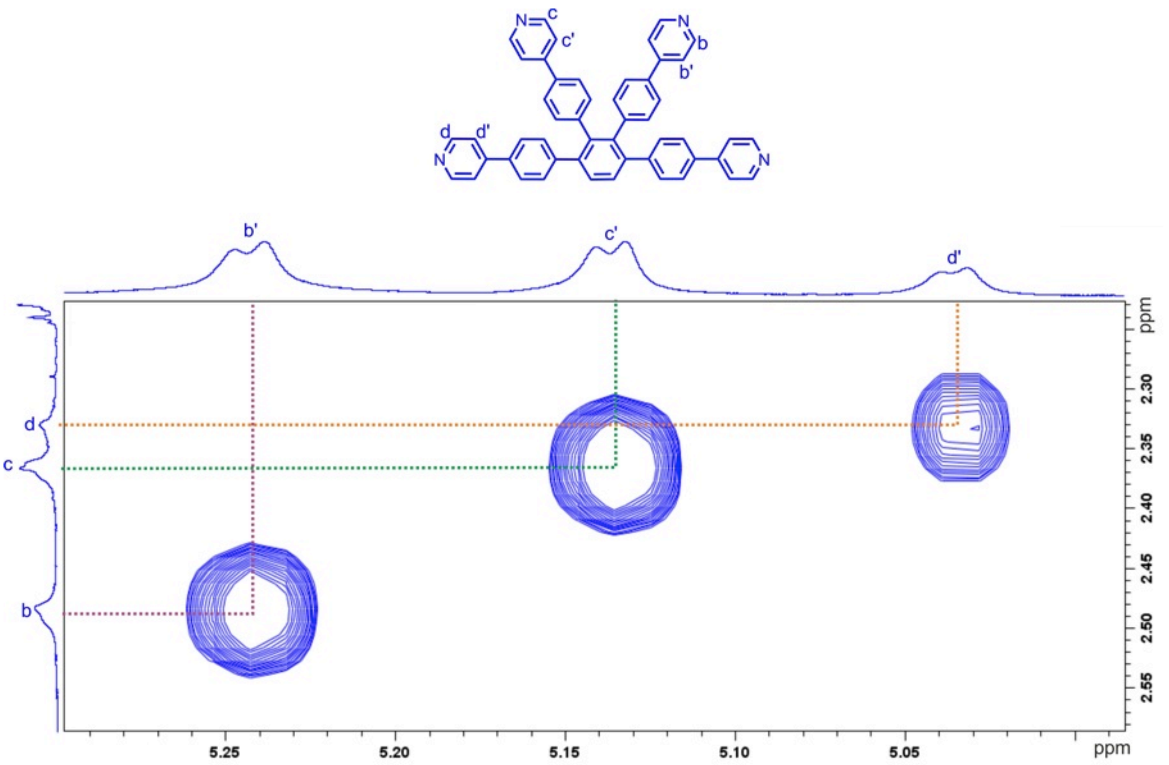

Figure S15. Region of the COSY spectrum of ***c*-P$_{t\text{-Bu}}$10•(T6)$_2$** showing correlations for protons ***b***, ***c*** and ***d*** with protons ***b'***, ***c'*** and ***d'*** on **T6** (700 MHz, $CDCl_3$, 298 K).

Based on the exchange correlations of protons ***b'***, ***c'*** and ***d'*** in the NOESY spectrum, the position of proton ***a'*** was determined as 7.02 ppm (Figure S16).

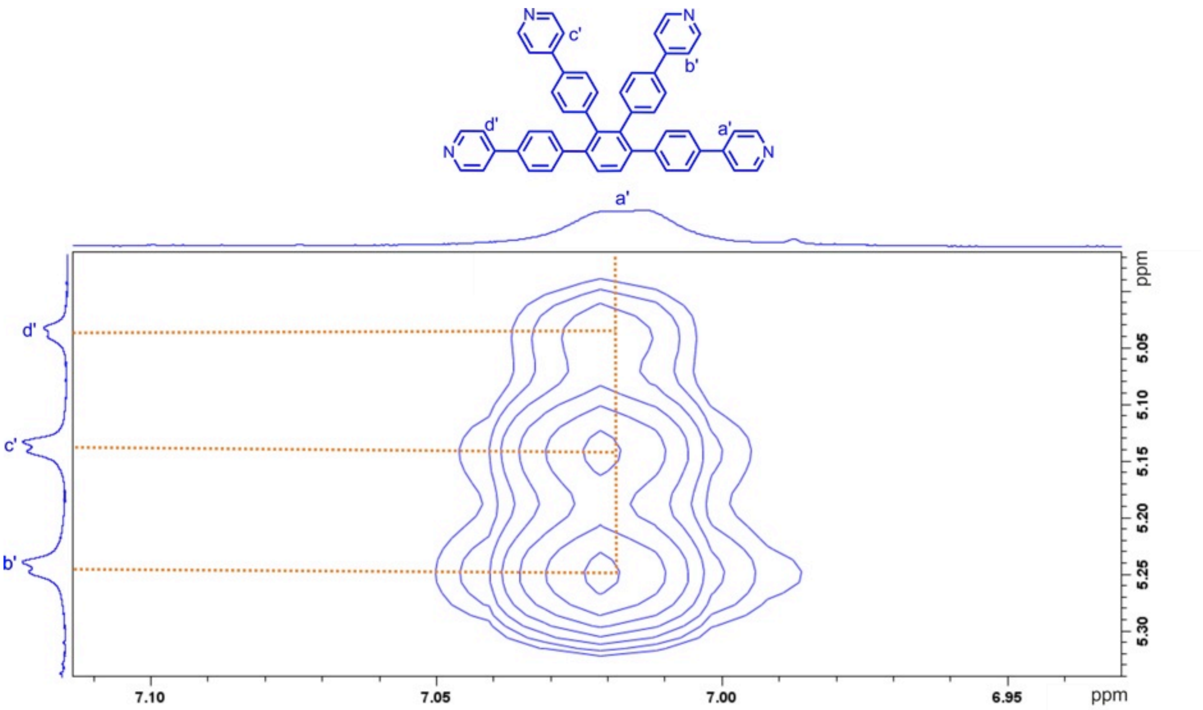

Figure S16. Region of the NOESY spectrum of ***c*-P$_{t\text{-Bu}}$10•(T6)$_2$** showing exchange correlations for protons ***b'***, ***c'*** and ***d'*** with proton ***a'*** on **T6** (700 MHz, $CDCl_3$, 298 K, 300 ms mixing time).

Assignment of proton ***a''*** was deduced by the presence of an NOE with proton ***a'*** (Figure S17).

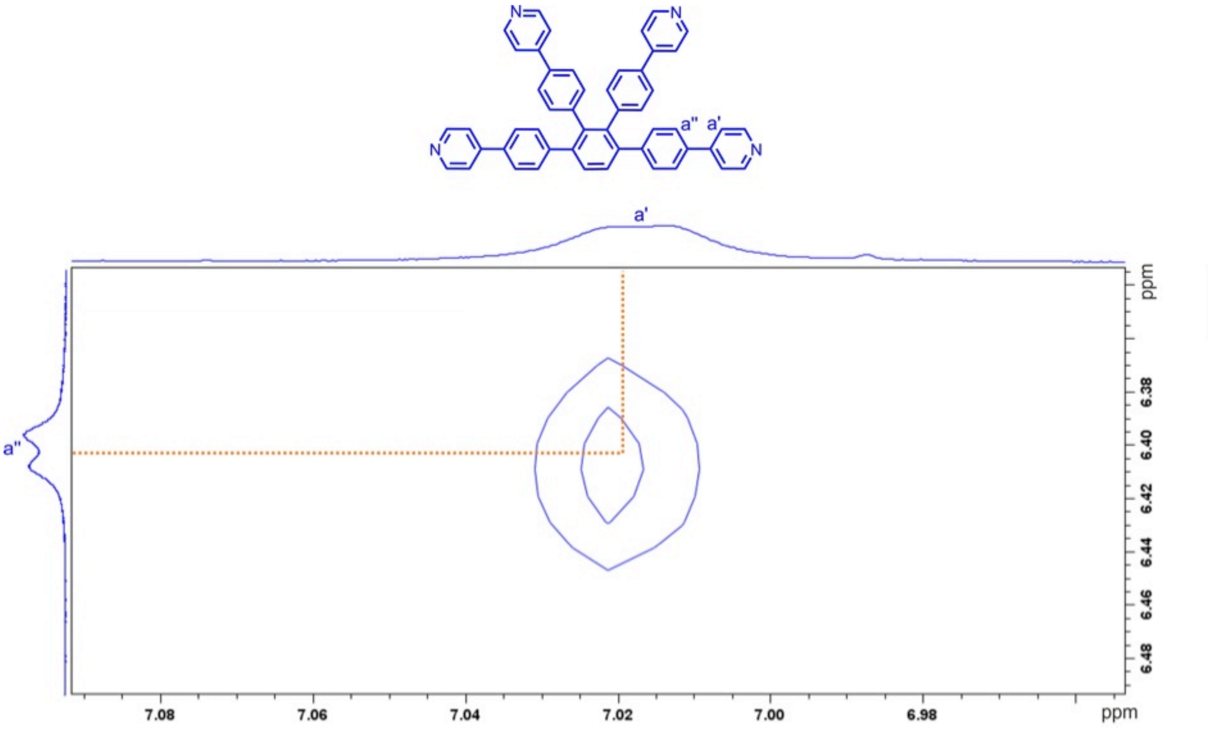

Figure S17. Region of the NOESY spectrum of ***c*-P$_{t\text{-Bu}}$10•(T6)$_2$** showing the NOE correlation of proton ***a'*** with proton ***a''*** on **T6** (700 MHz, $CDCl_3$, 298 K, 300 ms mixing time).

A COSY experiment confirmed the assignment for proton ***a'''*** based on its coupling with proton ***a''*** (Figure S18).

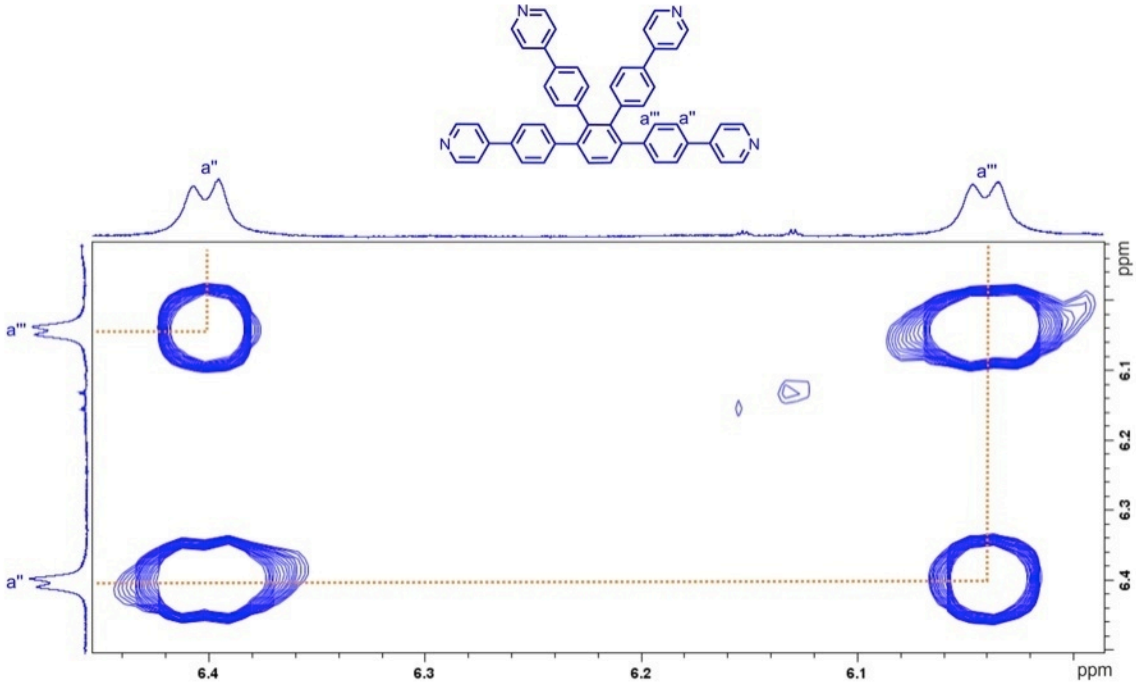

Figure S18. Region of the COSY spectrum of ***c*-P$_{t\text{-Bu}}$10•(T6)$_2$** showing correlations for proton ***a''*** and proton ***a'''*** on **T6** (700 MHz, $CDCl_3$, 298 K).

Proton ***d''*** overlaps with the signal of one of the remaining template protons (***c''*** or ***c'''***) as indicated by its integral value (Figure S19). Assignment of proton ***d''*** was additionally confirmed by the presence of an NOE with proton ***d'*** (Figure S19). The remaining proton signal with half of the integral value of proton ***a'''*** was assigned to ***d'''*** at 5.56 ppm.

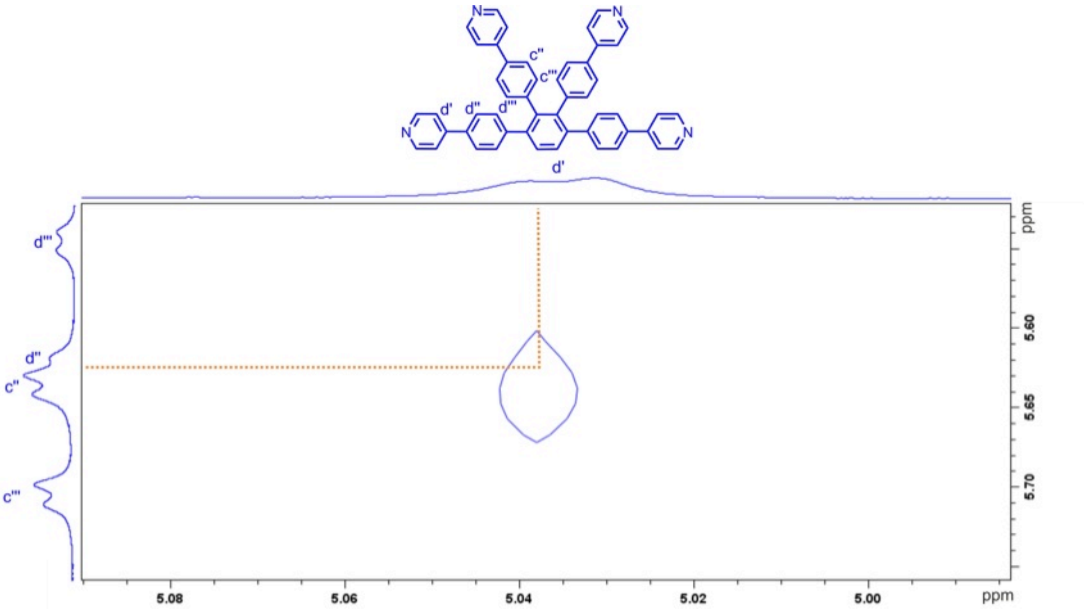

Figure S19. Region of the NOESY spectrum of ***c*-P$_{t\text{-Bu}}$10•(T6)$_2$** showing the NOE correlation of proton ***d'*** with proton ***d''*** on **T6** (700 MHz, $CDCl_3$, 298 K, 300 ms mixing time).

It was impossible to identify the resonances of protons ***b''***, ***c''***, ***d''***, ***b'''***, ***c'''*** and ***d'''*** based on the ROESY and COSY spectra of the complex ***c*-P$_{t\text{-Bu}}$10•(T6)$_2$** because the numerous exchange and NOE cross-peaks are too close and overlap at points (Figure S20). The assignment of these resonances shown in Figure S20 was deduced by comparison with the same signals in the locked complex ***c*-P$_{t\text{-Bu}}$10•(T6)$_2$•$PdCl_2$** (see later).

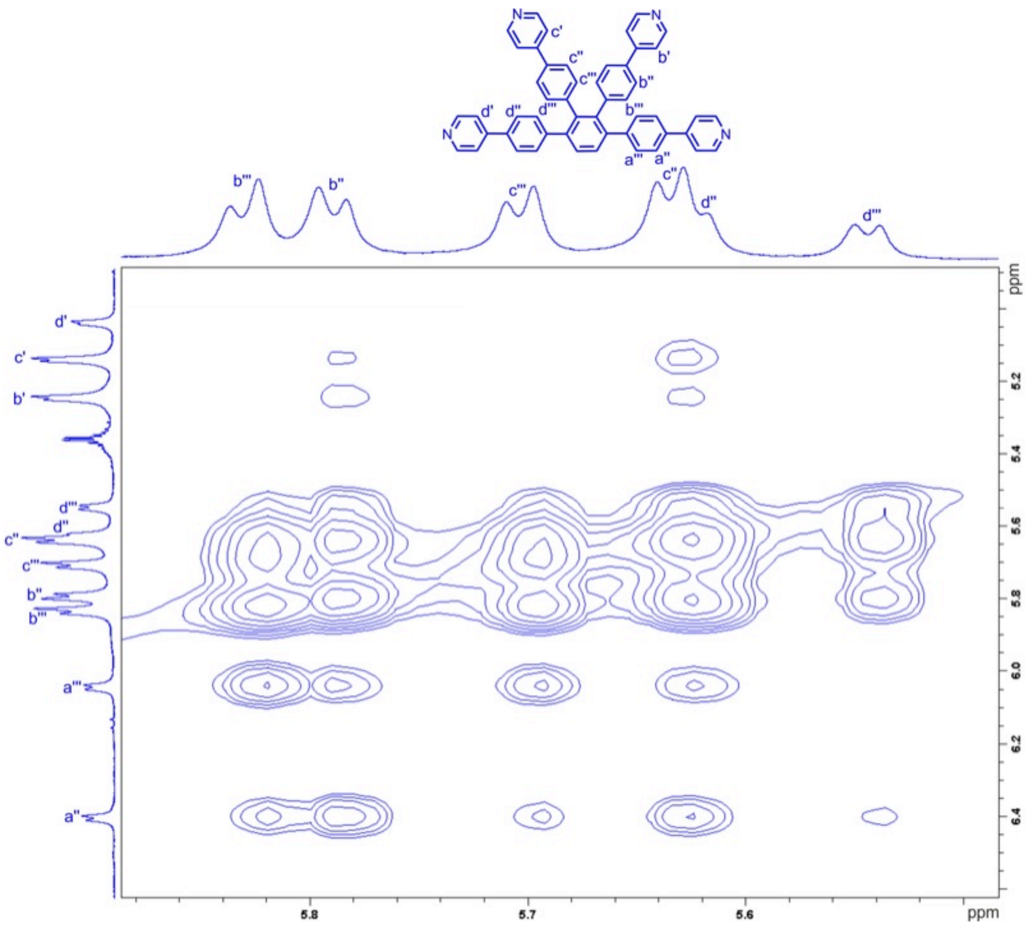

Figure S20. Region of the NOESY spectrum of ***c*-P$_{t\text{-Bu}}$10•(T6)$_2$** showing exchange correlations for protons ***b''***, ***c''*** and ***d''*** with protons ***b'''***, ***c'''*** and ***d'''*** on **T6** (700 MHz, $CDCl_3$, 298 K, 300 ms mixing time).

Following the assignment of the template protons, the identity of the aryl side-group protons was deduced. There are three porphyrin environments (porphyrin units B, C and D). Within the aryl side groups of each of these porphyrin units, there are additionally three environments corresponding to inward and outward facing *ortho*-protons as well as *para*-protons (Figure S13). The portion of the aryl side group that points away from the template is drawn in bold and the portion of the template that coordinates the porphyrin units is also drawn.

Assignment of protons **B*in***, **C*in*** and **D*in*** based on the NOE correlations with protons ***b***, ***c*** and ***d*** was impossible due to the presence of exchange correlations (Figure S21). The assignment of these resonances was deduced by comparison with the same signals in the locked complex ***c*-P$_{t\text{-Bu}}$10•(T6)$_2$•PdCl$_2$**.

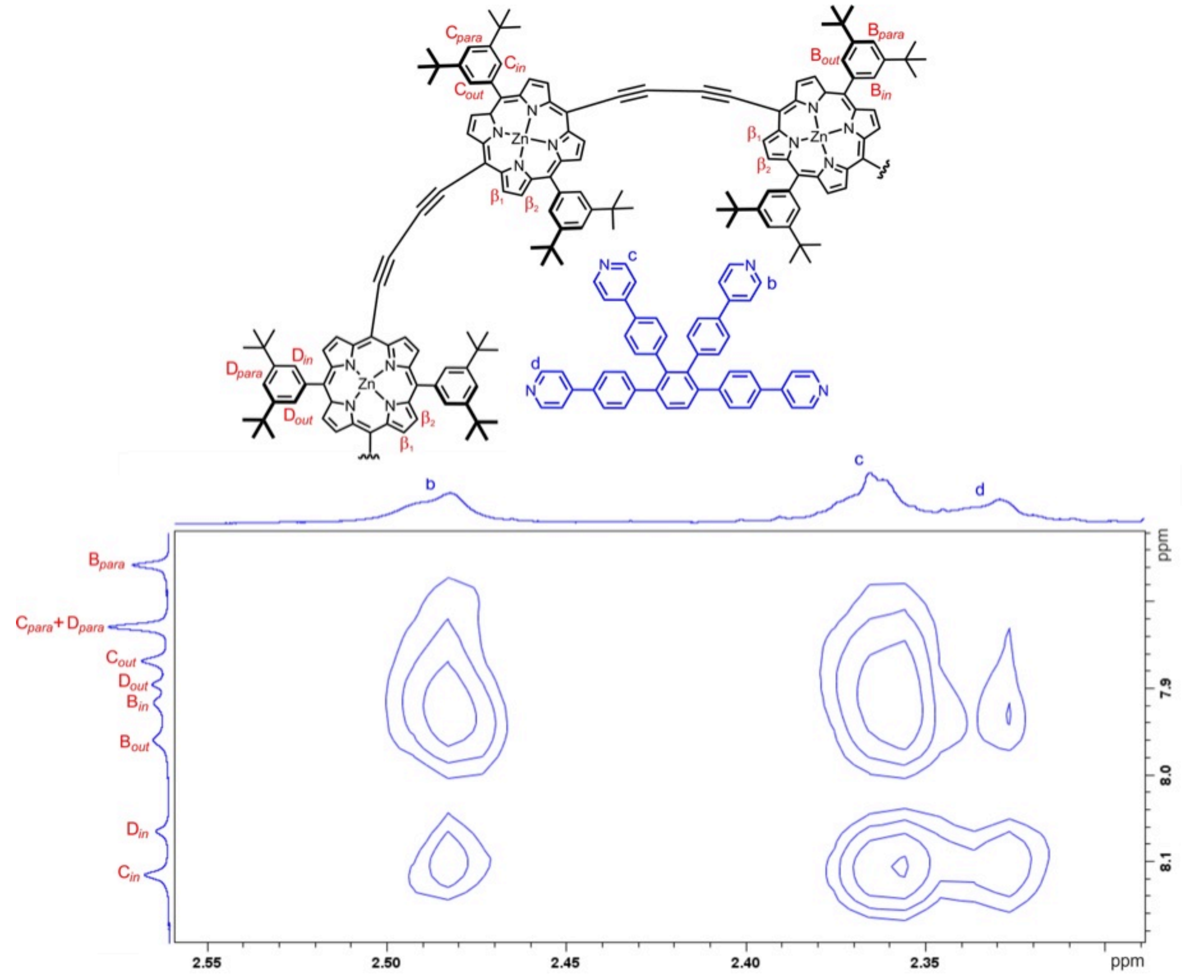

Figure S21. Region of the NOESY spectrum of ***c*-P$_{t\text{-Bu}}$10•(T6)$_2$** showing NOEs and exchange correlations of protons ***b***, ***c*** and ***d*** on **T6** and protons ***C$_{in}$*** and ***D$_{in}$*** on the aryl side groups of the porphyrin units (700 MHz, $CDCl_3$, 298 K, 300 ms mixing time).

The assignment of protons **B$_{para}$**, **C$_{para}$** and **D$_{para}$** was based on the comparison of their corresponding integrals and by assuming that protons **C$_{para}$** and **D$_{para}$** show up at the same chemical shift (7.83 ppm) and are significantly different compared to **B$_{para}$** (7.76 ppm) (Figure S22). Resonance **D$_{out}$** (7.89 ppm) was assigned by comparing its integration with that of resonance **D$_{in}$**. The assignment of resonances **B$_{out}$** (7.96 ppm), **B$_{in}$** (7.92 ppm) and **C$_{out}$** (7.87 ppm) was deduced by comparison with the same signals in the locked complex ***c*-P$_{t\text{-Bu}}$10•(T6)$_2$•PdCl$_2$**.

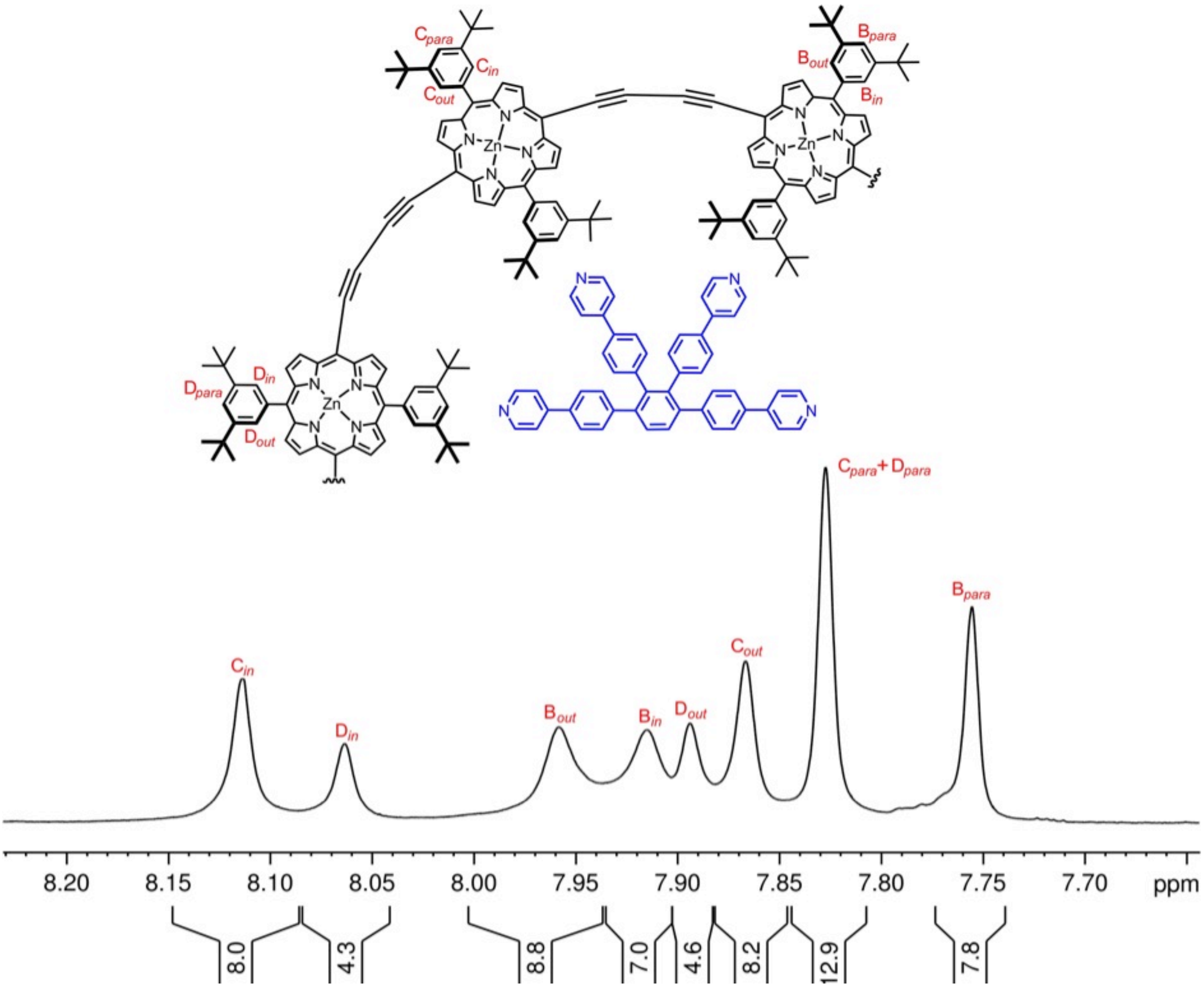

Figure S22. Region of the ^{1}H NMR spectrum of ***c*-P$_{t\text{-Bu}}$10•(T6)$_2$** showing the integral values of protons **B$_{in}$**, **B$_{out}$**, **B$_{para}$**, **C$_{in}$**, **C$_{out}$**, **C$_{para}$**, **D$_{in}$**, **D$_{out}$** and **D$_{para}$** of the aryl side groups of the porphyrin units (700 MHz, $CDCl_3$, 298 K).

The assignment of *tert*-butyl protons **B$_{t\text{-}Bu,in}$**, (1.50 ppm), **B$_{t\text{-}Bu,out}$** (1.50 ppm), **C$_{t\text{-}Bu,in}$**, (1.61 ppm), **C$_{t\text{-}Bu,out}$** (1.54 ppm), **D$_{t\text{-}Bu,in}$**, (1.60 ppm) and **D$_{t\text{-}Bu,out}$** (1.56 ppm) was deduced by comparison with the same signals in the locked complex ***c*-P$_{t\text{-Bu}}$10•(T6)$_2$•PdCl$_2$**.

C5) Characterization of ***c*-P$_{t\text{-Bu}}$10•(T6)$_2$•PdCl$_2$**

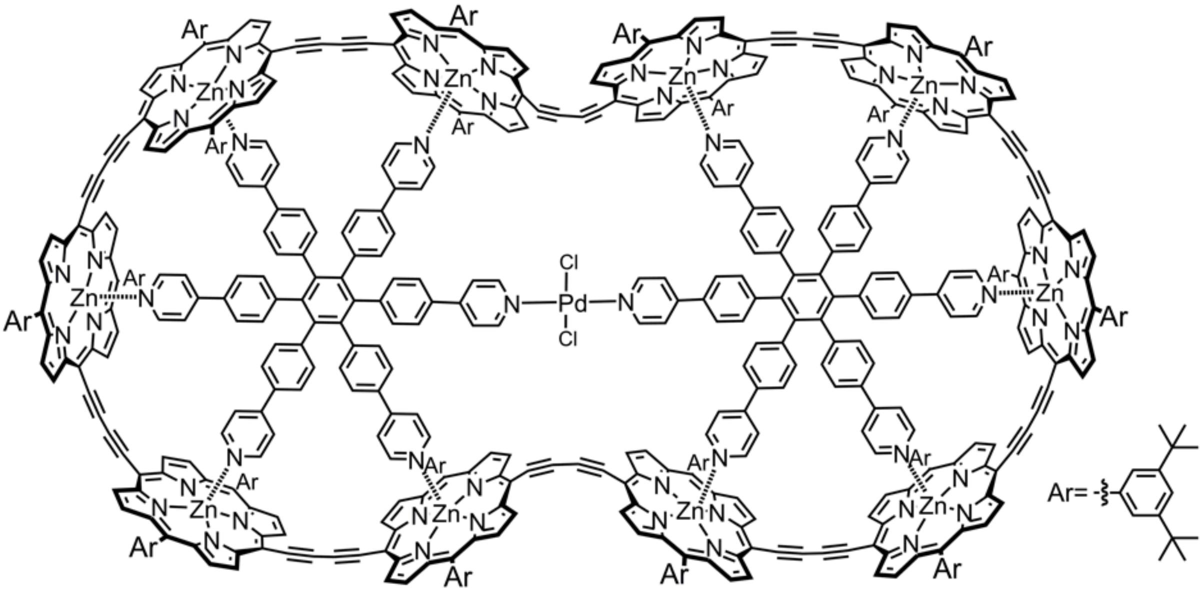

This complex was prepared by gradual addition of $PdCl_2(PhCN)_2$ to a solution of ***c*-P$_{t\text{-Bu}}$10•(T6)$_2$** in $CDCl_3$ (^{1}H NMR titration, 700 MHz, 298 K, see Figure S41).

^{1}H NMR (700 MHz, $CDCl_3$, 298 K): δ_H (ppm) 9.66–9.56 (m, 40H), 8.86–8.76 (m, 40H), 8.17 (d, J = 6.8 Hz, 4H), 8.11 (br s, 8H), 8.06 (br s, 4H), 7.95 (br s, 8H), 7.92 (br s, 8H), 7.89 (br s, 4H), 7.86 (br s, 8H), 7.82 (br s, 12H), 7.75 (br s, 8H), 6.57 (d, J = 7.1 Hz, 4H), 6.29 (d, J = 8.5 Hz, 4H), 5.94 (d, J = 8.6 Hz, 4H), 5.81 (d, J = 9.2 Hz, 8H), 5.75 (d, J = 9.2 Hz, 8H), 5.69 (d, J = 8.9 Hz, 8H), 5.64–5.59 (m, 12H), 5.54 (d, J = 8.7 Hz, 4H), 5.29 (d, J = 6.7 Hz, 8H), 5.13 (d, J = 6.5 Hz, 8H), 5.03 (d, J = 6.0 Hz, 4H), 2.47–2.43 (m, 8H), 2.36–2.34 (m, 8H), 2.33–2.31 (m, 4H), 1.61 (br s, 72H), 1.60 (br s, 36H), 1.56 (br s, 36H), 1.54 (br s, 72H), 1.52–1.48 (m, 144H).

Due to the effective D_{2h} symmetry of the ***c*-P$_{t\text{-Bu}}$10•(T6)$_2$•PdCl$_2$** complex, only one quarter of the molecule needs to be considered in the interpretation of the ^{1}H NMR spectrum (Figure S23). The chemical structure in Figure S23 represents the three distinct porphyrin units contained in this quarter. The portion of the aryl side group that points away from the template is drawn in bold and the portion of the template that coordinates the porphyrin units is also drawn. The full assignment of the ^{1}H NMR spectrum for the ***c*-P$_{t\text{-Bu}}$10•(T6)$_2$•PdCl$_2$** complex is outlined below. This was completed by comparison with the complex ***c*-P$_{t\text{-Bu}}$8•(T6)$_2$** and ***c*-P$_{t\text{-Bu}}$10•(T6)$_2$** (presented above) and as well as with the assistance of COSY and NOESY NMR experiments.

The full ^{1}H NMR assignment was first done for the **T6** molecules of ***c*-P$_{t\text{-Bu}}$10•(T6)$_2$•PdCl$_2$**. The following assignments are based on the comparison with the assignment of the template protons of ***c*-P$_{t\text{-Bu}}$10•(T6)$_2$** as well as with the assistance of COSY and NOESY NMR experiments. The most distinct template proton ***a*** of palladium-coordinated pyridine unit corresponds to the highest frequency template proton (at 8.17 ppm) similar to the case of ***c*-P$_{t\text{-Bu}}$10•(T6)$_2$** discussed above in Section C4. Due to the binding of palladium(II), the resonance of proton ***a*** shows up at lower shift (8.17 ppm) compared to the complex ***c*-P$_{t\text{-Bu}}$10•(T6)$_2$** (8.74 ppm). Pyridine resonances ***b*** (2.44 ppm), ***c*** (2.35 ppm) and ***d*** (2.32 ppm) were assigned by comparison with complex ***c*-P$_{t\text{-Bu}}$10•(T6)$_2$**.

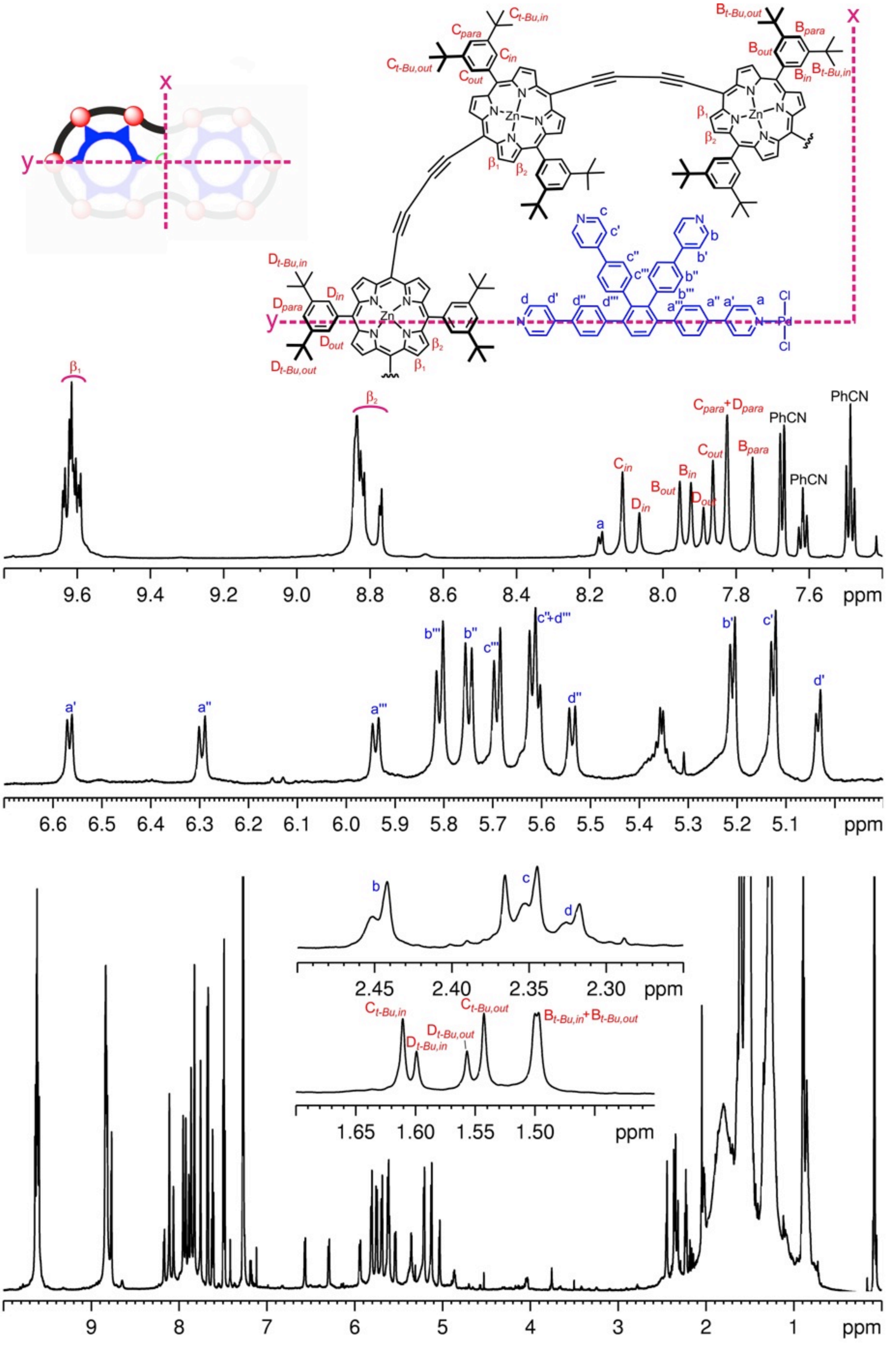

Figure S23. ^{1}H NMR spectrum of ***c*-P$_{t\text{-}Bu}$10•(T6)$_2$•PdCl$_2$** (700 MHz, $CDCl_3$, 298 K).

First, from the template protons ***a***, ***b***, ***c*** and ***d***, and via a COSY experiment, we can confirm the assignment for protons ***a'***, ***b'***, ***c'*** and ***d'*** (Figure S24).

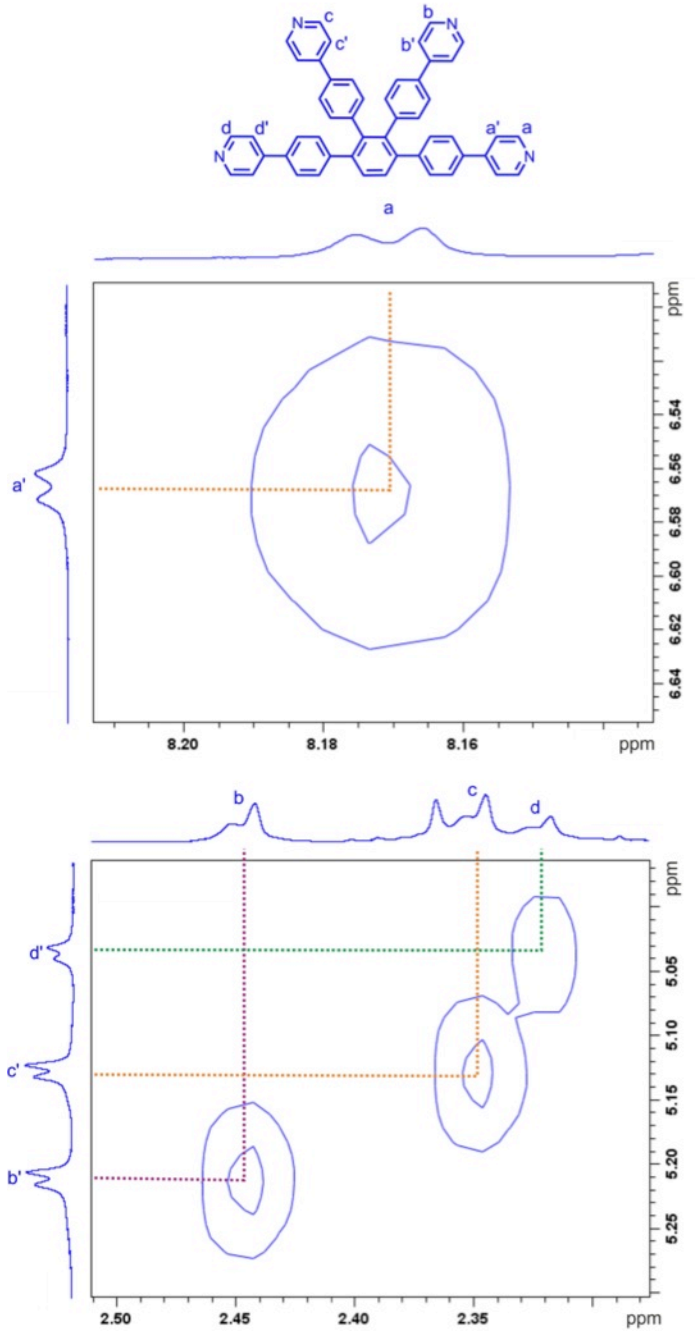

Figure S24. Regions of the COSY spectrum of ***c*-P$_{t\text{-Bu}}$10•(T6)$_2$•PdCl$_2$** showing the correlations for protons ***a***, ***b***, ***c*** and ***d*** with protons ***a'***, ***b'***, ***c'*** and ***d'*** on **T6** (700 MHz, $CDCl_3$, 298 K).

The assignment of protons ***a''***, ***b''***, ***c''*** and ***d''*** was deduced by the presence of NOE correlations with protons ***a'***, ***b'***, ***c'*** and ***d'***, respectively (Figure S25).

A COSY experiment led to the assignments of protons ***a'''***, ***b'''***, ***c'''*** and ***d'''*** based on their coupling with protons ***a''***, ***b''***, ***c''*** and ***d''***, respectively (Figure S26).

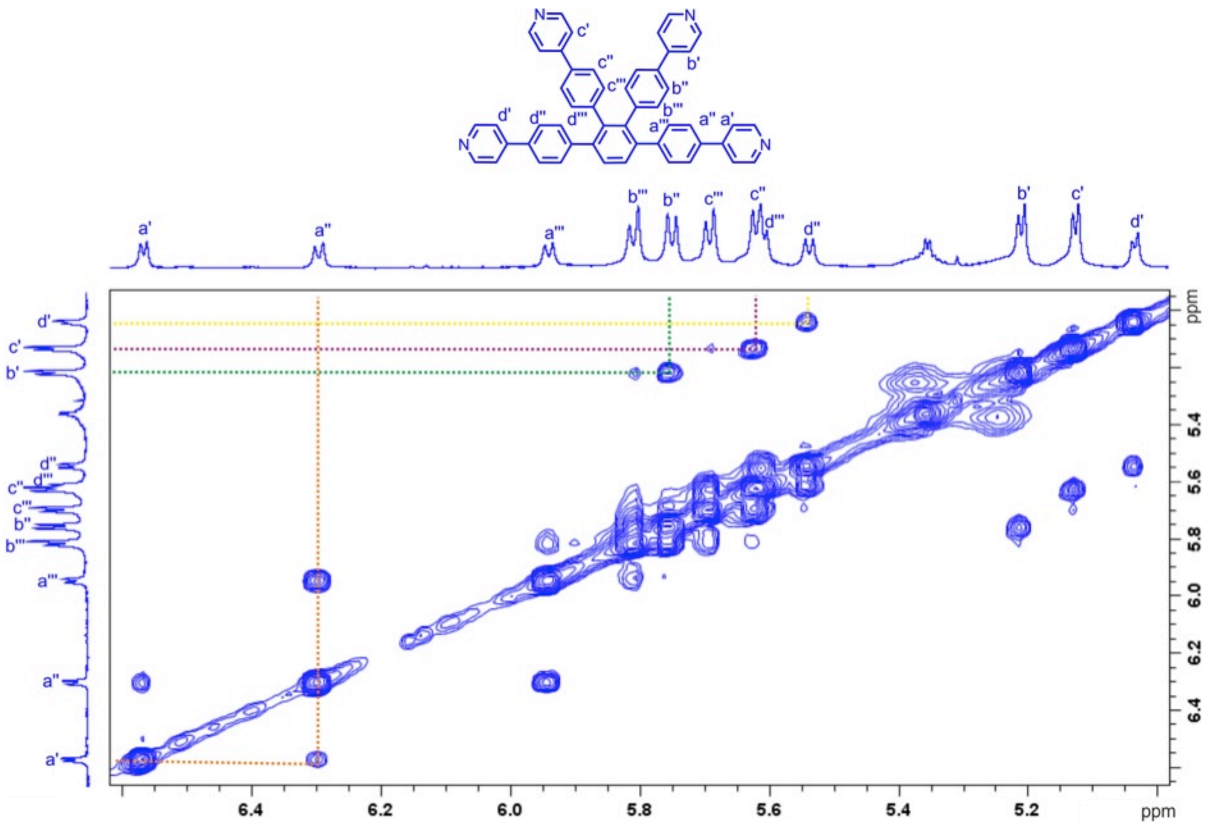

Figure S25. Region of the NOESY spectrum of ***c*-P$_{t\text{-Bu}}$10•(T6)$_2$•PdCl$_2$** showing the NOE correlations of protons ***a’***, ***b’***, ***c’*** and ***d’*** with protons ***a’’***, ***b’’***, ***c’’*** and ***d’’***, respectively (700 MHz, $CDCl_3$, 298 K, 200 ms mixing time).

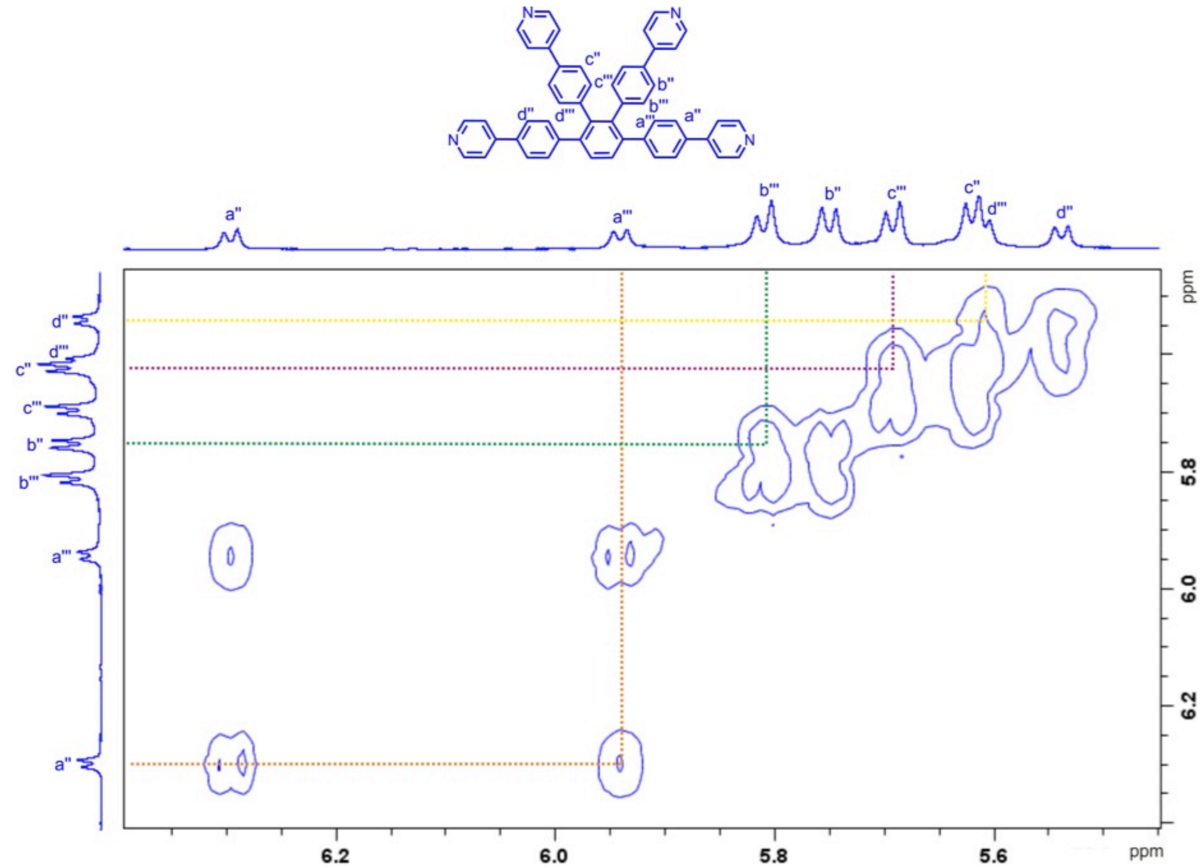

Figure S26. Region of the COSY spectrum of ***c*-P$_{t\text{-Bu}}$10•(T6)$_2$•PdCl$_2$** showing the correlations of protons ***a’’***, ***b’’***, ***c’’*** and ***d’’*** with protons ***a’’’***, ***b’’’***, ***c’’’*** and ***d’’’***, respectively (700 MHz, $CDCl_3$, 298 K).

Following the assignment of the template protons, the identity of the three aryl side-group protons was deduced. There are three porphyrin environments (porphyrin units B, C and D). Within the aryl side groups of each of these porphyrin units, there are additionally three environments corresponding to inward and outward facing *ortho*-protons as well as *para*-protons (Figure S23). The portion of the aryl side group that points away from the template is drawn in bold and the portion of the template that coordinates the porphyrin units is also drawn.

The assignment of protons **B_{in}**, **C_{in}** and **D_{in}** is based on the NOE correlations with protons ***b***, ***c*** and ***d*** (Figure S27).

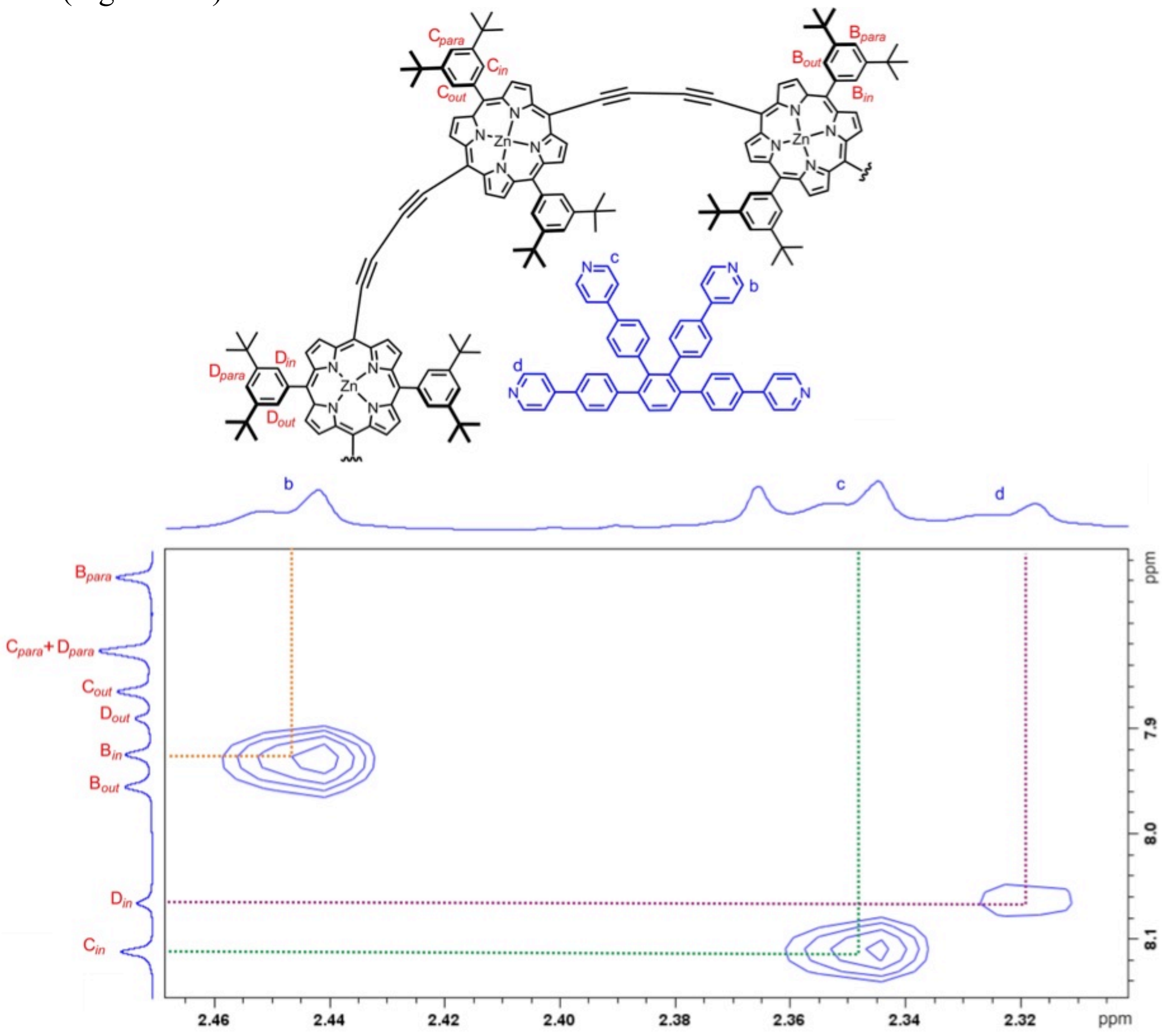

Figure S27. Region of the NOESY spectrum ***c*-P$_{t\text{-Bu}}$10•(T6)$_2$•PdCl$_2$** showing the NOE correlations of protons ***b***, ***c*** and ***d*** on **T6** with protons ***B_{in}***, ***C_{in}*** and ***D_{in}*** on the aryl side groups of the porphyrin units (700 MHz, $CDCl_3$, 298 K, 200 ms mixing time).

The assignment of protons **B_{para}**, **C_{para}** and **D_{para}** was based on the comparison of their corresponding integrals and by assuming that protons **C_{para}** and **D_{para}** show up at the same chemical shift (7.82 ppm) and are significantly different compared to **B_{para}** (7.76 ppm) (Figure S28). Resonance **B_{out}** (7.95 ppm) was assigned based on a weak NOE correlation with resonance **B_{para}**. Resonance **C_{out}** (7.86 ppm) was assigned based on a NOE correlation with resonance **C_{in}**. Finally, resonance **D_{out}** (7.89 ppm) was assigned based on a NOE

correlation with resonance **D*in***. as well as comparing its integration with that of resonance **D*in***.

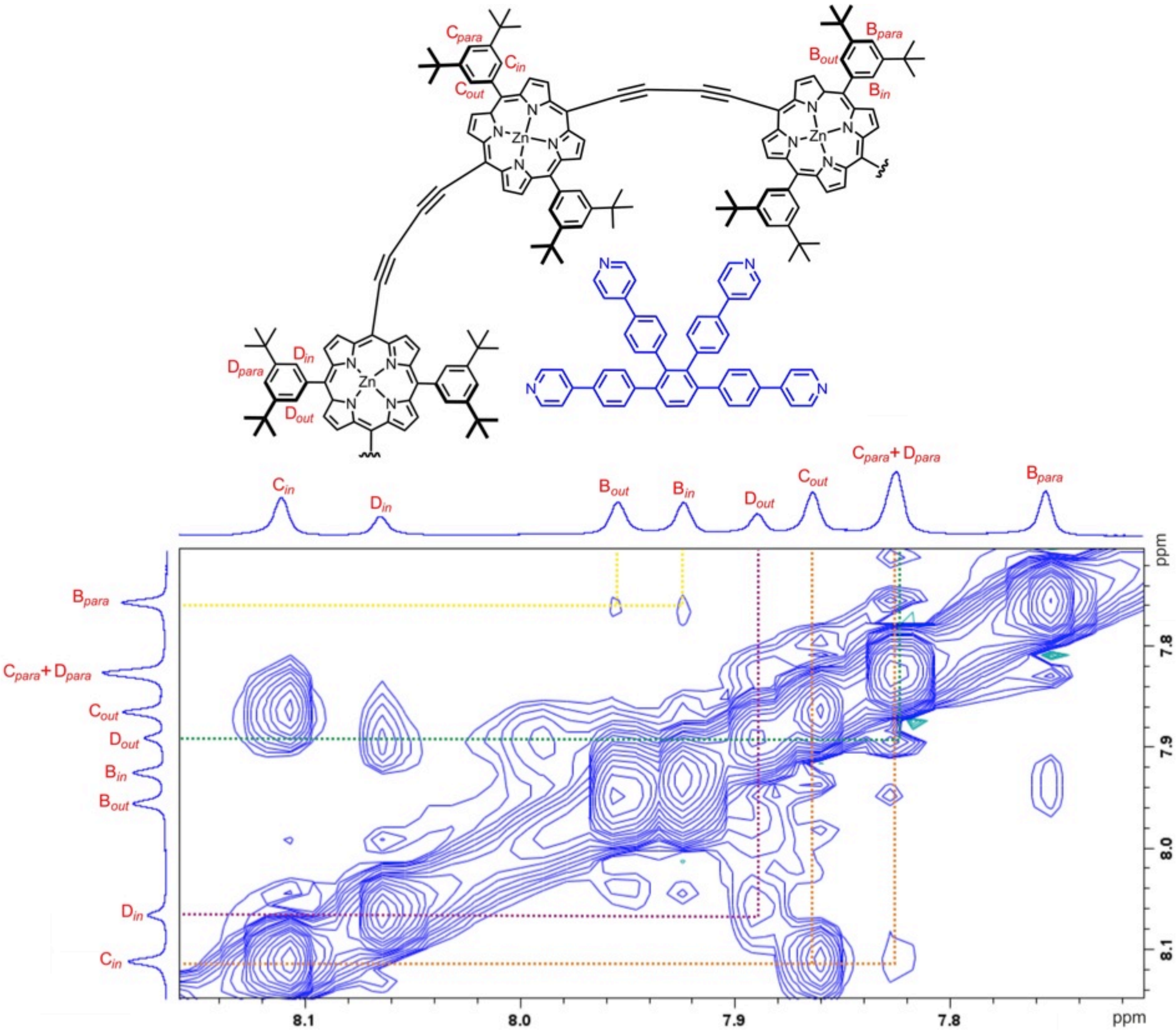

Figure S28. Region of the NOESY spectrum showing the Exchange correlations of protons **B*in***, **B*out***, **B*para***, **C*in***, **C*out***, **C*para***, **D*in***, **D*out*** and **D*para*** of the aryl side groups of the porphyrin units (700 MHz, $CDCl_3$, 298 K, 200 ms mixing time).

The assignment of the *tert*-butyl protons **B*t-Bu,in*** (1.50 ppm), **B*t-Bu,out*** (1.50 ppm), **C*t-Bu,in*** (1.61 ppm), **C*t-Bu,out*** (1.54 ppm), **D*t-Bu,in***, (1.60 ppm) and **D*t-Bu,out*** (1.56 ppm) was confirmed by observing NOE signals with protons **B*in***, **B*out***, **C*in***, **C*out***, **D*in*** and **D*out***, respectively (Figure S29).

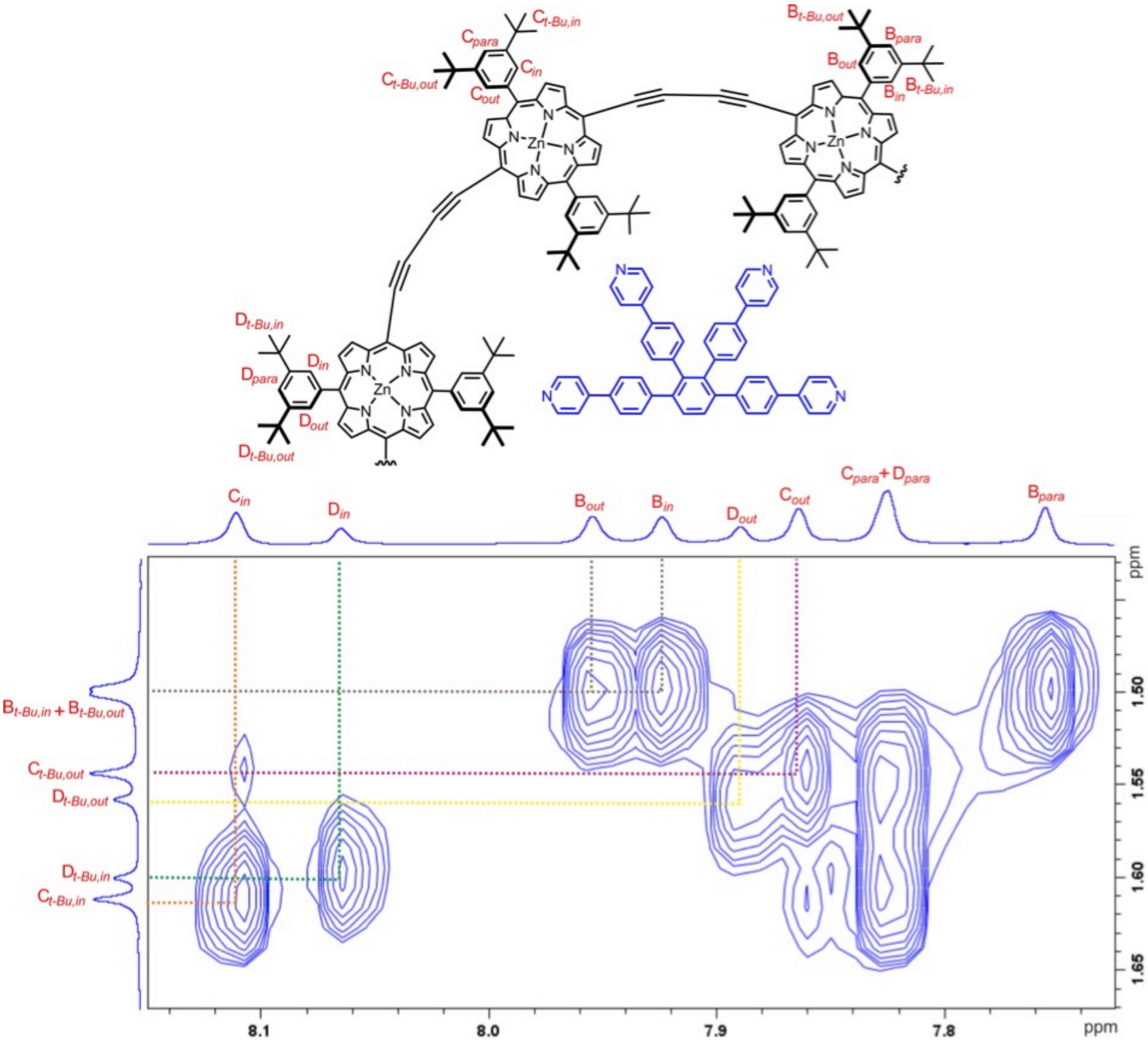

Figure S29. Region of the NOESY spectrum showing the NOE correlations of protons $\mathbf{B}_{t\text{-}Bu,in}$, $\mathbf{B}_{t\text{-}Bu,out}$, $\mathbf{C}_{t\text{-}Bu,in}$, $\mathbf{C}_{t\text{-}Bu,out}$, $\mathbf{D}_{t\text{-}Bu,in}$, $\mathbf{D}_{t\text{-}Bu,out}$ with protons $\mathbf{B}_{in}$, $\mathbf{B}_{out}$, $\mathbf{B}_{para}$, $\mathbf{C}_{in}$, $\mathbf{C}_{out}$, $\mathbf{C}_{para}$, $\mathbf{D}_{in}$, $\mathbf{D}_{out}$ and $\mathbf{D}_{para}$ of the aryl side groups of the porphyrin units with (700 MHz, $CDCl_3$, 298 K, 200 ms mixing time).

C6) Comparison of Δδ for the template protons in the [1]*H NMR of* ***c*-P$_{t\text{-Bu}}$10•(T6)$_2$** *and* ***c*-P$_{t\text{-Bu}}$10•(T6)$_2$•PdCl$_2$**

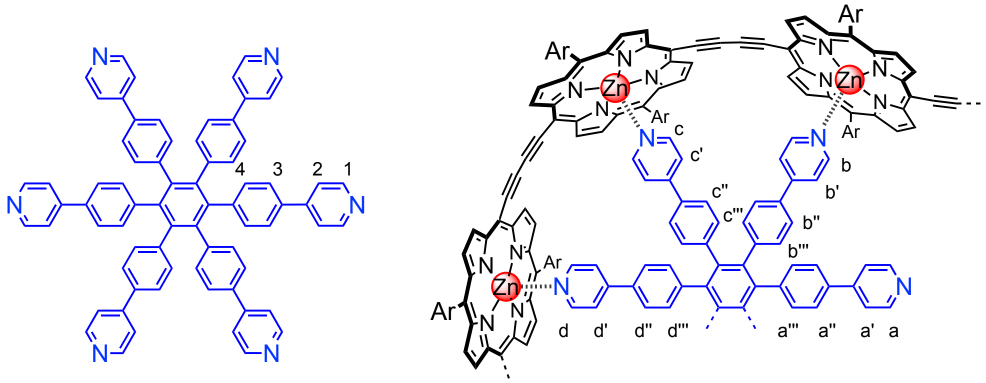

Figure S30. Proton labels for **T6** and complex-bound **T6** in ***c*-P$_{t\text{-Bu}}$10•(T6)$_2$** and in ***c*-P$_{t\text{-Bu}}$10•(T6)$_2$•PdCl$_2$**.

Table S3. ^{1}H NMR chemical shifts for protons on free **T6** and the corresponding protons on bound **T6** in ***c*-P$_{t\text{-Bu}}$10•(T6)$_2$** and in ***c*-P$_{t\text{-Bu}}$10•(T6)$_2$•PdCl$_2$** ($CDCl_3$, 298 K).

**T6**	δ_H (ppm)	***c*-P$_{t\text{-Bu}}$10•(T6)$_2$**	δ_H (ppm)	$\Delta\delta_H$ **(ppm)**	***c*-P$_{t\text{-Bu}}$10•(T6)$_2$•PdCl$_2$**	δ_H (ppm)	$\Delta\delta_H$ **(ppm)**
**1**	8.54	**a**	8.74	**–0.20**	**a**	8.17	**0.37**
		**b**	2.49	**6.05**	**b**	2.45	**6.09**
		**c**	2.37	**6.17**	**c**	2.35	**6.19**
		**d**	2.33	**6.21**	**d**	2.32	**6.22**
**2**	7.34	**a'**	7.02	**0.32**	**a'**	6.57	**0.77**
		**b'**	5.24	**2.10**	**b'**	5.21	**2.13**
		**c'**	5.14	**2.20**	**c'**	5.13	**2.21**
		**d'**	5.04	**2.30**	**d'**	5.03	**2.31**
**3**	7.23	**a''**	6.40	**0.83**	**a''**	6.30	**0.93**
		**b''**	5.83	**1.40**	**b''**	5.75	**1.48**
		**c''**	5.63	**1.60**	**c''**	5.62	**1.61**
		**d''**	5.54	**1.69**	**d''**	5.54	**1.69**
**4**	7.00	**a'''**	6.04	**0.96**	**a'''**	5.94	**1.06**
		**b'''**	5.83	**1.17**	**b'''**	5.81	**1.19**
		**c'''**	5.70	**1.30**	**c'''**	5.69	**1.31**
		**d'''**	5.63	**1.37**	**d'''**	5.61	**1.39**

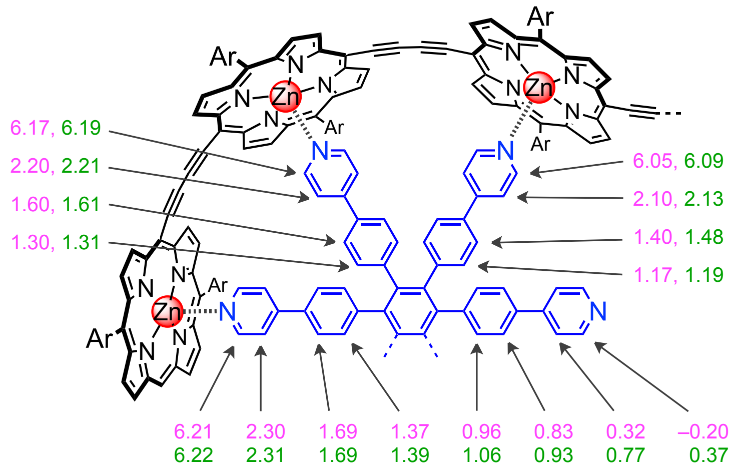

Figure S31. Complexation-induced changes in chemical shift, *Δδ* in the ^{1}H NMR spectra of free template **T6** and bound templates (pink for ***c*-P$_{t\text{-Bu}}$10•(T6)$_2$** and green for ***c*-P$_{t\text{-Bu}}$10•(T6)$_2$•PdCl$_2$**) in $CDCl_3$, at 298 K.

C7) Analytical GPC traces

Crude reaction mixtures were analyzed by GPC (see Section A for details of instrumentation). In the case of R = *t*-Bu compounds, the solution was passed over a short column (1-3 cm) of basic alumina, to remove the palladium catalyst, and a SEC column (S-X1 Biobeads) in $CHCl_3$/10% pyridine or toluene/10% pyridine, to remove the benzoquinone and templates. For R = OC_8H_{17}, only the SEC column was performed to remove catalyst, benzoquinone and templates. Analytical yields were estimated by integrating GPC traces using the extinction coefficient at the detection wavelength (500 nm). The treatment described above removes the templates from the nanorings in all cases except **T6** and **T5/T5*** in ***c*-P6**.

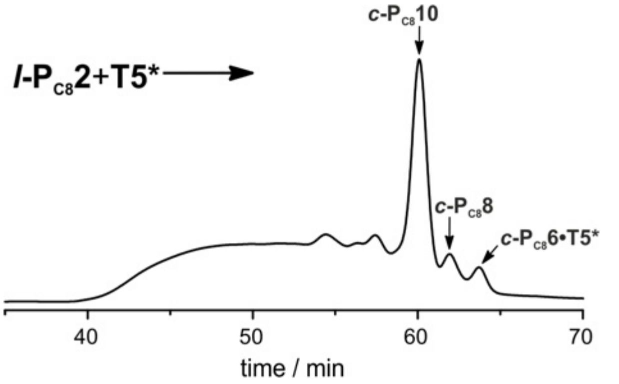

Figure S32. Analytical GPC traces (toluene/1% pyridine, detection at 500 nm) of the crude reaction mixture of coupling ***l*-P$_{t\text{-Bu}}$2** in the presence of **T5***.

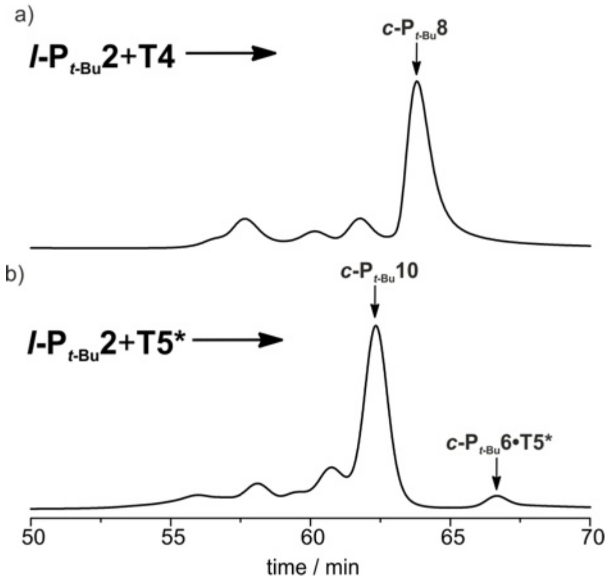

Figure S33. Analytical GPC traces (toluene/1% pyridine, detection at 500 nm) of the crude reaction mixtures of coupling ***l*-P$_{t\text{-Bu}}$2** in the presence of **T4** (a) and **T5*** (b).

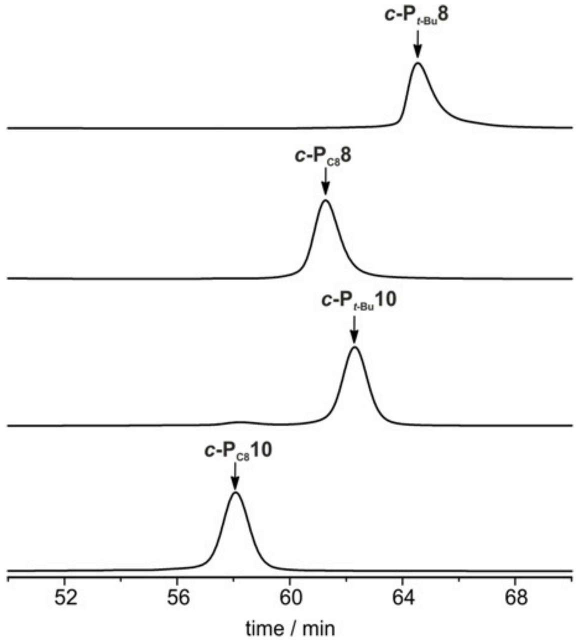

Figure S34. Analytical GPC traces (toluene/1% pyridine, detection at 500 nm) of the purified nanorings ***c*-P$_{t\text{-Bu}}$8**, ***c*-P$_{C8}$8**, ***c*-P$_{t\text{-Bu}}$10** and ***c*-P$_{c8}$10**.

D. Binding Studies of Templates within Nanorings

D1) Binding of **T4** *with* ***c*-P8**

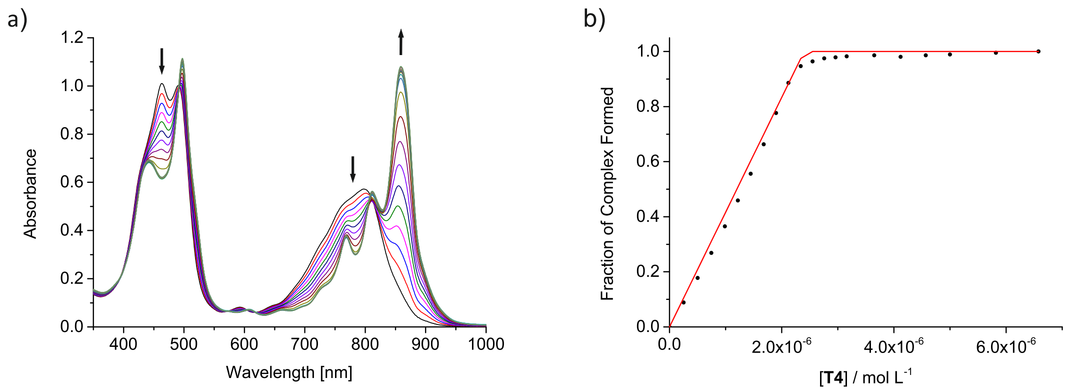

Figure S35. UV/vis/NIR titration of ***c*-P$_{t\text{-Bu}}$8** ([***c*-P$_{t\text{-Bu}}$8**] = 1.2 × 10^{-6} mol L^{-1}) with **T4** in $CHCl_3$ at 298 K. (a) Changes in absorption upon addition of **T4**. Arrows indicate areas of increasing and decreasing absorption during the titration; (b) Binding isotherm (black dots) derived from absorption data at λ = 860 nm and calculated fit (red line). A square end-point is reached at 2:1 **T4**:***c*-P$_{t\text{-Bu}}$8** ratio.

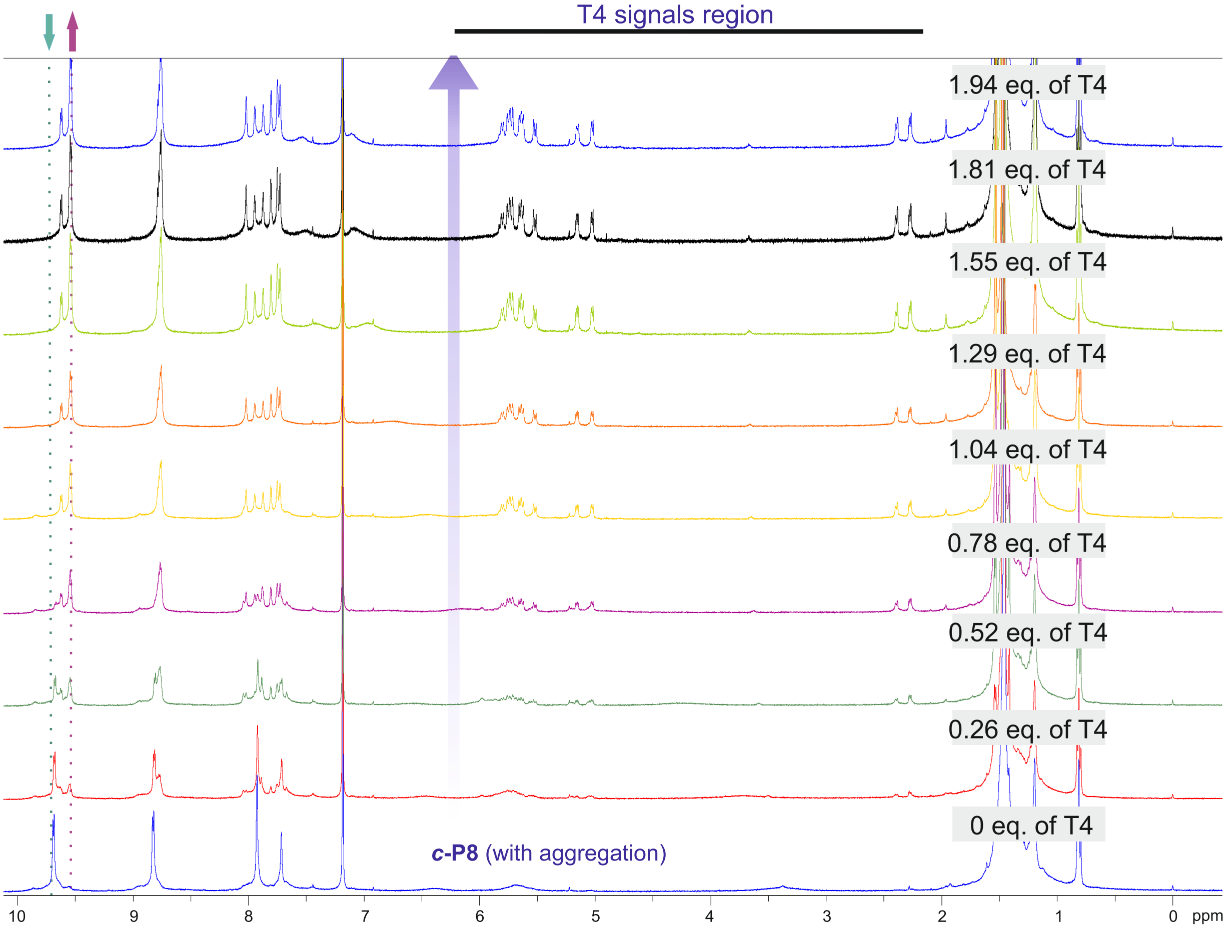

Figure S36. ^{1}H NMR titration of ***c*-P$_{t\text{-Bu}}$8** with **T4** ($CDCl_3$, 400 MHz, 298 K). See Figure S1 for assignments.

D2) Binding of **T5** *with* ***c*-P10**

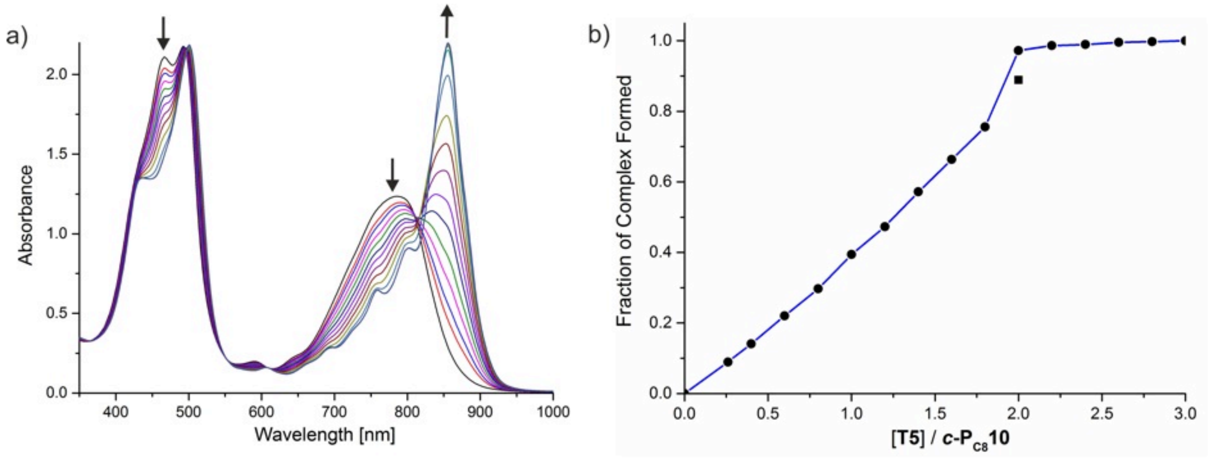

Figure S37. UV/vis/NIR titration of ***c*-P$_{C8}$10** ([***c*-P$_{C8}$10**] = 2.95 × 10^{-6} mol L^{-1}) with **T5** in $CHCl_3$ at 298 K. (a) Changes in absorption upon addition of **T5**. Arrows indicate areas of increasing and decreasing absorption during the titration; (b) The mole fraction of ***c*-P$_{C8}$10•(T5)$_2$** (derived from absorption data at λ = 860 nm) plotted against the molar ration of **T5** / ***c*-P$_{C8}$10**. At the start of the titration the ***c*-P$_{C8}$10** is highly aggregated, and the break-up of these aggregates is slow to reach equilibrium. The titration was carried out with 15 min equilibration time between additions. The isolated square point at 2 equiv of **T5** corresponds to the absorption immediately after addition, before the 15 min equilibration time. The slightly concave shape of the binding curve is attributed to competing aggregation when less than 2 equivalents of the **T5** template is present.

D3) Binding of **T6** *with* ***c*-P8**

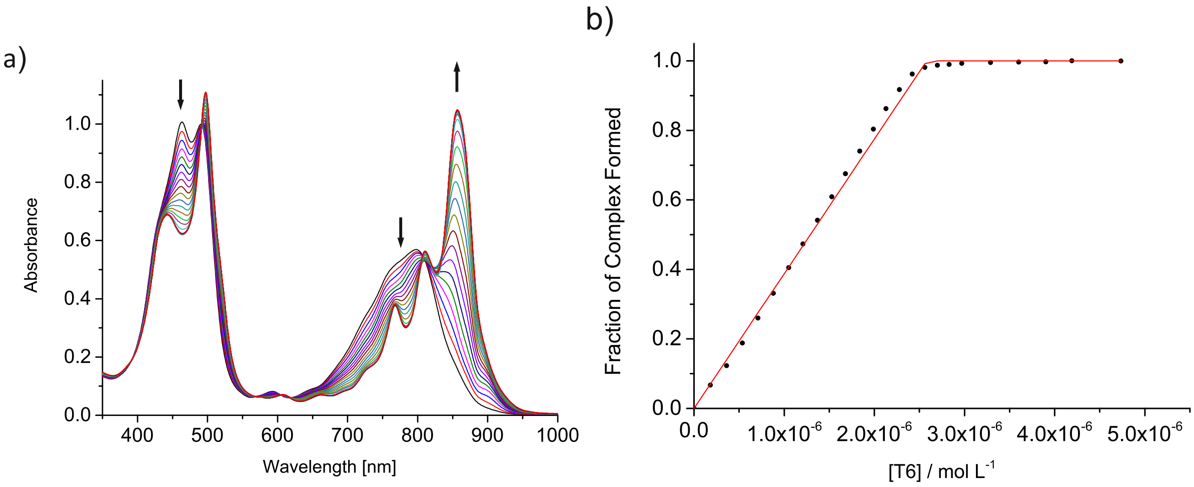

Figure S38. UV/vis/NIR titration of ***c*-P$_{t\text{-Bu}}$8** ([***c*-P$_{t\text{-Bu}}$8**] = 1.29 × 10^{-6} mol L^{-1}) with **T6** in $CHCl_3$ at 298 K. (a) Changes in absorption upon addition of **T6**. Arrows indicate areas of increasing and decreasing absorption during the titration; (b) Binding isotherm (black dots) derived from absorption data at λ = 852 nm and calculated fit (red line). A square end-point is reached at 2:1 **T6**:***c*-P$_{t\text{-Bu}}$8** ratio.

D4) Binding of **T6** *with* ***c*-P10**

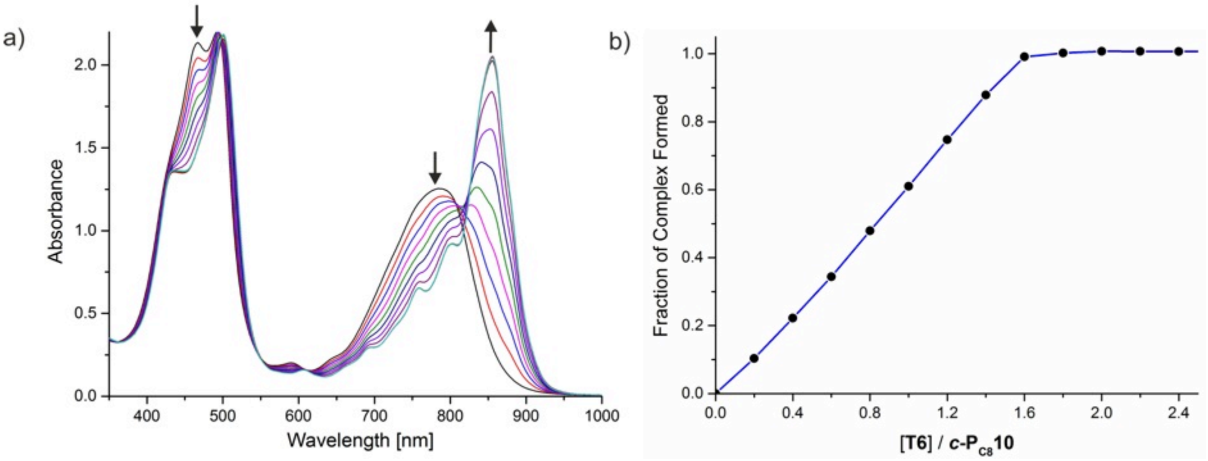

Figure S39. UV/vis/NIR titration of ***c*-P$_{C8}$10** ([***c*-P$_{C8}$10**] = 2.95 × 10^{-6} mol L^{-1}) with **T6** in $CHCl_3$ at 298 K. (a) Changes in absorption upon addition of **T6**. Arrows indicate areas of increasing and decreasing absorption during the titration; (b) The mole fraction of ***c*-P$_{C8}$10•(T6)$_2$** (derived from absorption data at λ = 860 nm) plotted against the molar ration of **T6** / ***c*-P$_{C8}$10**.

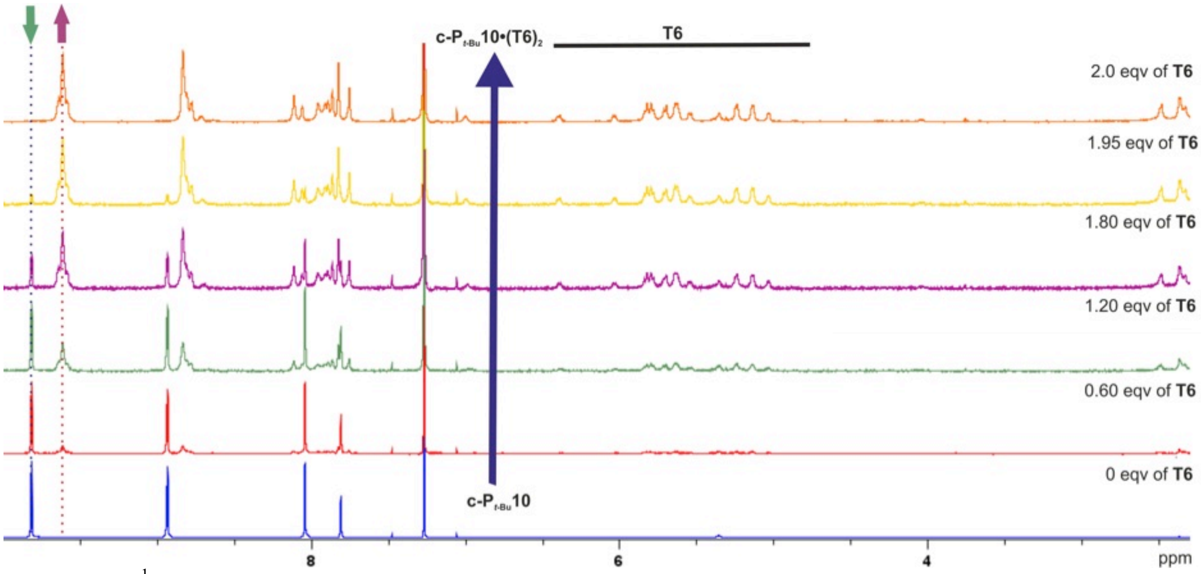

Figure S40. ^{1}H NMR titration of ***c*-P$_{t\text{-}Bu}$10** with **T6** ($CDCl_3$, 500 MHz, 298 K). See Figure S13 for assignments.

D5) Binding of **$Pd(PhCN)_2Cl_2$** *with* ***c*-$P_{t\text{-}Bu}$10•(T6)$_2$**

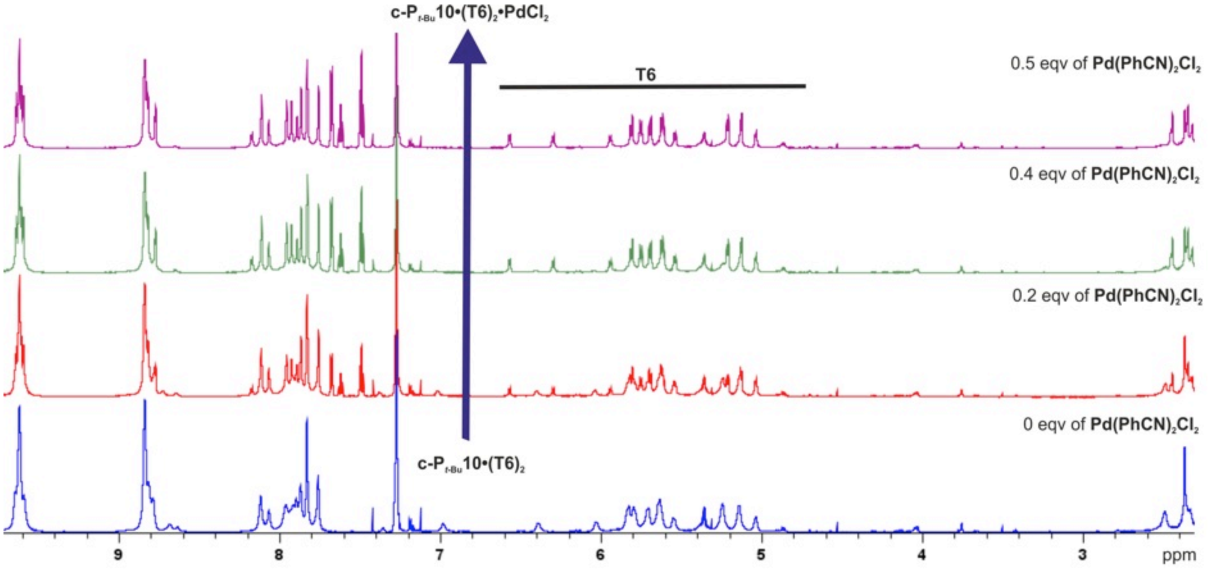

Figure S41. ^{1}H NMR titration of ***c*-$P_{t\text{-}Bu}$10•(T6)$_2$** with $Pd(PhCN)_2Cl_2$ ($CDCl_3$, 700 MHz, 298 K). See Figures S13 and S23 for assignments.

E. EXchange SpectroscopY Experiments

E1) EXSY experiments on ***c*-P$_{t\text{-Bu}}$8•(T6)$_2$**

For the following EXSY experiment, the *ortho* protons on the porphyrin aryl side-groups were selectively irradiated to observe exchange patterns. Identical EXSY experiments were also carried out targeting the template proton ***d*** (Figure 5). Spectra were recorded under various mixing times, ranging from 100 ms to 600 ms.

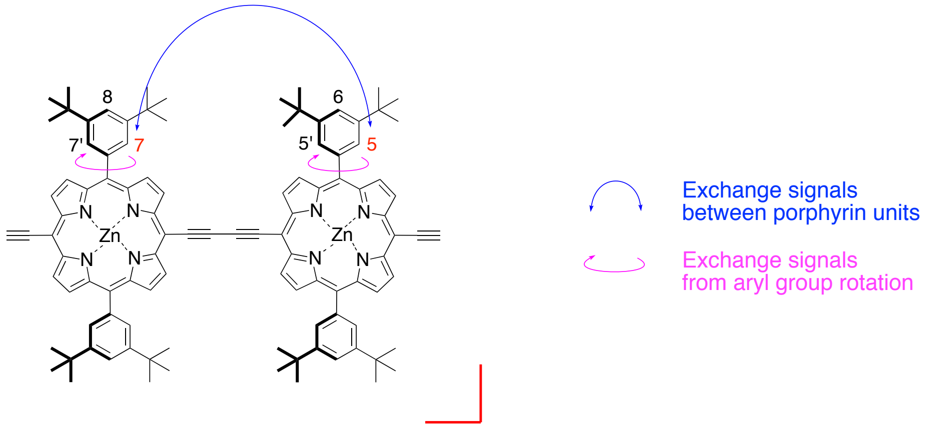

Figure S42. Indications showing the exchange signals arising from the exchange between porphyrin units and the rotations of the aryl groups.

From the EXSY experiment targeting aryl protons ***5*** and *7* on ***c*-P$_{t\text{-Bu}}$8·(T6)$_2$**, there are two types of exchange signals (Figure S42): i) signals corresponding to the rotation of the aryl group (i.e. ***5*** exchanges with ***5′*** and *7* exchanges with *7′*), and ii) signals arising from the movement of the whole porphyrin nanoring (i.e. ***5*** exchanges with *7*; Figure S43b,c). This second type of exchange signal confirms that besides the template, the nanoring itself also moves. Control experiments were carried out on ***c*-P$_{t\text{-Bu}}$8·(T4)$_2$**, and only signals resulting from the aryl group rotation were observed in this case (Figure S43f,g).

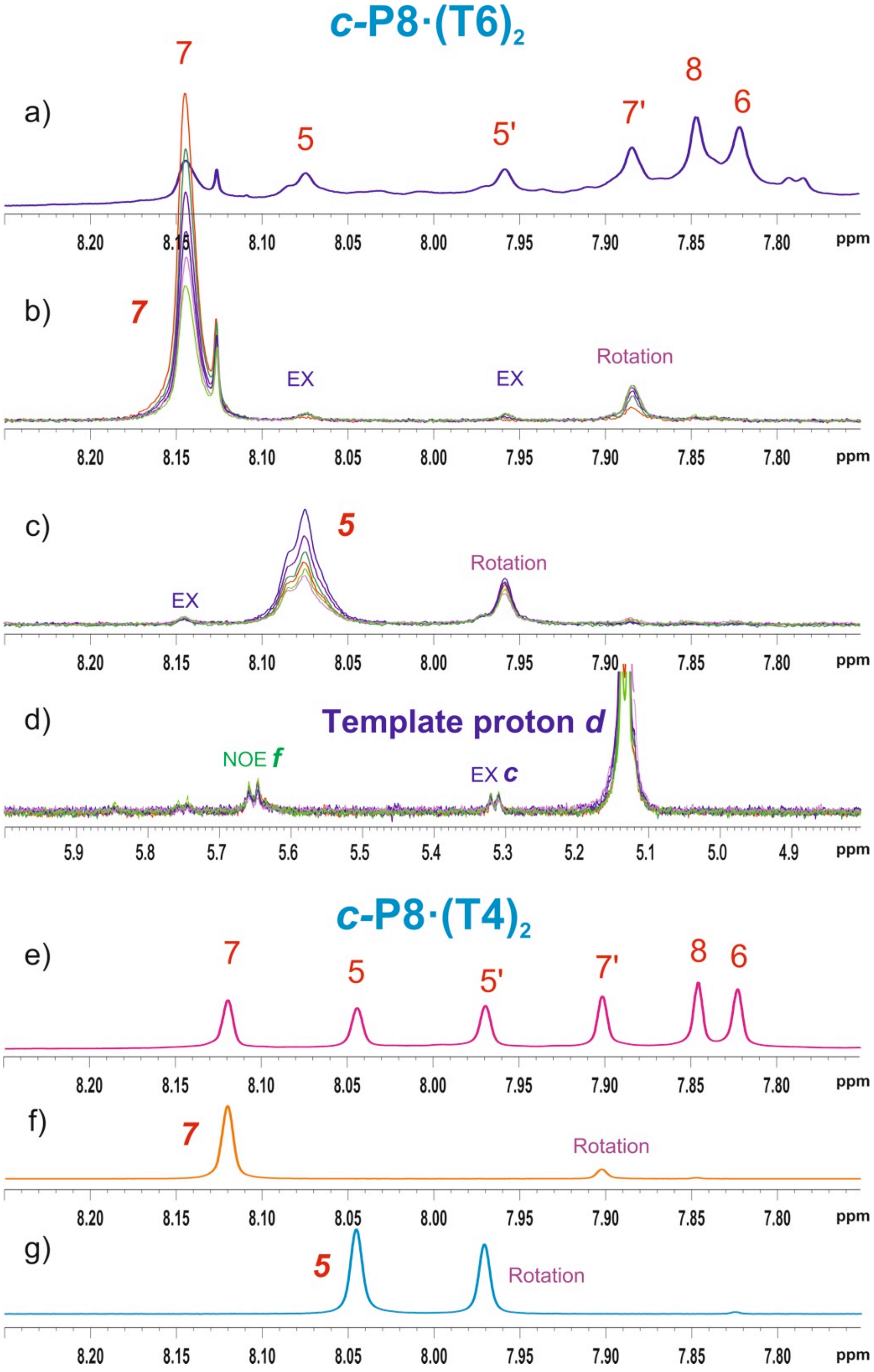

Figure S43. EXSY NMR Spectra ($CDCl_3$, 700 MHz, 298 K, mixing times ranging from 100 ms to 600 ms, indicated by different colors). a) Aryl side-group region of the ^1H NMR spectrum of ***c*-P$_{t\text{-Bu}}$8·(T6)$_2$**; b) overlaid EXSY spectra of ***c*-P$_{t\text{-Bu}}$8·(T6)$_2$** with different mixing times, targeting proton *7*; c) overlaid EXSY spectra of ***c*-P$_{t\text{-Bu}}$8·(T6)$_2$** with different mixing times, targeting proton *5*; d) overlaid EXSY spectra of ***c*-P$_{t\text{-Bu}}$8·(T6)$_2$** with different mixing times, targeting the template proton ***d***; e) Aryl side-group region of the ^1H NMR spectrum of ***c*-P$_{t\text{-Bu}}$8·(T4)$_2$**; f) EXSY spectrum of ***c*-P$_{t\text{-Bu}}$8·(T4)$_2$** targeting proton *7*, mixing time = 400 ms; g) EXSY spectrum of ***c*-P$_{t\text{-Bu}}$8·(T4)$_2$** targeting proton *5*, mixing time = 400 ms.

The intensity of each exchange peak at a given mixing time was divided by the sum of the intensities of the exchange peaks and target peak at that mixing time (i.e. by the total magnetization) to give the normalized intensities plotted in Figure S44. The data were fitted to the kinetic models shown in Figure S44 using the Berkeley Madonna software package

(1993-2001 Robert I. Macey & George F. Oster), giving the rate constants shown in Figure S44. These results show that template rotation (d→c) and porphyrin nanoring rotation (7→5) occur at the same rate (k_1 = 0.13 s^{-1}), while the aryl side-group rotation occurs more rapidly (7→7'; k_2 = 4 s^{-1}; 5→5'; k_3 = 0.4 s^{-1}). The experimental uncertainties in these rate constants are estimated to be 15% for k_1 and k_2, and ca. 40% for k_3. The main source of error in these rate constants comes from the fact that some magnetization is lost by relaxation and through nuclear Overhauser effects. It is interesting that the rate of aryl rotation of 7→7' is much faster than 5→5'; the same effect is also observed in ***c*-P$_{t\text{-Bu}}$8·(T4)$_2$** (Figure S43f,g) which shows that it is unrelated to the caterpillar track motion.

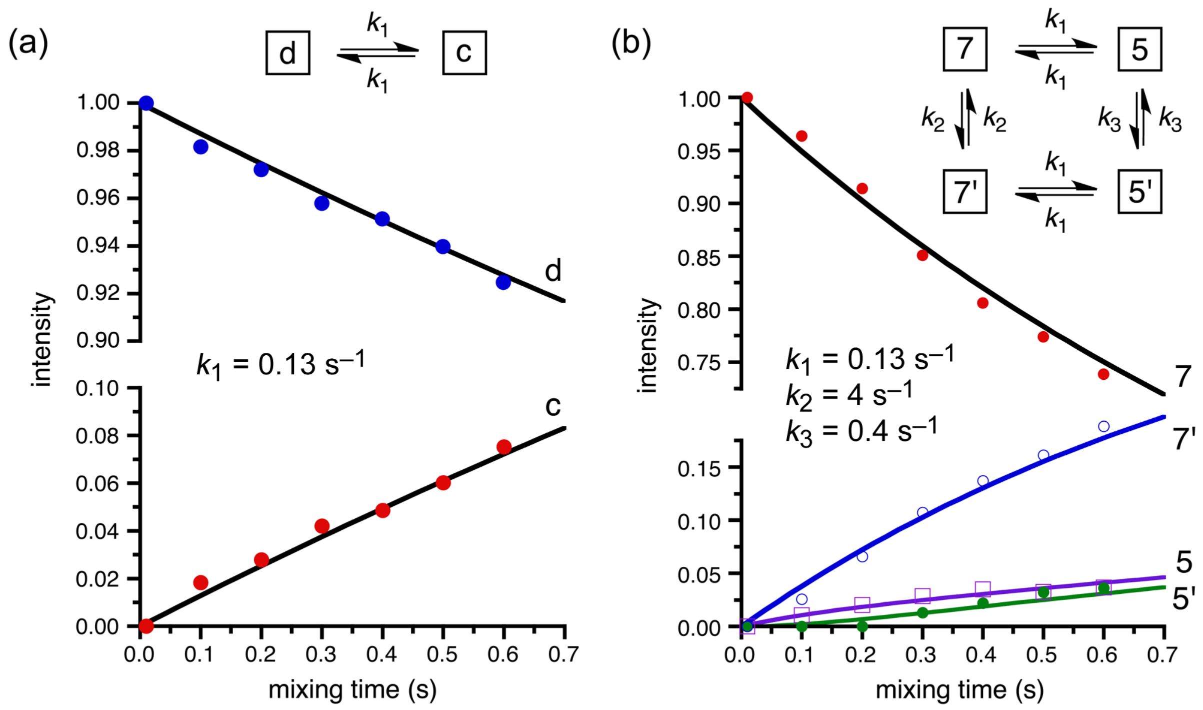

Figure S44. Plot of the normalized integrated exchange peaks against mixing time in ***c*-P$_{t\text{-Bu}}$8•(T6)$_2$** ($CDCl_3$, 700 MHz, 298 K). (a) Data from irradiation of template proton ***d***. (b) Data from irradiation of proton *7*. Curves were fitted to the kinetic models shown using the Berkeley Madonna software package. The experimental data plotted here come from the spectra in Figure S43b,d.

E2) EXSY experiments for ***c*-P$_{t\text{-Bu}}$10•(T6)$_2$** and ***c*-P$_{t\text{-Bu}}$10•(T6)$_2$•PdCl$_2$**

Similar EXSY experiments were also performed on ***c*-P$_{t\text{-Bu}}$10•(T6)$_2$**. Nanoring and template protons were selectively irradiated to observe exchange patterns. In this case, we found that the exchange occurs much faster than in ***c*-P$_{t\text{-Bu}}$8•(T6)$_2$**.

The following section describes our observations when proton ***a'*** is irradiated (Figure 46b). Spectra were recorded for mixing times ranging from 5 ms to 200 ms. As mentioned above, the intensity of each exchange signal was divided by the sum of the intensities of the exchange signals and target peak at that mixing time, to give the normalized intensities plotted in Figure S45. A gradual build-up of exchange signals for protons ***b'***, then ***c'*** and finally ***d'*** was observed. The data were fitted to the kinetic model shown in Figure S45 using the Berkeley Madonna software package, with all six rate constant set to be equal. The good fit to this model confirms that the template undergoes a step-wise rotation within the nanoring rather than dissociating and re-binding in a random orientation.

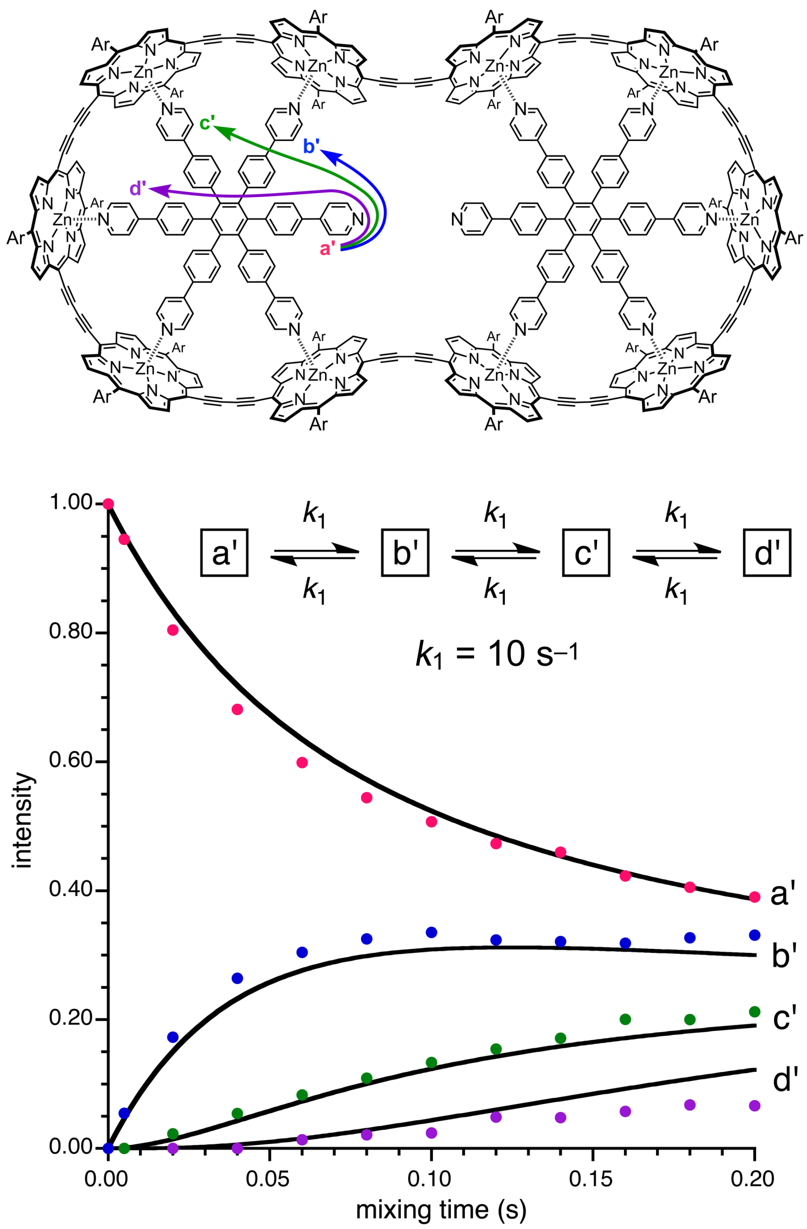

Figure S45. Top: Structure of ***c*-P$_{t\text{-Bu}}$10•(T6)$_2$** and arrows showing the step-wise exchange between template proton ***a′*** and ***b′*/*c′*/*d′***. Bottom: Normalized magnetizations at ***a′***, ***b′***, ***c′*** and ***d′*** plotted against mixing time and fitted to the kinetic model with ${}^{k}{}_{1}$ = 10 s^{-1}. The experimental data plotted here come from the spectra in Figures S46b and S47.

When the sample of ***c*-P$_{t\text{-Bu}}$10•(T6)$_2$** was treated with one equivalent of $PdCl_2(PhCN)_2$, all exchange peaks disappeared even at the longer 200 ms mixing time. While proton ***a′*** in the free ***c*-P$_{t\text{-Bu}}$10•(T6)$_2$** exchanged to ***b′***, then ***c′*** and finally ***d′*** at 200 ms mixing time (Figure S46b), the same EXSY experiment on ***c*-P$_{t\text{-Bu}}$10•(T6)$_2$•PdCl$_2$** only revealed NOE correlations with protons ***a*** and ***a″*** (Figure S46d).

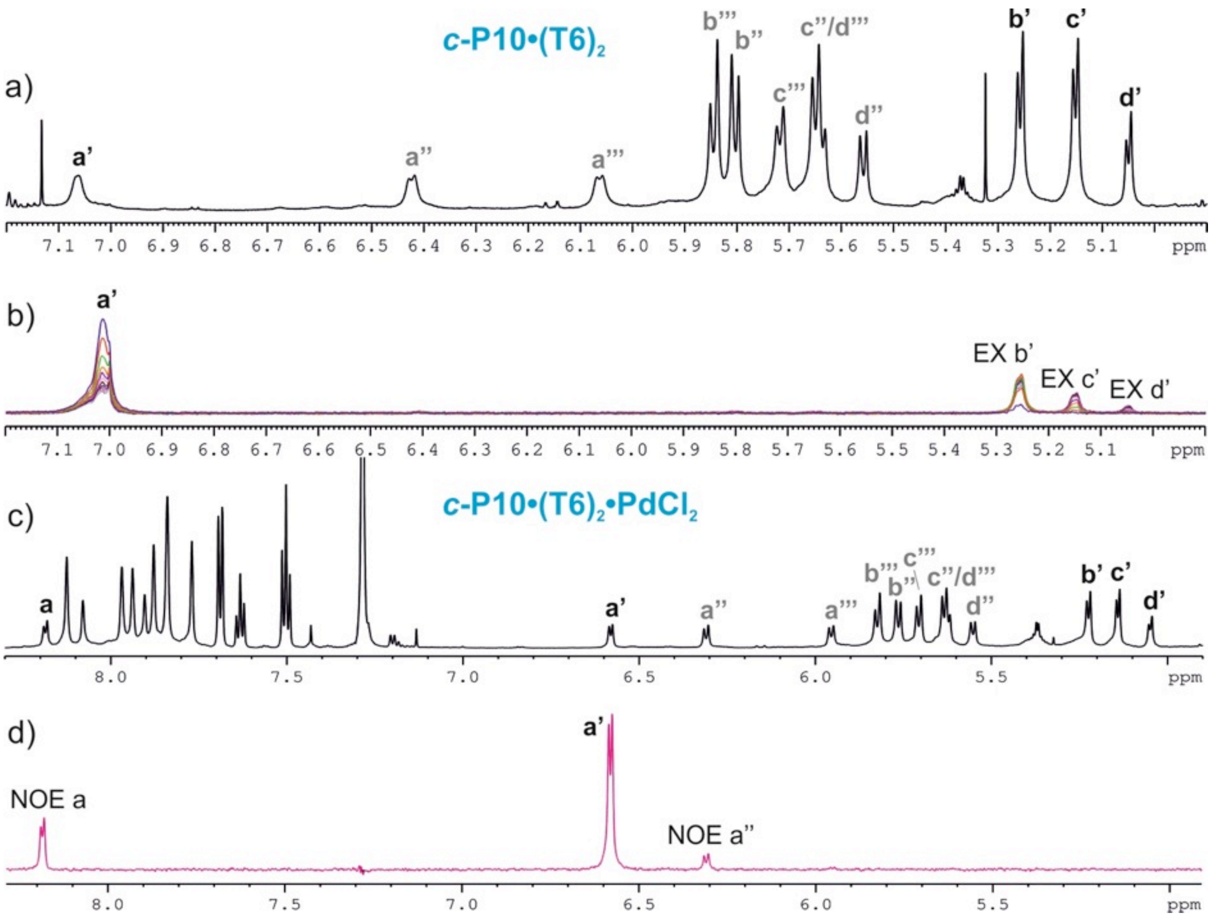

Figure S46. EXSY NMR experiments ($CDCl_3$, 700 MHz, 298 K, mixing times ranging from 5 ms to 200 ms, indicated by different colors). a) **T6** template region of the ^1H NMR spectrum of ***c*-P$_{t\text{-Bu}}$10·(T6)$_2$**; b) overlaid EXSY spectra of ***c*-P$_{t\text{-Bu}}$10·(T6)$_2$** with different mixing times, targeting proton ***a'***; c) **T6** template and aryl side-group region of the ^1H NMR spectrum of ***c*-P$_{t\text{-Bu}}$10·(T6)$_2$·PdCl$_2$**; f) EXSY spectrum of ***c*-P$_{t\text{-Bu}}$10·(T6)$_2$·PdCl$_2$** targeting proton ***a'***, mixing time = 200 ms.

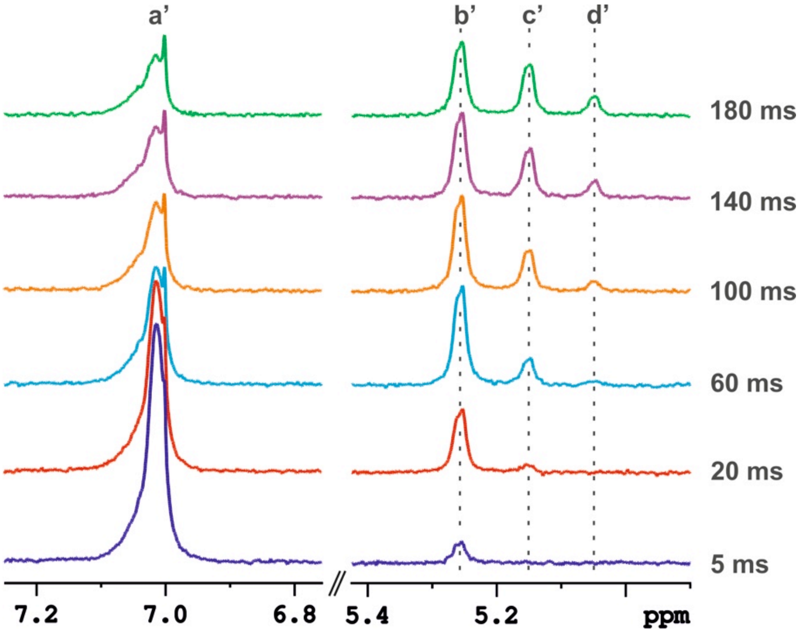

Figure S47. Expansion from Figure S46b showing the sequential growth of the exchange signals for protons ***b'***, ***c'*** and ***d'*** (only selected mixing times are shown for clarity).

F. Small Angle X-ray Scattering (SAXS) analysis of *c*-P$_{t\text{-Bu}}$8•(T4)$_2$ and *c*-P$_{C8}$10•(T5)$_2$

Synchrotron radiation SAXS data were collected using standard procedures on the I22 beamline at the Diamond Light Source (UK) equipped with a photon counting detector. The beam was focused onto the detector placed at a distance of 1.25 m from the sample cell. The covered range of momentum transfer was $0.03 < q < 1.0$ Å^{-1} ($q = 4\pi \sin(\theta)/\lambda$ where 2θ is the scattering angle and λ = 1.00 Å is the X-ray wavelength). The data were normalized to the intensity of the incident beam; the scattering of the solvent was subtracted using an in-house program. To check for radiation damage during the SAXS experiment, the data were collected in 10 successive 60 s frames. All SAXS measurements were performed in toluene at known concentrations (~10^{-4} M) in a solution cell with mica windows (1 mm path length). Simulated scattering curves from molecular models were obtained by fitting to the experimental scattering data using the program CRYSOL.[S7] The program GNOM[S8] was used to calculate pair distribution functions and radii of gyration from experimental and simulated scattering data.

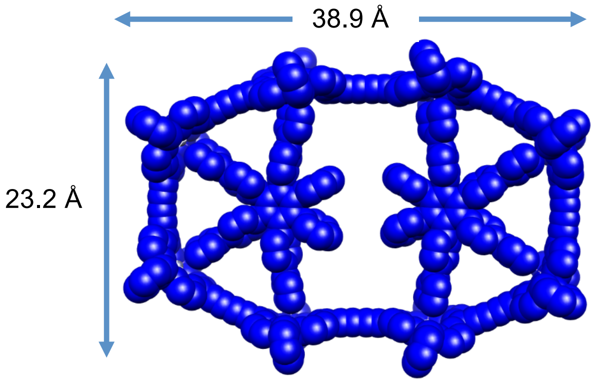

a)

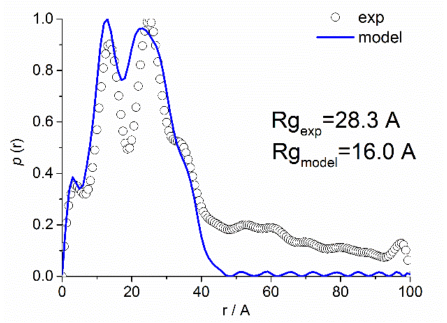

b)

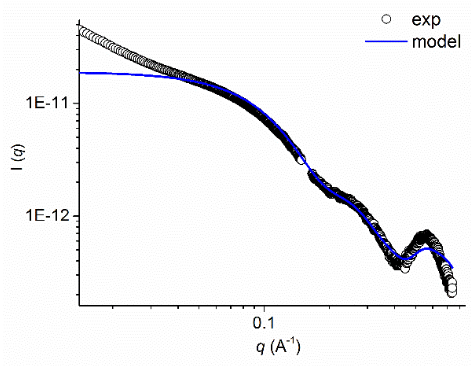

c)

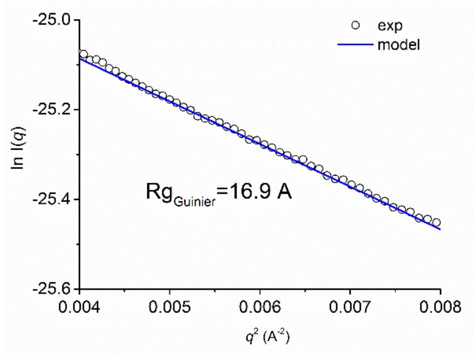

d)

Figure S48. SAXS analysis of the molecular structure of ***c*-P$_{t\text{-Bu}}$8•(T4)$_2$** in solution (toluene, 5.1 mg/mL, 298 K). **a)** Molecular model used for fitting of and comparison with experimental data. The geometry was optimized by molecular mechanics using HyperChem™ with a MM+ force field. **b)** Experimental (black circles) and model based (blue line) pair distribution function (PDF), obtained using the software *Gnom*. **c)** Experimental scattering curve (black circles) plotted on a double log scale with fitted data from the molecular model (blue line). **d)** Guinier analysis obtained from the experimental scattering data at very small angles using the software *Primus*.

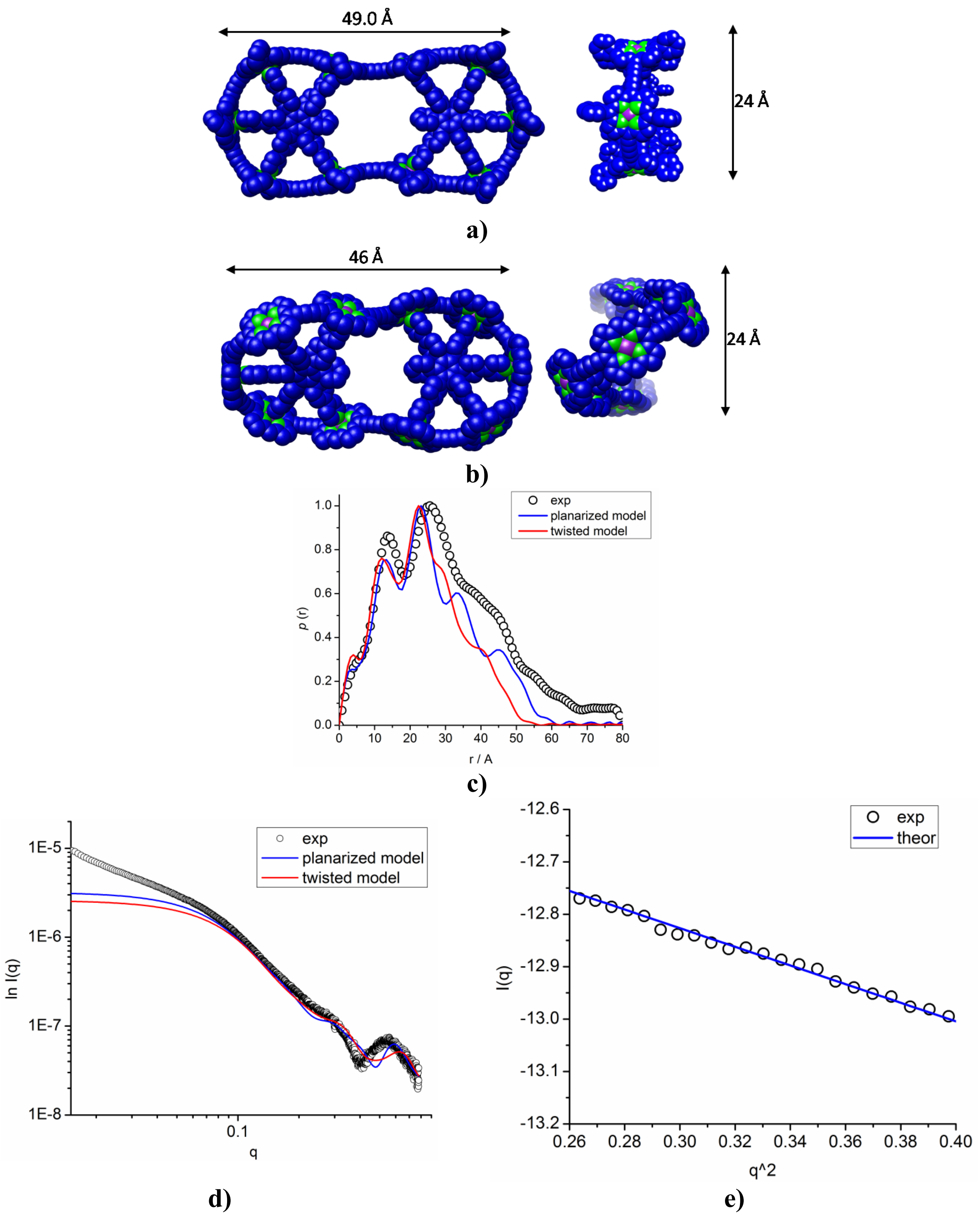

Figure S49. SAXS analysis of the molecular structure of ***c*-P$_{C8}$10•(T5)$_2$** in solution (toluene, 4.7 mg/mL, 298 K). Two molecular models used – planar (a) and twisted (b) for fitting of and comparison with experimental data. The geometry was optimized by molecular mechanics using HyperChem^{TM} with a MM+ force field. **c)** Experimental (black circles) and model based (blue line-planarized model, red line – twisted model) pair distribution function (PDF), obtained using the software *Gnom*. **d)** Experimental scattering curve (black circles) plotted on a double log scale with fitted data from the molecular model (blue line). **d)** Guinier analysis obtained from the experimental scattering data at very small angles using the software *Primus*.

G. Calculated geometry of *c*-P10•(T6)$_2$•PdCl$_2$

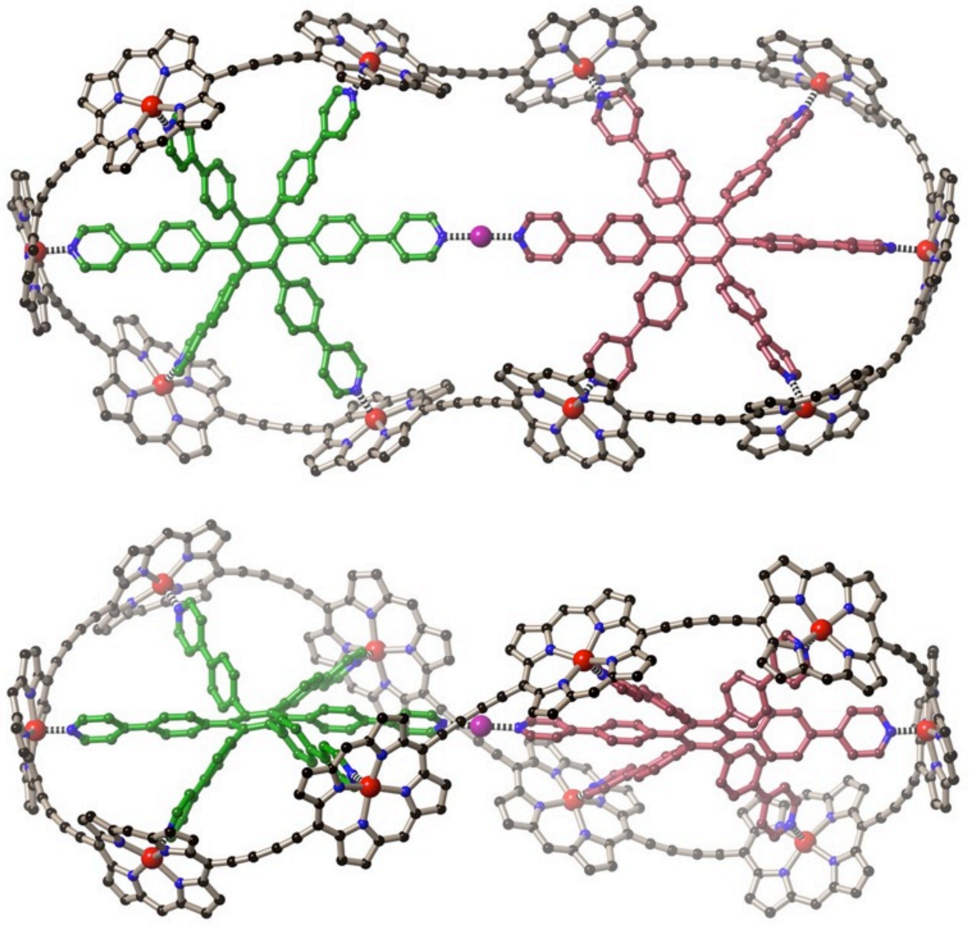

Figure S50. Two orthogonal views of the calculated structure of ***c*-P10•(T6)$_2$•PdCl$_2$**. The geometry was optimized by molecular mechanics using HyperChem^{TM} with a MM+ force field. Aryl side groups were not included in the calculation, and the PdCl$_2$ unit was modeled as a single metal atom. Hydrogen atoms are omitted from the diagram for clarity.

H. References

[S1] J. D. Wilkinson, G. Wicks, A. Nowak-Król, Ł. Łukasiewicz, C. Wilson, M. Drobijev, A. Rebane, D. T. Gryko, H. L. Anderson, *J. Mater. Chem. C* **2014**, *2*, 6802–6809.

[S2] M. Hoffmann, J. Kärnbratt, M.-H. Chang, L. M. Herz, B. Albinsson, H. L. Anderson, *Angew. Chem. Int. Ed.* **2008**, *47*, 4993–4996.

[S3] H. J. Hogben, J. K. Sprafke, M. Hoffmann, M. Pawlicki, H. L. Anderson, *J. Am. Chem. Soc.* **2011**, *133*, 20962–20969.

[S4] K. E. Maly, E. Gagnon, T. Maris, J. D. Wuest, *J. Am. Chem. Soc.* **2007**, *129*, 4306-4322.

[S5] M. Hoffmann, C. J. Wilson, B. Odell, H. L. Anderson, *Angew. Chem. Int. Ed.* **2007**, *46*, 3122–3125.

[S6] M. C. O'Sullivan, J. K. Sprafke, D. V. Kondratuk, C. Rinfray, T. D. W. Claridge, A. Saywell, M. O. Blunt, J. N. O'Shea, P. H. Beton, M. Malfois, H. L. Anderson, *Nature* **2011**, *469*, 72–75.

[S7] D. I. Svergun, C. Berbato, M. H. J. Koch, *J. Appl. Cryst.* **1995**, *28*, 768–773.

[S8] D. I. Svergun, *J. Appl. Cryst.* **1992**, *25*, 495–503.
